# Lithium normalizes ASD-related neuronal, synaptic, and behavioral phenotypes in DYRK1A-knockin mice

**DOI:** 10.1038/s41380-024-02865-2

**Published:** 2024-12-05

**Authors:** Junyeop Daniel Roh, Mihyun Bae, Hyosang Kim, Yeji Yang, Yeunkeum Lee, Yisul Cho, Suho Lee, Yan Li, Esther Yang, Hyunjee Jang, Hyeonji Kim, Hyun Kim, Hyojin Kang, Jacob Ellegood, Jason P. Lerch, Yong Chul Bae, Jin Young Kim, Eunjoon Kim

**Affiliations:** 1 Center for Synaptic Brain Dysfunctions, Institute for Basic Science (IBS), Daejeon, 34141 Korea; 2 Department of Biological Sciences, Korea Advanced Institute for Science and Technology (KAIST), Daejeon, 34141 Korea; 3 Digital Omics Research Center, Korea Basic Science Institute, Cheongju, 28119 Korea; 4 Korea Institute of Drug Safety & Risk Management, Anyang, 14051 Korea; 5 Department of Anatomy and Neurobiology, School of Dentistry, Kyungpook National University, Daegu, 41940 Korea; 6 Department of Anatomy and BK21 Graduate Program, Biomedical Sciences, College of Medicine, Korea University, Seoul, 02841 Korea; 7 Bertis Inc, Gwacheon, 13840 Korea; 8 Division of National Supercomputing, KISTI, Daejeon, 34141 Korea; 9 Mouse Imaging Centre, Hospital for Sick Children, Toronto, Ontario, M5T 3H7 Canada; 10 Bloorview Research Institute, Holland Bloorview Kids Rehabilitation Hospital, Toronto, Ontario, M4G 1R8 Canada; 11 Wellcome Centre for Integrative Neuroimaging, University of Oxford, Oxford, Oxfordshire, OX39DU UK

**Keywords:** Neuroscience, Drug discovery

## Abstract

Dyrk1A deficiency is linked to various neurodevelopmental disorders, including developmental delays, intellectual disability (ID) and autism spectrum disorders (ASD). Haploinsufficiency of *Dyrk1a* in mice reportedly leads to ASD-related phenotypes. However, the key pathological mechanisms remain unclear and human *DYRK1A* mutations remain uncharacterized in mice. Here, we generated and studied *Dyrk1a*-knockin mice carrying a human ASD patient mutation (Ile48LysfsX2; Dyrk1a-I48K mice). These mice display severe microcephaly, social and cognitive deficits, dendritic shrinkage, excitatory synaptic deficits, and altered phospho-proteomic patterns enriched for multiple signaling pathways and synaptic proteins. Early chronic lithium treatment of newborn mutant mice rescues the brain volume, behavior, dendritic, synaptic, and signaling/synapse phospho-proteomic phenotypes at juvenile and adult stages. These results suggest that signaling/synaptic alterations contribute to the phenotypic alterations seen in Dyrk1a-I48K mice, and that early correction of these alterations by lithium treatment has long-lasting effects in preventing juvenile and adult-stage phenotypes.

## Introduction

DYRK1A (dual-specificity tyrosine-phosphorylation-regulated kinase 1 A) is a serine/threonine kinase that has been implicated in Down syndrome, a human condition caused by trisomy of chromosome 21. Previous studies of DYRK1A using animal models of Down syndrome mainly focused on phenotypes associated with its overexpression, such as developmental delay, learning and memory impairments, and neuronal and synaptic deficits [1–3]. Meanwhile, recent human genetic studies found that reduced (not increased) *DYRK1A* expression is associated with various neurodevelopmental brain dysfunctions, including developmental delay, microcephaly, autism spectrum disorder (ASD), intellectual disability, and seizures [4–8]. The clinical importance of *DYRK1A* downregulation is supported by reports indicating that *DYRK1A* mutations are found in 0.1–0.5% of individuals with ASD and/or intellectual disability [4–6, 9–12] and ~0.5% of those with developmental disorders [13]. As such, studying relevance of Dyrk1a to the pathophysiology of ASD in human-mimicking animal models is called for.

Previous studies on global *Dyrk1a*-haploinsufficient mice reported neurodevelopmental and ASD-related phenotypes, including reduced viability, developmental delay, microcephaly, behavioral deficits, and neuronal and synaptic alterations [14–20]. These results provide strong face validity for *Dyrk1a* haploinsufficiency and suggest potential mechanisms underlying *Dyrk1a*-related mouse phenotypes. However, no published study has completely described their phenotypic rescue, related causal mechanisms, or in-depth characterization of mice carrying a *DYRK1A* mutation identified in humans.

Here we generated and characterized *Dyrk1a*-knockin mice carrying a human *DYRK1A* mutation (Ile48LysfsX2; termed Dyrk1a-I48K mice). These mice display severe microcephaly, dendritic shrinkage, suppressed excitatory synaptic transmission, and social impairments that are associated with phospho-proteomic changes in signaling pathway-related and synaptic proteins. Early chronic treatment of newborn mutant mice with lithium rescues the brain, behavioral, dendritic, synaptic, and phospho-proteomic phenotypes at juvenile and adult stages. Our work highlights that early lithium treatment triggers mechanisms through which multiple signaling and synaptic proteins exert long-lasting rescue effects in this model.

## Materials and Methods

### Animals

To generate Dyrk1A-I48K knock-in (KI) mice, two nucleotides were removed to replace the 48th isoleucine residue of exon 3 with lysine and introduce a frame shift-generated stop codon (I48K fsX2). A neomycin cassette flanked by FRT (flippase recognition target) elements responsive to the flippase enzyme was added to the intron following exon 3. A subsequent crossing with protamine flp mice, which express flippase in the male germ line (Protamine-Flp mice), removed the cassette and left a residual segment that could be detected by polymerase chain reaction (PCR). Animals were fed *ad libitum* and housed under a 12-h light/dark cycle (light phase from 01:00 am to 13:00 pm). Genotypes of Dyrk1a-KI HT mice were determined by PCR using the following primer pairs: wild-type [WT] allele (413 bp): 5’-557GAG AAA GAG AGC TGT TTG CCT TCC G-3’ (forward) and 5’-CAA GCA GTT ACA558AGT TCC AGG CTC C-3’ (reverse); knockout (KO) allele (589 bp), 5-GAG AAA559GAG AGC TGT TTG CCT TCC G-3’ (forward) and 5’-ATG CTG AGA AGG CAG560GTA GGT AAG G-3’ (reverse); floxed allele (323 bp), 5’-GAG ATG GCG CAA CGC561AAT TAA TG-3’ (forward) and 5’-CAA GCA GTT ACA AGT TCC AGG CTC C-3’562(reverse). Mice were weaned at postnatal day 21 (P21), and mixed-genotype littermates of the same gender were housed together until experiments. Only male mice were used for adult behavior tests, whereas both male and female mice were used for pup and juvenile tests, as indicated in the figure legends. For the majority of this study, we employed a heterozygous KI line in the C57BL/6 J background (Dyrk1a-I48KfsX2^KI/+^ or KI). Homozygous Dyrk1a-KI mice were generated by shifting the genetic background of Dyrk1a-KI mice from pure C57BL/6 J to a hybrid 129 Sv;C57BL/6 J (50:50) genetic background (termed homozygous/HM Dyrk1a-KI mice). The two original mouse lines (C57BL/6 J and 129 Sv) were maintained independently for at least five generations before being crossed and/or used to produce the mice for experiments.

### Ethics approval

Mice were bred and maintained at the mouse facility of Korea Advanced Institute of Science and Technology (KAIST) according to the Animal Research Requirements of KAIST. All experimental procedures were conducted in accordance to the guidelines of Animal Research Requirements on the protection of animals used for scientific purposes (Laboratory Animal Act Law 19918, Jan 2, 2024, Korea) and KAIST Animal Welfare Guidelines, and were approved by the Committee of Animal Research and Ethics at KAIST (KA2023-093-v1).

### MRI measurement of mouse brain

Mice were anesthetized and intracardially perfused with 10 ml of 0.1 M PBS containing 10 U/ml heparin (PPC, cat#C504805) and 2 mM ProHance (Gadolinium contrast agent, Bracco Diagnostics, cat#111181) followed by 10 ml of 4% paraformaldehyde/PFA (Cedarlane cat#15710) containing 2 mM ProHance. After perfusion, mice were decapitated. The brain and remaining skull structures were incubated in 4% PFA  +  2 mM ProHance overnight at 4 °C then transferred to 0.1 M PBS containing 2 mM ProHance and 0.02% sodium azide for at least seven days before MRI scanning. For the anatomical MRI scans a T2 weighted, three-dimensional fast spin-echo sequence was used, with a cylindrical acquisition of k-space, and with a TR of 350 ms, and TEs of 12 ms per echo for six echoes, field of view of 20 × 20 × 25 mm^3^ and matrix size = 504 × 504 × 630 giving an image with 0.040 mm isotropic voxels. Total imaging time for this sequence was ~14 h.

### Electrophysiology

Ex-vivo electrophysiological experiments were performed to evaluate excitatory/inhibitory synaptic transmission and extracellular field population recordings. More detailed information on electrophysiology methods is available in Supplementary Information.

### Transcriptomic analysis

RNA-Seq analysis was performed using i) P21 HT/heterozygous-Dyrk1a-KI mice, ii) P60 HT-Dyrk1a-KI mice, and their WT counterparts. Detailed information on RNA sample preparation and DEG analyses GSEA analyses are described in Supplementary Information.

### Proteomic analysis

Total and PTM proteomic analyses by LC-MS/MS were performed using i) P21 HT-Dyrk1a-KI mice, ii) P60 HT-Dyrk1a-KI mice, and iii) P60-HM/homozygous-Dyrk1a KI mice and their WT counterparts. Detailed information on the sample preparation, LC-MS/MS analysis and proteome identification are available in Supplementary Information.

### Behavioral tests

All adult behavioral tests were performed with age-matched male WT and Dyrk1a-KI mice (2–5 months). Pup and juvenile tests were conducted using both male and female mice as indicated in the figure legends. All behavioral experiments were carried out during light-off periods, except for the home cage nesting behavioral and Laboras tests. Rest periods of at least 1 day were given between tests. Unless otherwise specified, all data were analyzed using EthoVision XT 10 (Noldus). More details on individual behavioral test schemes are listed in the Supplementary Information.

### Pharmacological rescue

For pharmacological rescues of behaviors, lithium was indirectly delivered to pups through mammary milk by providing dams with lithium carbonate (Merck #255823) at a concentration of 600 mg/liter in drinking water. Dams and pups were treated with lithium starting 3 days after birth through mother’s milk until they were able to drink the lithium-containing directly up to P21. At P21, mice were weaned and co-housed in cages containing 3–6 mice with the same genetic background. After weaning, mice were fed normal drinking water. Drug-administered dams were not returned to mating cages. We did not directly measure the levels of lithium carbonate and its metabolites in the sera of newborn mice, but a previous study employing the same drug treatment strategy has demonstrated that lithium can reach the blood of newborn mice via mother’s milk and reported detailed concentrations of serum fluoxetine and its metabolite during and after the treatment [21–23].

### Data acquisition and statistical analysis

Behavioral analyses were performed in a double-blind manner. All data are expressed as mean values with standard error of mean (SEM). All statistical analyses were performed using the GraphPad Prism software (version 7.0). The statistical significance of values is indicated in the figure panels as follows: *p < 0.05, **p < 0.01, and ***p < 0.001. Detailed statistical results are presented in Supplementary Table 1.

## Results

### Characterization of *Dyrk1a*-I48K-knockin mice

A protein-truncating mutation in the *DYRK1A* gene (Ile48LysfsX2) was reported in a human autistic individual with the following symptoms: 1) microcephaly, 2) intellectual disability, 3) anxiety, 4) ASD-related social deficits, 5) impaired speech, 6) stereotypic behavior, and 7) febrile seizures [6, 10]. To determine if this mutation could yield related deficits in mice, we generated *Dyrk1a*-knockin mice carrying the Ile48Lys (or I48K) mutation (Supplementary Fig. 1a).

This protein-truncating mutation is located near the N-terminus of the protein; its introduction led to the removal of the whole protein with the truncated N-terminal peptide (48 amino acids) being undetectable, as shown by the ~50% decrease seen in the DYRK1A protein levels of heterozygous *Dyrk1a*-knockin mice (termed ‘Dyrk1a-KI mice’ hereafter) by immunoblot analyses using both N- and C-terminal antibodies (Supplementary Fig. 1b, c; Supplementary Table 1). Consistent with previous results [14–16], homozygous Dyrk1a-KI mice were embryonically lethal.

The embryonic lethality of homozygous Dyrk1a-KI mice led us to assess the temporal expression patterns of *Dyrk1a* during embryonic and postnatal stages. In situ hybridization detected *Dyrk1a* mRNAs in various brain regions, including the olfactory bulb, cortex, hippocampus, and cerebellum **(**Supplementary Fig. 1d), similar to the previous results [24, 25]. DYRK1A protein levels were relatively high during embryonic and early postnatal stages, then decreased to adult levels at ~ postnatal week 4 (or ~ postnatal day/P28) (Supplementary Fig. 1e). DYRK1A protein levels were comparable in different brain regions at P14 (Supplementary Fig. 1f).

Dyrk1a-KI mice displayed developmental delay, as evidenced by decreased body weight; this was more obvious in males than females at juvenile and adult stages (Supplementary Fig. 1g, h). Brain weight and area and brain/body weight ratios were also decreased in male Dyrk1a-KI mice at postnatal weeks 3 and 8, corresponding to juvenile and adult stages, respectively (Supplementary Fig. 1i). Gross morphology of the Dyrk1a-KI brain was largely normal, as determined by immunostaining for DAPI (cell body marker), NeuN (neurons), S100β (astrocytes), and neurofilament M (axons) (Supplementary Fig. 1j, k). These results collectively suggest that *Dyrk1a* deletion leads to neurodevelopmental delays that can be detected at juvenile and adult stages.

### Severe microcephaly in Dyrk1a-KI mice

**Fig. 1:**
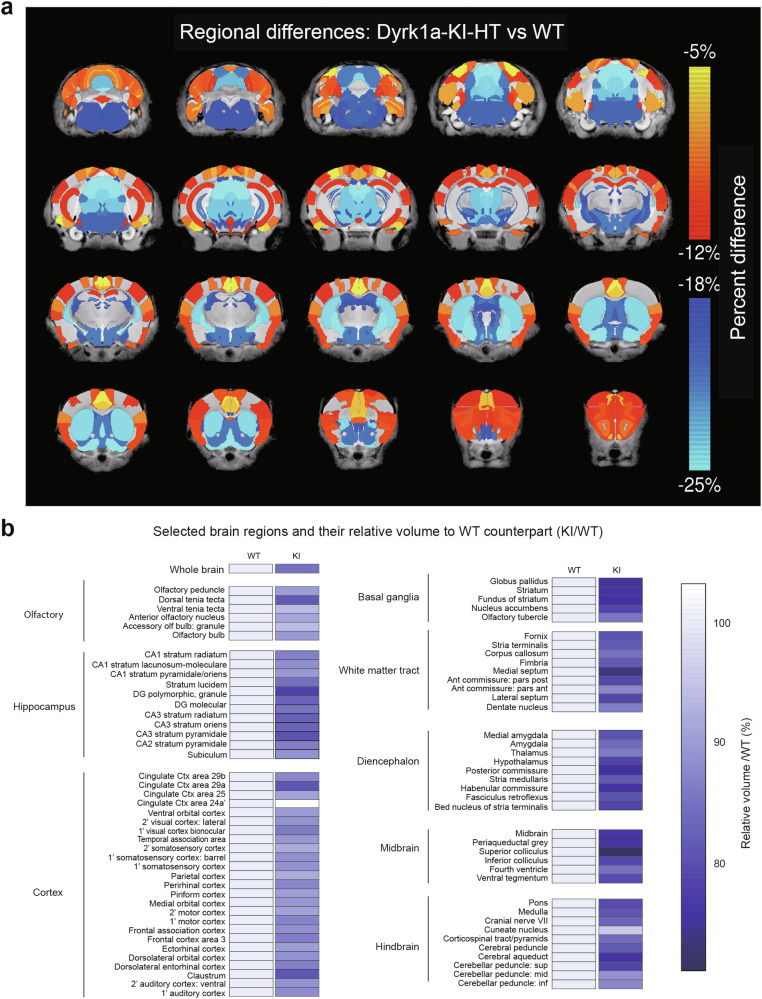
Severe microcephaly in Dyrk1a-KI mice. **a** Heatmaps showing volumetric changes (largely decreases) in various brain regions of Dyrk1a-KI mice (8 weeks, male) compared with WT mice, as assessed using MRI. (*n* = 9 mice [WT, KI], Student’s *t*-test). **b** Detailed analysis of brain regions exhibiting reduced volumes in Dyrk1a-KI versus WT mice. The widespread volumetric decreases in various brain regions are further highlighted by the observation that only one subregion shows a small volumetric increase (cingulate cortex area 24a’; ~3%; indicated by a white box). (*n* = 9 mice [WT, KI], Student’s t-test).

Given that the autistic individual with the DYRK1A-I48K mutation displayed microcephaly [6, 10] and our Dyrk1a-KI mice showed decreases in their brain weights and areas (Supplementary Fig. 1i), we further analyzed brain volumes using magnetic resonance imaging (MRI). MRI analyses of 8-week-old Dyrk1a-KI mouse brains indicated that there was global microcephaly, with the strongest reductions (5–25%) seen in brain regions of the basal ganglia, diencephalon, and midbrain (Fig. 1a).

The specific brain sub-regions that exhibited stronger volumetric decreases included the hippocampal dentate gyrus, cingulate cortex, globus pallidus, nucleus accumbens, medial septum, medial amygdala, hypothalamus, and superior colliculus (Fig. 1b). These results collectively suggest that the Dyrk1a-I48K mutation in mice leads to severe microcephaly.

### Behavioral abnormalities in Dyrk1a-KI mice

To test whether the developmental delay and microcephaly observed in Dyrk1a-KI mice are associated with behavioral abnormalities, we subjected the mutant mice to a battery of behavioral tests. We used male mice for these assays because body weights were significantly decreased only in male mice.

Adult-stage Dyrk1a-KI mice (2–5 months) displayed largely normal locomotor activity in the open-field and Laboras tests, which measure movements in novel and familiar environments, respectively (Supplementary Fig. 2a, b). These mice also showed normal anxiety-like behaviors in the open-field, elevated plus-maze, and light-dark tests, and normal motor coordination in the rotarod test (Supplementary Fig. 2a, c–e).

**Fig. 2:**
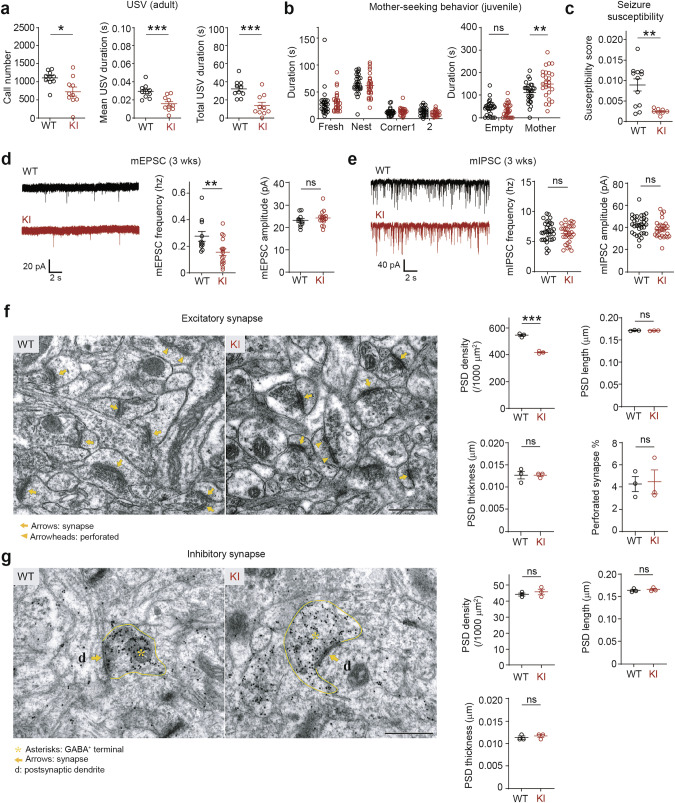
Behavioral deficits, decreased seizure susceptibility, and reduced excitatory synaptic transmission and density in Dyrk1a-KI mice. **a** Significant suppression of ultrasonic vocalizations (USVs) in Dyrk1A-KI mice (2–3 months; male) versus WT mice, as shown by the number and mean duration of each USV and the total duration of all USVs. (*n* = 10 mice [WT], 10 [KI], Student’s t-test, Mann-Whitney U test). **b** (right) Increased time spent with a reunited mother in juvenile Dyrk1a-KI versus WT mice (P22; male) (*n* = 27 [WT], 25 [KI], two-way ANOVA). (left) The mutant mice showed normal levels of olfactory function in the session performed immediately before the mother-reunification session, as shown by their preference for bedding from the previous nest relative to fresh bedding or empty corners. **c** Decreased seizure susceptibility in Dyrk1a-KI mice (2–4 months; male) in the pentylenetetrazolium (PTZ)-induced seizure test. (*n* = 12 [WT], 8 [KI], Student’s *t*-test). **d** Decreased frequency but not amplitude of mEPSCs in CA1 hippocampal pyramidal neurons (P19–21; male). (*n* = 11 neurons from 3 mice [WT], 16, 3 [KI], Mann-Whitney U-test, Student’s *t*-test). **e** Normal levels of mIPSCs in CA1 hippocampal pyramidal neurons (P21–22; male). (*n* = 33, 6 [WT], 29, 6 [KI], Student’s *t*-test). **f** Decreased density of excitatory postsynaptic densities (PSDs) but no change in their length, thickness, or perforation in the hippocampal CA1 region of Dyrk1a-KI mice (P21; male). Arrows indicate putative excitatory synapses with PSDs apposed to presynaptic axon terminals. Scale bar, 500 nm. **g** Normal density, length, and thickness of inhibitory PSDs in the hippocampal CA1 region of Dyrk1a-KI mice (P21; male). Arrows indicate putative inhibitory synapses with PSDs that are apposed to GABA-immunopositive axon terminals. Scale bar, 500 nm. (*n* = 3 mice [WT, KI], Student’s t-test). Significance is indicated as * ( < 0.05), ** ( < 0.01), *** ( < 0.001), or ns (not significant).

In social/repetitive behavioral tests, adult Dyrk1A-KI mice emitted markedly fewer ultrasonic vocalizations (USVs) during courtship behaviors, although their social interactions were normal in the three-chamber test (Fig. 2a; Supplementary Fig. 2f), reminiscent of the speech delay and social deficits observed in the human individual who carries the DYRK1A-I48K mutation [6, 10]. Repetitive behavior was also altered, as shown by decreased climbing, although self-grooming and rearing were normal (Supplementary Fig. 2g–i).

Learning and memory were moderately suppressed in adult Dyrk1A-KI mice, as evidenced by a decrease in spatial fear memory retrieval at 24 h but not at 7 days (Supplementary Fig. 3a, b). In contrast, cued fear conditioning and spatial learning and memory were normal in the Morris water maze (Supplementary Fig. 3c, d). These results are also reminiscent of the intellectual disability observed in the DYRK1A-I48K human individual [6, 10].

Juvenile Dyrk1a-KI mice spent more time with their reunited mother after a 30 min separation (Fig. 2b), suggestive of anxiety-like behavior and similar to the behavior seen in the DYRK1A-I48K human individual. However, these mice showed locomotor activity and social/repetitive behaviors within the normal range (Supplementary Fig. 3e-g). Dyrk1a-KI pups emitted normal levels of USVs when separated from their mothers (Supplementary Fig. 3h), indicative of normal anxiety-like behavior.

Collectively, these results suggest that Dyrk1a-KI mice display various behavioral abnormalities in social, repetitive, and cognitive behavioral domains at both juvenile and adult stages.

### Decreased seizure susceptibility and excitatory synaptic density in the Dyrk1a-KI hippocampus

ASD frequently involves an imbalance of excitation/inhibition in brain functions [26–28]. We thus tested for altered excitability of the Dyrk1a-KI brain by subjecting the mutant mice to the pentylenetetrazolium (PTZ)-induced seizure test. Dyrk1a-KI mice showed markedly decreased levels of PTZ-induced brain excitation (Fig. 2c).

To test if this hypo-excitation of the brain involved synaptic alterations, we measured miniature excitatory and inhibitory postsynaptic currents (mEPSCs and mIPSCs, respectively) in CA1 pyramidal neurons of the hippocampus, as this brain region exhibited detectable *Dyrk1a* expression and volumetric decreases in Dyrk1A-KI mice (Supplementary Fig. 1d, f; Fig. 1). Our results revealed that Dyrk1a-KI CA1 pyramidal neurons displayed a decrease in the frequency but not amplitude of mEPSCs at ~P21 (Fig. 2d). In contrast, mIPSCs were normal in both frequency and amplitude in mutant neurons (Fig. 2e). These findings indicate that the mutant mice exhibited a decrease in the ratio of excitatory to inhibitory synaptic transmission.

Electron microscopic analyses revealed that the excitatory synapse density was decreased in the mutant CA1 region: There were fewer postsynaptic densities (PSDs) apposed to presynaptic axon terminals, although the PSDs were normal in their length, thickness, and perforation (a measure of maturation) (Fig. 2f). In contrast, inhibitory PSDs were not altered in density, length, or thickness (Fig. 2g). These results suggest that excitatory synaptic density is decreased in the mutant hippocampus.

Interestingly, other measures of excitatory synaptic function were largely normal: There was no change in basal AMPA receptor-mediated synaptic transmission (input-output curve) or the ratio of evoked NMDA to AMPA receptor-mediated currents (NMDA/AMPA ratio), with a moderate change in presynaptic release (paired-pulse ratio) (Supplementary Fig. 4a–c). There was also no change in measures of synaptic plasticity, including high-frequency stimulation long-term potentiation (HFS-LTP), theta-burst stimulation LTP (TBS-LTP), low-frequency stimulation long-term depression (LFS-LTD), and metabotropic glutamate receptor-dependent LTD (mGluR-LTD) (Supplementary Fig. 4d–g).

These results collectively suggest that the Dyrk1a-KI mutation in mice decreases hippocampal excitatory synaptic density without affecting inhibitory synaptic density or other measures of excitatory synaptic function (basal transmission, NMDA receptor function, and synaptic plasticity), thereby decreasing the ratio of excitatory-to-inhibitory synaptic transmission.

### Early lithium treatment rescues dendritic arborization, synaptic transmission and density, brain size, and social behavior in Dyrk1a-KI mice

**Fig. 3:**
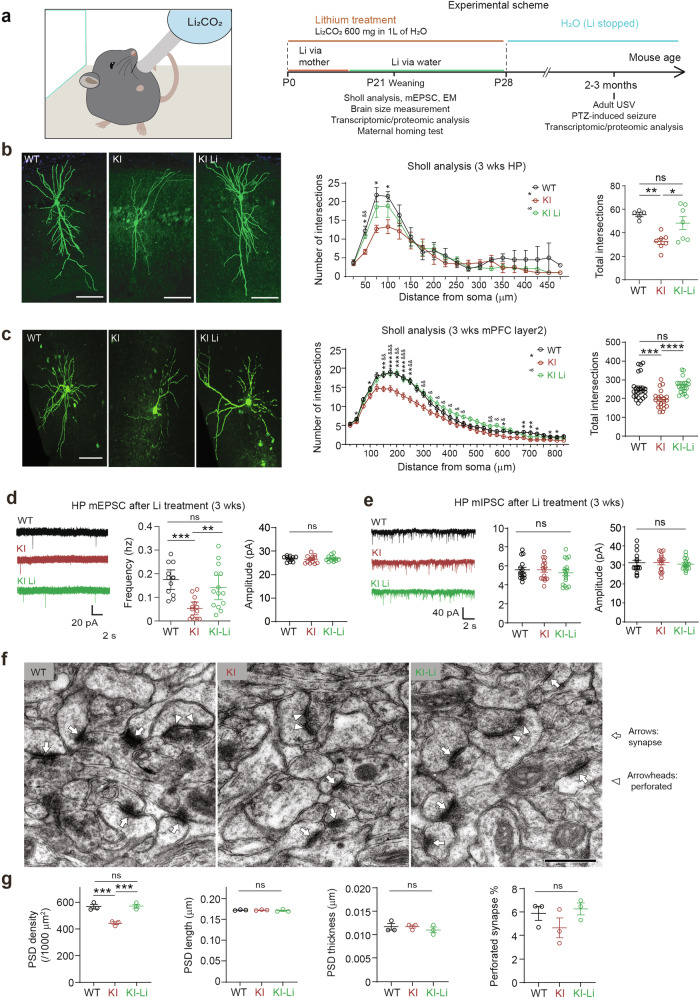
Early lithium treatment rescues dendritic branching and synaptic transmission and density in Dyrk1a-KI mice. **a** Schematic depiction of early postnatal chronic lithium treatment and subsequent measurements of dendritic arborization (Sholl analysis), excitatory synaptic transmission (mEPSCs), excitatory synaptic density, brain size, proteomic profiles, and mother-seeking behavior in the maternal homing test at ~P21, and social communication (adult USVs), seizure susceptibility, and proteomic profiles at 2–3 months. Lithium carbonate (600 mg/L H_2_O) was given to pups via the mother’s milk up until weaning at ~P21. At ~P21, lithium-treated mice were randomly selected for Sholl analysis (hippocampus and mPFC), mEPSC recording, EM analysis of excitatory synaptic density (PSD density and shape), brain area measurement, and proteomic analyses. The remaining mice continued to receive lithium treatment via their drinking water until ~P28; they were thereafter given normal drinking water, and behaviors (USV social communication and proteomic profiles were assessed at ~2–3 months. **b** Normalized dendric arborization in hippocampal (HP) CA1 pyramidal neurons in lithium-treated Dyrk1a-KI mice ( ~ P21; male), as determined by Sholl analysis. Scale bar, 100 μm. (*n* = 5 neurons from 3 mice [HP-WT], 7, 3 [HP-KI; Li-untreated], and 7, 3 [HP-KI-Li], one-way ANOVA). **c** Normalized dendric arborization in mPFC (medial prefrontal cortex) prelimbic layer 2 pyramidal neurons (**c**) in lithium-treated Dyrk1a-KI mice ( ~ P21; male), as determined by Sholl analysis. Scale bar, 100 μm. (*n* = 25, 3 [mPFC-WT], 21, 3 [mPFC-KI; Li-untreated], and 22, 3 [mPFC-KI-Li], one-way ANOVA). **d**, **e** Normalized frequency of mEPSCs in hippocampal CA1 pyramidal neurons in lithium-treated Dyrk1a-KI mice ( ~ P21) with no effects on mIPSCs. (*n* = 12 neurons from 3 mice [mEPSC-WT], 13, 3 [mEPSC-KI], 14, 3 [mEPSC-KI-Li], 15, 3 [mIPSC-WT], 16, 3 [mIPSC-KI], 17, 3 [IEPSC-KI-Li], one-way ANOVA). **f**, **g** Normalized density of excitatory synapses (PSDs apposed to axon terminals) without effects on PSD length, thickness, or perforation in the hippocampal CA1 area of lithium-treated Dyrk1a-KI mice ( ~ P21). (*n* = 3 mice [WT], 3 [KI], 3 [KI-Li], one-way ANOVA).

**Fig. 4:**
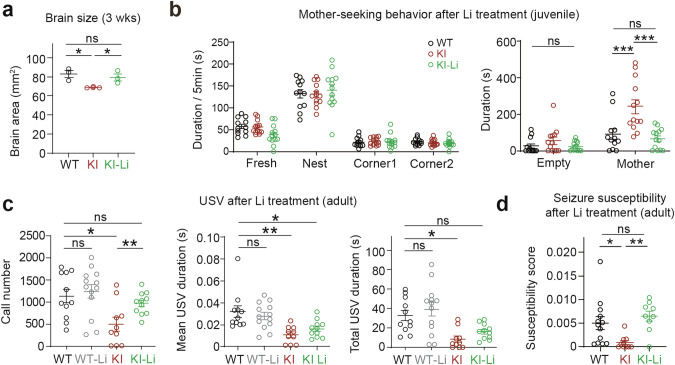
Early lithium treatment rescues brain size and behaviors in juvenile and adult Dyrk1a-KI mice. **a** Normalized brain size in lithium-treated Dyrk1a-KI mice ( ~ P21). Whole-brain areas were measured using top-down brain images. (*n* = 3 mice [WT, KI, and KI-Li], one-way ANOVA). **b** Normalized mother-seeking behavior in lithium-treated Dyrk1a-KI mice (P21), as shown by time spent with reunited mother in the maternal homing test. (*n* = 12 [WT], 13 [KI], and 13 [KI-Li], one-way ANOVA). **c** Partially normalized courtship USVs in lithium-treated Dyrk1a-KI mice (2–3 months), as shown by full normalization of USV call number and partial normalization of total USV duration. (*n* = 11 [WT], 13 [WT-Li], 10 [KI], and 11 [KI-Li], two-way ANOVA). **d** Normalized seizure susceptibility in lithium-treated Dyrk1a-KI mice (2–3 months), as shown by time spent with reunited mother in the maternal homing test. (*n* = 13 [WT], 10 [KI], and 9 [KI-Li], one-way ANOVA). Significance is indicated as * ( < 0.05), ** ( < 0.01), *** ( < 0.001), or ns (not significant).

We hypothesized that the suppressed dendritic arborization and decreased excitatory synaptic density in Dyrk1a-KI mice (Fig. 2) may represent key mechanisms underlying the phenotypes of Dyrk1a-KI mice. To test this hypothesis, we set out to treat Dyrk1a-KI mice with lithium, bipolar disorder medication known to exert pleiotropic neurodevelopmental, neuroprotective, and neuro-synaptic effects through multiple mechanisms, including GSK3β inhibition [29–36]. We utilized a chronic treatment during the first 3–4 postnatal weeks because: 1) Dyrk1a protein levels remain high until ~P21 (Supplementary Fig. 1d), 2) behavioral phenotypes were detectable at juvenile stages (Fig. 2b; Supplementary Fig. 2 and 3), 3) excitatory synaptic deficits were observed at juvenile stages (Figs. 2d–g and 4) early corrections of pathological mechanisms can elicit long-lasting effects [37]. Specifically, newborn heterozygous Dyrk1a-KI pups (~ P0) were treated with lithium through the mother’s milk until weaning (~ P21) [21–23], and dendritic arborization, synaptic transmission and density, brain size, proteomic profiles, and mother-seeking behavior were assessed at ~P21 (Fig. 3a). After being weaned at ~P21, another set of Dyrk1a-KI pups continued to receive lithium treatment through their drinking water until ~P28. At ~2–3 months of age, social communication (adult USVs) and seizure susceptibility were measured, and proteomic profiles were obtained.

The early lithium-treated Dyrk1a-KI mice showed normalized dendritic arborization in hippocampal CA1 pyramidal neurons and prefrontal prelimbic pyramidal neurons at ~P21, compared with those of untreated Dyrk1a-KI mice, as determined by Sholl analysis (Fig. 3b, c). In addition, hippocampal neurons of lithium-treated Dyrk1a-KI mice showed a normalized frequency of mEPSCs at ~P21 without effects on mIPSCs (Fig. 3d, e). Consistently, the lithium treatment normalized excitatory synaptic density, as determined by the EM analysis of PSD, without affecting PSD morphology (Fig. 3f).

Remarkably, early lithium-treated Dyrk1a-KI mice showed a normal brain size at ~P21, lacking the microcephaly seen in their untreated counterparts (Fig. 4a). Lithium also restored mother-seeking behavior at ~P21 (Fig. 4b) and courtship USVs (partial) and seizure susceptibility at 2–3 months (Fig. 4c, d).

These results collectively suggest that early postnatal and chronic lithium treatment of Dyrk1a-KI mice rescues multiple Dyrk1a-KI phenotypes (dendritic arborization, synaptic transmission and density, brain size, and behavior) both immediately and long after the treatment, and has long-lasting effects in preventing behavioral phenotypes from appearing at adult stages.

### Posttranslational modifications in early lithium-treated Dyrk1a-KI mice at ~ P21

To better understand the mechanisms underlying the early lithium-dependent rescue of Dyrk1a-KI phenotypes, we sought to analyze Dyrk1a-KI transcriptomes and proteomes. We used whole-brain samples because Dyrk1a is widely expressed in the brain, and brain volumetric and neuromorphological changes were observed in various brain regions.

The RNA-Seq results from Dyrk1a-KI whole brains at P21 indicated small numbers of differentially expressed genes (DEGs) but, in gene set enrichment analysis (GSEAs) [38, 39], strong increases in synapse-related gene expressions and “reverse-ASD” transcriptomic patterns (changes opposite to those occurring in ASD) (Supplementary Fig. 5a–d; Supplementary Table 2). These results suggest compensatory transcriptomic changes to normalize excitatory synaptic function at P21. The P60 transcriptome showed reverse-ASD patterns associated with upregulated splicing-related genes and downregulated oligodendrocyte-related genes (Supplementary Fig. 5e–h; Supplementary Table 3), suggestive of compensatory changes that occur through other (or not-synaptic) mechanisms.

We next analyzed total proteomes using WT and Dyrk1a-KI mice at ~P21 and ~P60 and found small numbers of differentially expressed proteins (total-DEPs; *p* < 0.05 + FC > 1.2; total 13 for P21 [up 8 and down 5]; total 13 for P60 [up 5 and down 8]) (Supplementary Fig. 6a–d; Supplementary Table 4). There were no overlapping DEPs at P21 and P60. This suggests that the Dyrk1a-KI mutation induces small changes in total protein levels at ~P21 and ~P60.

We next attempted to analyze Dyrk1a-KI proteomes with a focus on posttranslational modifications (PTMs), which are known to modulate aspects of protein stability, function, localization, interaction, and stability [40, 41]. To this end, we treated WT and Dyrk1a-KI mice with vehicle/lithium during the first three postnatal weeks, analyzed PTM-differentially expressed phosphopeptides (DEPPs), determined baseline differences between vehicle-treated WT mice and vehicle-treated Dyrk1a-KI mice, and identified a subset of baseline differences (vehicle-treated WT/Dyrk1a-KI) that were no longer significantly different between lithium-treated Dyrk1a-KI and vehicle-treated WT mice (termed as ‘lithium-rescued PTM-DEPPs’). We further compared PTM-DEPPs obtained at ~P21 versus ~P60 to examine the immediate and long-term effects of early lithium treatment.

The baseline comparison of vehicle-treated WT and vehicle-treated Dyrk1a-KI mice identified a large number of P21 PTM-DEPPs (*p* < 0.05 + FC > 1.2; 766 total, 336 upregulated (‘up’) and 430 downregulated (‘down’)) (Supplementary Fig. 7a; Supplementary Table 5). Given the small numbers of total DEPs mentioned above (Supplementary Fig. 6a, b), these PTM changes seem to mainly reflect changes in protein phosphorylation levels rather than total protein levels. Many of the PTM-DEPPs with stronger changes (*p* < 0.05 + FC > 2.0) were among the synaptic proteins listed in the SynGO database [42]; these included Dock4 (dedicator of cytokinesis 4]), Elavl2 (ELAV-like RNA-binding protein 2), Dagla (diacylglycerol lipase alpha), Prkcd (protein kinase C delta), Prr7 (proline-rich 7), and Map2 (microtubule-associated protein 2) (Supplementary Fig. 7b).

DAVID analyses using the KEGG database [43] indicated that the PTM-DEPPs (766 peptides) were enriched for various signaling pathways, including the insulin, cAMP, oxytocin, AMPK, ErbB, thyroid hormone, gap junction, and autophagy pathways (Supplementary Fig. 7c). DAVID analyses using the gene ontology (GO) database [44] indicated that the PTM-DEPPs were enriched for ‘postsynaptic density’ and ‘protein binding’ (Supplementary Fig. 7c). Consistently, the PTM-DEPPs were found to be enriched for SynGO proteins/functions (21–27%) (Supplementary Fig. 7d). These results suggest that the P21 PTM-DEPPs may affect various signaling pathways and synaptic proteins to induce the dendritic/synaptic deficits observed in juvenile Dyrk1a-KI mice.

**Fig. 5:**
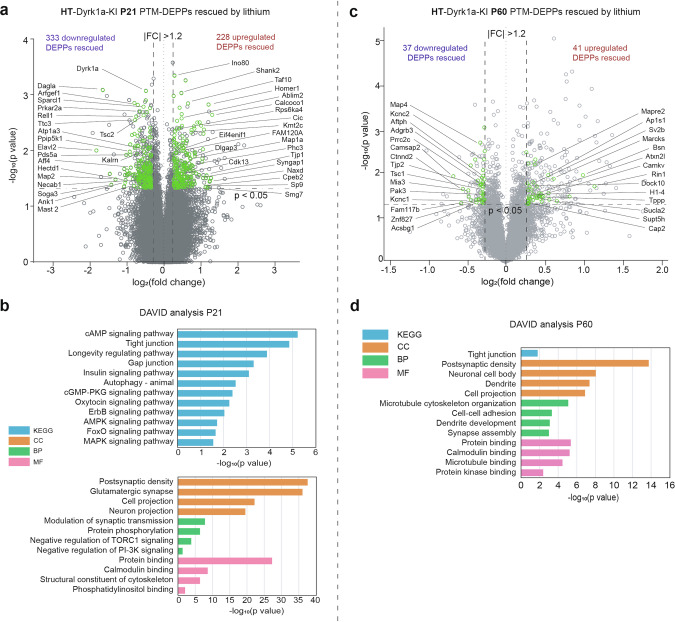
Rescue of P21 and P60 PTM-DEPPs by early lithium treatment of Dyrk1a-KI mice. **a** Volcano plot presentation of PTM-DEPPs at ~P21 that are significantly altered in the baseline condition (vehicle-treated WT and vehicle-treated heterozygous Dyrk1a-KI) but are rescued by early chronic lithium treatment (P0–21) and thus are no longer significantly different (*p* > 0.05) between lithium-treated Dyrk1a-KI mice and vehicle-treated WT mice (termed as ‘lithium-rescued PTM-DEPPs’). The rescued PTM-DEPPs are highlighted as green dots (vehicle-treated WT vs. lithium-treated Dyrk1a-KI mice) among those showing the baseline difference (gray dots; vehicle-treated WT vs. vehicle-treated Dyrk1a-KI mice). (*n* = 3 mice [WT-Veh, KI-Veh, and KI-Li]). **b** DAVID-KEGG/gene ontology (GO) analyses of the lithium-rescued P21 and P60 PTM-DEPPs (up and down pooled). **c** A volcano plot showing early (P0–28) lithium-rescued ~P60 PTM-DEPPs (green dots; no longer significantly different between lithium-treated Dyrk1a-KI mice and vehicle-treated WT mice) overlaid onto the baseline ~P60 PTM-DEPPs (gray dots; vehicle-treated WT vs. vehicle-treated Dyrk1a-KI mice). (*n* = 3 mice [WT-Veh, KI-Veh, and KI-Li]). **d** DAVID-KEGG/gene ontology (GO) analyses of the lithium-rescued P60 PTM-DEPPs (up and down pooled).

We next compared PTM-DEPPs from P21 vehicle-treated WT mice and lithium-treated Dyrk1a-KI mice, highlighting the lithium-rescued PTM-DEPPs in volcano plots of the baseline difference between vehicle-treated WT mice and vehicle-treated Dryk1a-KI mice (Fig. 5a**;** Supplementary Fig. 8a; Supplementary Table 5). DAVID-KEGG analyses indicated the lithium-rescued PTM-DEPPs (561 total; 228 up and 333 down) are enriched for ~80% of the signaling pathways that were identified from the baseline difference analysis (8 out 10 pathways, namely the insulin, cAMP, oxytocin, AMPK, gap junction, longevity, ErbB, and autophagy pathways) (Fig. 5b). DAVID-GO analyses indicated that the lithium-rescued PTM-DEPPs were enriched for ‘postsynaptic density’ and ‘protein binding’ (Fig. 5b), and therefore resembled the DAVID-GO enrichment pattern at baseline (Supplementary Fig. 7c). The lithium-rescued PTM-DEPPs were enriched for SynGO proteins/functions (24–31%) (Supplementary Fig. 8b), further resembling the pattern seen at baseline (Supplementary Fig. 7d). Notably, the lithium-rescued SynGO PTM-DEPPs included Elavl2 (S221) and Kalrn (S488), whose decreased phosphorylations were restored to WT levels after lithium treatment (Fig. 5b). The functions of these phosphorylations remain unclear, but Elavl2 is detected at synapses and regulates synapse assembly [45, 46] and dendritic arborization [47]. In addition, Kalrn (Kalirin), a Rho guanine nucleotide exchange factor (Rho-GEF) implicated in neurodevelopmental disorders, including schizophrenia [48], regulates synaptic functions and dendritic arborization [49].

These results collectively suggest that early chronic lithium treatment of Dyrk1a-KI mice rescues the PTM patterns of various signaling and synaptic proteins seen immediately after weaning (~ P21).

### Posttranslational modifications in early lithium-treated Dyrk1a-KI mice at ~ P60

When PTM-DEPPs were analyzed at ~P60 to assess the long-term effects of early lithium treatment (P0–P28), the baseline comparison of vehicle-treated WT and vehicle-treated Dyrk1a mice revealed many P60 PTM-DEPPs (*p* < 0.05 + FC > 1.2; 306 total, 220 up and 86 down) (Supplementary Fig. 9a; Supplementary Table 6). Some synaptic proteins exhibited strongly upregulation of their P60 PTM-DEPPs (*p* < 0.05 + FC > 2.0), including SynGO proteins (e.g., Shank3 and Shank2), Psd3 (pleckstrin and sec3 domain-containing 3), Kif1a (kinesin family member 1 A), Crk (CRK proto-oncogene, adaptor protein), Pura (purine-rich element binding protein A), Kcnma1 (potassium calcium-activated channel subfamily M alpha 1), Kif21a (kinesin family member 21 A), and Aak1 (AP2-associated kinase 1) (Supplementary Fig. 9b). In DAVID-KEGG analyses, P60 PTM-DEPPs were enriched for certain synapse-related pathways, including the ‘glutamatergic synapse’, ‘GABAergic synapse’, and ‘synaptic vesicle cycle’ pathways (Supplementary Fig. 9c). This result differed from those observed at P21, when various signaling pathways were more strongly enriched (Supplementary Fig. 7c). DAVID-GO analyses showed that P60 PTM-DEPPs were enriched for ‘postsynaptic density’, ‘postsynaptic density’, and ‘protein binding’ (Supplementary Fig. 9c), which was similar to the results obtained from P21 PTM-DEPPs (Supplementary Fig. 5c). Surprisingly, SynGO analyses revealed that P60 PTM-DEPPs showed a notable synaptic enrichment for SynGO proteins/functions ( ~ 46%/38%) (Supplementary Fig. 9d) that was ~2-fold greater than that seen for P21 PTM-DEPPs (Supplementary Fig. 7d). These results suggest that P60 PTM-DEPPs differ from P21 PTM-DEPPs: The former are less enriched for signaling pathways but more strongly enriched for synaptic proteins.

We next identified P60 PTM-DEPPs in vehicle-treated WT mice versus lithium-treated Dyrk1a-KI mice and compared them to the baseline PTM-DEPPs to identify lithium-rescued PTM-DEPPs (Fig. 5c; Supplementary Fig. 8c; Supplementary Table 6). The P60 lithium-rescued PTM-DEPPs (78 total, 41 up and 37 down) included various synaptic proteins, including Bsn (bassoon presynaptic cytomatrix protein), Ap1s1 (adaptor-related protein complex 1 subunit sigma 1), Dock10 (dedicator of cytokinesis 10), Sv2b (synaptic vesicle glycoprotein 2B), Marcks (myristoylated alanine rich protein kinase C substrate), Cap2 (cyclase-associated actin cytoskeleton regulatory protein 2), Kcnc1 (potassium voltage-gated channel subfamily C member 1), Ctnnd2 (catenin delta 2), Pak3 (p21 (RAC1)-activated kinase 3), and Tsc1 (TSC complex subunit 1) (Supplementary Fig. 8c). DAVID-KEGG/GO analyses revealed that the lithium-rescued PTM-DEPPs were strongly enriched for synapse-related functions (i.e., ‘postsynaptic density’) (Fig. 5d). The lithium-rescued PTM-DEPPs were strongly enriched for SynGO proteins/functions (Supplementary Fig. 8d). There was no overlap between P21 and P60 lithium-rescued PTM-DEPPs.

Lastly, homozygous (rather than heterozygous) Dyrk1a-KI mice at ~P60 were analyzed for PTM-DEPPs. Homozygous Dyrk1a-KI mice could be born in a hybrid background (C57/BL6J x 129 Sv), but at a very low Mendelian ratio (1:1:0.015 for WT/heterozygous/homozygous rather than 1:2:1). Thus, they could not be used to examine other phenotypes. This analysis identified numerous PTM-DEPPs (*p* < 0.05 + FC > 1.2; 223 total, 152 up and 71 down) (Supplementary Fig. 10a; Supplementary Table 7). The PTM-DEPPs identified as having stronger changes (*p* < 0.05 + FC > 1.2) included not only synaptic proteins but also mTOR and other signaling proteins (Supplementary Fig. 10b). DAVID and SynGO analyses supported these results (Supplementary Fig. 10c, d). The findings differed from those obtained in heterozygous Dyrk1a-KI mice, where the main changes were observed in synaptic but not signaling proteins. This indicates that there may be distinct gene-dosage effects.

Together, our results suggest that: 1) P60 PTM-DEPPs are enriched more for synaptic proteins than signaling proteins, and thus show a pattern that differs from that of P21 PTM-DEPPs; 2) early lithium treatment of Dyrk1a-KI mice preferentially rescues synaptic protein PTMs relative to signaling protein PTMs in the baseline difference long after the treatment (i.e., at ~P60); and 3) early lithium treatment may rescue signaling protein PTMs at ~P21 to rescue synaptic protein PTMs at ~P60.

### Functional characterization of lithium-rescued PTM-DEPPs in Dyrk1a-KI mice

We next attempted to further understand the mechanisms underlying the lithium-rescued PTM-DEPPs. We tested if the activity of GSK3β, known to be inhibited by lithium and regulate synaptic and neuronal functions [29–31, 50], was affected by examining the proteomic results for changes in GSK3β-Ser9 phosphorylation and by immunoblot analysis using P21 and P60 Dyrk1a-HT brain lysates. This was also combined with the analysis of potential GSK3β-upstream kinases using the method previously reported [51, 52].

Phosphorylation of GSK3β at Ser9 showed no significant changes, as indicated by the total and PTM proteomic analyses in P21 and P60 Dyrk1a-HT mice (Supplementary Figs. 6, 7, 9). Immunoblot analyses also indicated total and GSK3β-Ser9 phosphorylation levels were comparable between P21 WT and Dyrk1a-KI mice (Supplementary Figs. 11a and 12). Accordingly, potential GSK3β-upstream kinases, including calcium/calmodulin-dependent kinase (CaMK) group/family kinases (i.e., CAMK1–4 and CAMKK), determined as previously reported [51, 52], did not show altered phosphorylations in the PTM-DEPPs from P21 and P60 Dyrk1a-HT mice (Supplementary Fig. 11b). These results suggest that GSK3β is less likely to be important for Dyrk1-HT phenotypes.

Using the same method [51, 52], we sought to identify potential upstream kinases responsible for the downregulated and lithium-responsive PTM-DEPPs in P21 wild-type and Dyrk1a-KI mice. Downregulated PTM-DEPPs were primarily analyzed as they are more likely to represent direct potential substrates of the upstream kinases. Among the candidate upstream kinases for the top 31 downregulated P21 PTM-DEPPs, 24% belonged to the CMGC (CDKs, MAPKs, GSKs, CLKs) group kinases, where DYRK1A belongs to CLKs, and 13% to the STE group (STE20/11/7) (Supplementary Fig 11c). For the top 22 downregulated P60 PTM-DEPPs, 14% of upstream kinases belonged to the CMGC group, 12% to the CAMK group, and 9% to the STE group (Supplementary Fig 11d). These results suggest that some of the top downregulated P21/P60 PTM-DEPPs likely represent direct substrates of DYRK1A.

We then investigated the functions of several PTM-DEPPs, as their roles remain uncharacterized. Specifically, we focused on Kalirin and Elavl2, which are known to regulate synaptic function and dendritic arborization [45–49], although Elavl2 showed relatively lower scores for Dyrk1a/GSK3β-dependent phosphorylation compared to Kalirin. Given that the phosphorylation sites of these proteins, Kalirin-S488 and Elavl2-S221, are not well characterized, we generated phospho-mimic (Kalirin-7-S488D and Elavl2-S221D) and non-phosphorylatable mutants (Kalirin-7-S488A and Elavl2-S221A). These mutants were then tested for their functions in cultured wild-type (WT) and Dyrk1a-HT knock-in (KI) hippocampal neurons. Kalirin-7-S488D (phospho-mimic) overexpressed in cultured neurons enhanced the dendritic arborization of both WT and Dyrk1a-HT neurons, as measured by Sholl analysis and compared to its non-phosphorylatable form (Kalirin-7 S488A) (Supplementary Fig. 11e, f). Likewise, Elavl2-S221D increased dendritic arbors of WT and Dyrk1a-HT neurons, as compared to Elavl2-S221A (Supplementary Fig. 11e, f). These results suggest that Kalirin-S448 and Elavl2-S221 phosphorylations may regulate dendritic arborizations in hippocampal neurons and are consistent with the suppressed dendritic arborization in Dyrk1a-HT mice (Fig. 3b).

## Discussion

Here, we generated Dryk1a-KI mice carrying a patient-derived Dyrk1a-KI mutation (I48 K), characterized their phenotypes at proteomic, synaptic, neuronal, brain, and behavioral levels, and demonstrated that early postnatal chronic lithium treatment can prevent these phenotypes from appearing at juvenile and adult stages.

A key phenotype of our Dyrk1a-KI mice is severe microcephaly accompanied by brain volume decreases of 5–25% across different regions (Fig. 1). These region-specific decreases may reflect varying levels of Dyrk1a expression or interactions with other mechanisms. While further investigation is needed, the significant and widespread brain volume reductions are consistent with the microcephaly observed in the human individual carrying the I48K mutation [6, 10]. One possible mechanism underlying this phenotype could be the signaling pathway alterations reflected in the P21 PTM-DEPPs, which suggested changes in insulin, cAMP, oxytocin, AMPK, gap junction, longevity, ErbB, thyroid hormone, autophagy, and growth hormone signaling pathways (Supplementary Fig. 7c). Consistent with this possibility, early lithium treatment of Dyrk1a-KI mice reverses most (8 out of 10) of these pathways (insulin, cAMP, oxytocin, AMPK, gap junction, longevity, ErbB, and autophagy) (Fig. 5b) and rescues the brain-volume phenotypes at ~P21 (Fig. 4a). Interestingly, the rescued pathways have been implicated in regulating brain development and function, as well as in various brain disorders [53–60]. A previous study using *Dyrk1a* deleted in Emx1-positive excitatory neurons implicated TrkB-BDNF, ERK/MAPK, and mTOR signaling pathways in the pathophysiology underlying the limited growth of cortical neurons [20]. Another previous study has shown that two patient-derived *Dyrk1a*-truncating mutations (R205X and E239X) introduced by in utero electroporation can cause deficits in neuronal and synaptic development [8]. Our study extends these findings by demonstrating that a patient derived mutation in *Dyrk1a*, which is expressed in various brain cell types, can cause lithium-responsive deficits in multiple signaling pathways in a spatiotemporal context better mimicking the patient condition.

Our study suggests that a selective decrease in excitatory synaptic density (Fig. 2d–g) may be a mechanism underlying the Dyrk1a-KI mouse phenotypes. In support of this possibility, early lithium treatment of Dyrk1a-KI mice rescues their synaptic, dendritic, brain size, and behavioral phenotypes (Figs. 3 and 4). A possible mechanism leading to the decrease in excitatory synapse density could be suppression of dendritic arborization (Fig. 3b, c), which may involve multiple signaling pathways enriched in the lithium-rescued ~P21 PTM-DEPPs (Fig. 5b). Dendritic arborization is regulated by multiple extracellular signals (i.e., neurotransmitters and neurotrophins) [61] and signaling pathways, including the insulin [62–64], AMPK [65–67], gap junction [68–70], and ErbB [61, 71] pathways.

Alternatively, the decreased excitatory synaptic density seen in Dyrk1a-KI mice could involve synaptic protein deficits and/or dendritic shrinkage. The ~P21 lithium-rescued PTM-DEPPs are enriched for synapse-related GO functions and SynGO proteins (Fig. 5b; Supplementary Fig. 6b). A notable protein among the lithium-rescued synaptic proteins is Kalirin, a Rho-GEF that regulates synaptic function as well as dendritic arborization and are implicated in neurodevelopmental disorders (i.e., schizophrenia) [48, 49]. Another is Elavl2, a member of the ELAVL family of RNA-binding proteins, which regulate posttranscriptional processes, such as neuronal mRNA stability and splicing [72, 73], and are implicated in neural development and brain disorders [74]. Elavl2 is the least studied member of the family, but it is known to localize to and regulate synapses [45, 46], and been implicated in neurodevelopment, synaptic regulation, and ASD [75]. Our findings in the present study, particularly the phosphorylation-dependent regulation of dendritic arbors via Kalrn-S448 and Elavl2-S221 (Supplementary Fig. 11e, f), suggest that Dyrk1a-dependent/related phosphorylation may regulate Kalrn- and Elavl2-mediated dendritic growth and synaptic functions. This parallels the NMDA receptor activation-dependent phosphorylation of CaMKII and Kalirin-7 (at Thr95), which enhances Kalirin-7’s Rho-GEF activity [76] and hippocampal LTP [77].

Interestingly, early lithium treatment in Dyrk1a-KI mice is associated with somewhat different molecular effects in the short and long terms ( ~ P21 and ~P60, respectively) (Fig. 5; Supplementary Figs. 5–10). At ~P21, the early lithium treatment is seen to rescue multiple signaling pathways to normalize dendritic arborization and excitatory synaptic density. This signaling pathway-focus rescue effect is less pronounced at ~P60, when the effect appears to focus on synaptic proteins. It is possible that the rescue of signaling pathways at ~P21 may prevent the changes in synaptic proteins from appearing at ~P60. This is in line with the notion that early correction of key pathologies is important for long-lasting prevention of ASD-related phenotypes at later stages [37].

Lithium treatment is known to have multiple action mechanisms, including GSK3β inhibition. GSK3β exerts pleiotropic effects on neural development through the regulation of multiple signaling pathways, including the PI3K-Akt-mTOR pathway [29, 30] and β-catenin-dependent gene expression [78]. In addition, GSK3β regulates excitatory synapses and dendritic spines through multiple mechanisms, including synaptic plasticity (i.e., LTD promotion and LTP suppression) [31] and β-catenin degradation [79]. Lithium also regulates neuronal and synaptic development and functions through various signaling pathways, including GSK3β and CREB [80–84]. For example, lithium promotes the synaptic localization of the GluA2 AMPA receptor through δ-catenin [32]. In addition, Dyrk1a acts as a priming kinase for GSK3β in the phosphorylation of eIF2Bε and tau [85]. However, our analysis of GSK3β phosphorylation and its potential upstream kinases suggests that GSK3β inhibition may not be critical in the lithium-dependent rescue of Dyrk1a-KI phenotypes, and, instead, the data points to the importance of direct Dyrk1a substrates (Supplementary Fig. 11a–d).

Lithium, which is a gold-standard medication for bipolar disorder, was recently shown to be useful for neurological disorders [33–36]. Our results, together with the recently reported beneficial effects of lithium in a Shank3-mutant mouse model of ASD [86] and fragile X mouse models [87], suggest that lithium could have therapeutic potential for ASD and related neurodevelopmental disorders.

## Supplementary information

Supplementary Information

Supplementary Table 1

Supplementary Table 2

Supplementary Table 3

Supplementary Table 4

Supplementary Table 5

Supplementary Table 6

Supplementary Table 7

## Data Availability

i) The RNA-Seq data for Dyrk1a-KI and WT mice at P21 and P60 have been deposited at the GEO (Gene Expression Omnibus) repository with accession GSE274730. ii) The raw MS data files for total and PTM proteomes from heterozygous Dyrk1a-KI and WT mice at P21 have been deposited to the ProteomeXchange Consortium via the PRIDE partner repository with the identifier PXD053587 for total proteome analysis and the identifier PXD050102 for phosphoproteome analysis. iii) The raw MS data files for total and PTM proteomes from heterozygous Dyrk1a-KI and WT mice at P60 have been deposited to the repository MassIVE with the identifier PXD049356 for total proteome analysis and the identifier PXD049357 for phosphoproteome analysis. iv) The PTM data for P60 from homozygous Dyrk1a-KI and WT mice have been deposited to the ProteomeXchange database under the accession number of PXD050100.
