# Supplementary material for: Lithium normalizes ASD-related neuronal, synaptic, and behavioral phenotypes in DYRK1A-knockin mice
Source: Mol Psychiatry. 2024 Dec 5;30(6):2584–96. doi: 10.1038/s41380-024-02865-2 (PMC12092274; doi:10.1038/s41380-024-02865-2)
Supplement: Supplementary file 1 — Supplementary Information [file 41380_2024_2865_MOESM1_ESM.pdf]

Supplementary Information

Lithium normalizes ASD-related neuronal, synaptic, and behavioral phenotypes in DYRK1A-knockin mice

Junyeop Daniel Roh[1,*], Mihyun Bae[1,*], Hyosang Kim[1], Yeji Yang[2,3], Yeunkeum Lee[1,4], Yisul Cho[5], Suho Lee[1], Yan Li[1], Esther Yang[6], Hyunjee Jang[7], Hyeonji Kim[7], Hyun Kim[6], Hyojin Kang[8], Jacob Ellegood[9,10], Jason P. Lerch[9,11], Yong Chul Bae[5], Jin Young Kim[3], and Eunjoon Kim[1,2,#]

[1]Center for Synaptic Brain Dysfunctions, Institute for Basic Science (IBS), Daejeon 34141, Korea; [2]Department of Biological Sciences, Korea Advanced Institute for Science and Technology (KAIST), Daejeon 34141, Korea; [3] Digital Omics Research Center, Korea Basic Science Institute, Ochang 28119, Korea; [4]Korea Institute of Drug Safety & Risk Management, Anyang 14051, Korea; [5]Department of Anatomy and Neurobiology, School of Dentistry, Kyungpook National University, Daegu 41940, Korea; [6]Department of Anatomy and BK21 Graduate Program, Biomedical Sciences, College of Medicine, Korea University, Seoul 02841 Korea; [7]Bertis Inc., Gwacheon 13840, Korea; [8]Division of National Supercomputing, KISTI, Daejeon 34141, Korea; [9]Mouse Imaging Centre, Hospital for Sick Children, Toronto, Ontario, M5T 3H7, Canada; [10]Bloorview Research Institute, Holland Bloorview Kids Rehabilitation Hospital, Toronto, Ontario, M4G 1R8, Canada; [11]Wellcome Centre for Integrative Neuroimaging, University of Oxford, Oxford, Oxfordshire, OX39DU, UK;
*These authors contributed equally to the work; #Corresponding author.

Supplementary materials and methods

Electrophysiology

Mice (P17–23) were anesthetized using isofluorane (Terrell), and brains were surgically prepared after carefully removing the skull. Sagittal hippocampal slices (300 µm) were made using a vibratome (Leica VT1200) in ice-cold sucrose-based artificial cerebrospinal fluid (sCSF) buffer containing (in mM): 212 sucrose, 10 d-glucose, 25 $NaHCO_3$, 5 KCl, 1.25 NaH_2PO_4, 1.25 l-ascorbic acid, 2 Na-pyruvate, 3.5 $MgSO_4$, and 0.5 $CaCl_2$ bubbled with 95% O_2 and 5% CO_2. The slices were recovered in a chamber while submerged in artificial cerebrospinal fluid buffer (aCSF) held at 32°C, containing (in mM): 125 NaCl, 10 d-glucose, 25 $NaHCO_3$, 2.5 KCl, 1.25 NaH_2PO_4, 1.3 $MgCl_2$, and 2.5 $CaCl_2$ for 30 min and subsequently recovered at room temperature for 30 min while being bubbled with 95% O_2 and 5% CO_2 through the entirety of the recovery process and recordings.

For whole-cell patch recording, borosilicate glass pipettes (Harvard Apparatus) were pulled with a micropipette puller (Narishige). To record CA1 pyramidal cells, recording pipettes (3–4 MΩ) were filled with the following intracellular solutions: (i) for EPSC experiments (in mM): 117 $CsMeSO_4$, 10 TEA-Cl, 8 NaCl, 10 HEPES, 5 QX-314-Cl, 4 Mg-ATP, 0.3 Na-GTP, 10 EGTA with pH 7.3, and 285–300 mOsm, and (ii) for IPSC experiments (in mM): 115 CsCl2, 10 TEA-Cl, 8 NaCl, 10 HEPES, 5 QX-314-Cl, 4 Mg-ATP, 0.3 Na-GTP, 10 EGTA with pH 7.3, and 285–300 mOsm.

Data were filtered at 2 kHz and digitized at 10 kHZ using Multiclamp 700B and 1440 Digitizer (Molecular Devices). Series resistance was monitored in each sweep by measuring the peak amplitude of capacitance currents in response to short hyperpolarizing step pulse (5 mV, 40 ms). The acquired data were analyzed using Clampfit 10 (Molecular Devices).

Brain cells at P17–23 were used to measure miniature currents while being held at −70 mV in whole-cell configuration. For mEPSC measurements, picrotoxin (100 μM) and tetrodotoxin (10 μM) were added to the aCSF to block action potentials and inhibitory currents, respectively. For mIPSC measurements, NBQX (100 μM), AP5 (100 μM), and tetrodotoxin (TTX; 10 μM) were added to block AMPAR-mediated currents, NMDA-mediated currents, and action potentials, respectively. For spontaneous miniature recordings—sEPSC and sIPSC—similar process as mEPSC and mIPSC were followed, except for TTX, which was not added to allow for network activity and modulation of synaptic transmission.

For NMDA/AMPA ratio, picrotoxin (100 μM) was added to block $GABA_A$ receptor-mediated currents from slices at P17–21. CA1 pyramidal cells were whole cell patched and voltage clamped at −70 mV. To measure AMPAR-mediated EPSCs stratum radiatum (SR) dendritic field was stimulated every 15s by stimulating pipette filled with aCSF solution. After obtaining a stable baseline, 30 consecutive responses were recorded as AMPAR components. The holding potential was then changed to +40 mV on the same neuron to measure NMDAR-mediated EPSCs. The NMDA component was determined by measuring the amplitude 60 ms after the

stimulation. The ratio was calculated by dividing the average of NMDAR EPSCs (peak amplitudes) by the average of AMPAR EPSCs.

For extracellular field recordings, both stimulating and recording pipettes were filled with the aCSF solution and recorded at the stratum radiatum (SR) of the hippocampal CA1 region by stimulating the axon fibers of Schaeffer collateral from CA3. To induce HFS-LTP, high-frequency stimulation (100 Hz, 1 s) was applied after a stable baseline of 20 minutes. For TBS-LTP, after acquisition of a stable baseline, the slices (4–6 weeks) were stimulated with 10 trains of four pulses (theta bursts) at 100 Hz and responses were recorded for 1 hour after the stimulation. For NMDA-dependent LTD, we added picrotoxin (100 μM) to aCSF and used P16–22 slices. After a stable baseline was reached for 20 min, we stimulated the slices with low-frequency stimulation (1 Hz, 900 pulses for 15 min) followed by 1-h measurements of responses. For mGluR-dependent LTD, after 20-min stable baseline, we bath applied DHPG (50 μM) in aCSF to induce LTD for 10 min and recorded the responses for 1 hour in the presence of picrotoxin (100 μM). The average rise slopes of fEPSPs during the last 10 min were compared for both LTP and LTD.

For input–output experiments, input was defined as the peak amplitude of the fiber volley, and the output was defined as the initial slope of fEPSP. The stimulation intensity ranged from 5 to 35 μA with 2.5-μA increments per minute. Paired-pulse facilitation was measured as by evoking two fEPSPs with inter-stimulus intervals ranging from 25 to 300 ms, and the ratios were calculated by dividing the initial slope of the second fEPSP by that of the first fEPSP.

Sholl analysis

Biocytin (0.3%) mixed into intracellular solution was injected into CA1 pyramidal cells and layer 2 prefrontal neurons of 3 weeks-old mice following either vehicle or lithium treatment. After biocytin injection for 10 minutes, the glass capillary was detached from the cell membrane slowly until the giga-seal was once again observed followed by return of initial pipette resistance (3–4 MΩ). The injected slices were fixed in 4% PFA overnight, then incubated in 3% donkey serum, 0.3% Triton X-100, and Streptavidin, Alexa FluorTM 488 Conjugate (ThermoFisher, S11223, 1:500) in PBS for 24 h at 4 ℃.

Sholl analysis of hippocampal cultured neurons

Primary cultures of mouse neurons were prepared from embryonic day 17 (E17) male Dyrk1A knock-in or WT embryos. Dissected hippocampal tissues were maintained in plain Neurobasal-A medium (Thermo Fisher Scientific) for 1~3 days during which, genotyping was performed. Tissues were dissociated by enzyme digestion with papain (Worthington Chemical, LS003127) and subsequently transfected with mutant constructs: Kalirin-7 (pEAK10-His-Myc-Kal7 (Addgene, #25454) S488A, Kalirin-7 S488DE, Elavl2 (Origene, MG205762) S221A, and Elavl2 S221D. The plasmids were co-transfected with pAAV-hSyn-mCherry (Addgene, #114472) for visualizing neurons overexpressing each construct.

For Kalirin-7 mutations, pEAK10-His-Myc-Kal7 was cut by NcoI and PciI, and PCR was performed using the following primers: cctggatgtcctgcagcgtcccctggaccctgggaactccgagtccctcacagcc (Forward) and

ggctgtgagggactcggagttcccagggtccaggggacgctgcaggacatccagg (Reverse) for the S488D mutation and ggctgtgagggactcggagttcccaggggccaggggacgctgcaggacatccagg (Forward) and cctggatgtcctgcagcgtcccctggcccctgggaactccgagtccctcacagcc (Reverse) for the S488A mutation. Finally, the PCR products were ligated with the original vector cut by NcoI and PciI.

For Elavl2 mutations, PCR using Pfu (SPX16-R250 Solgent) was performed using the following primers: cagctgtaccaggctccaaacagaagg (Forward) and ccttctgtttggagcctggtacagctg (Reverse) for the S221A mutation and cagctgtaccaggatccaaacagaagg (Forward) and ccttctgtttggatcctggtacagctg (Reverse) for the S221D mutation, and the original plasmid was digested with Dpn1 (Enzynomics). All mutant constructs were double checked with DNA sequencing. Transfection was carried out using the Mouse Neuron Nuclofector Kit (Lonza, VPG-1001) according to the manufacturer’s protocol.

Following transfection, neurons were plated on poly-D-lysine-coated 18-mm glass coverslips with a plating medium (Neurobasal-A medium supplemented with 2% B-27, 10% FBS, 1% GlutaMax, and 1 mM sodium pyruvate (all from Thermo Fisher Scientific) at a density of 1 x 10^5 cells per coverslip. After 4 hours, the plating medium was replaced with FBS-free culture medium (Neurobasal-A medium supplemented with 2% B-27, 1% GlutaMax, and 1 mM sodium pyruvate). The medium was refreshed every 7 days with a 50% replacement. At the proper time point, neurons were fixed with 4% PFA and stained with anti-mcherry (ab205402, abcam) antibody for better signals. Images were captured by confocal microscopy (Carl Zeiss, LSM780). Dendritic arbors were traced by neuTube 1.0 software and the number of intersections per 25 μM interval from soma was analyzed by ImageJ.

Brain size measurement

Brains of 3 weeks-old or 8 weeks-old Dyrk1a KI mice and WT counterparts were collected and imaged with a scale in a top-down view. Calibrating using the scale within the picture, the brain size was analyzed using ImageJ.

Brain lysates and western blot

Mouse brains of respective ages were extracted on ice and homogenized in ice-cold homogenization buffer (0.32 M sucrose, 10 mM HEPES, pH 7.4, 2 mM EDTA, pH 8.0, 2 mM EGTA,601pH8.0, protease inhibitors, phosphatase inhibitors). Total lysates were prepared by boiling brain tissues with β-mercaptoethanol directly after homogenization. Immunoblot conditions: Dyrk1a (Abnova H00001859-M01 and Abcam ab156818), GSK3β (Cell Signaling 12456), p-GSK3β (S9, Cell Signaling 9336), PSD-95 (home-made #1689), β-actin (Sigma A5316) and α-tubulin (Sigma T5168) at 4°C overnight. Fluorescent secondary antibody signals were detected using Odyssey Fc Dual-Mode Imaging System.

In situ hybridization

In situ hybridization was performed as previously described [1]. Mouse brain sections (14 μm thick) at embryonic day (E18) and postnatal days (P0, P7, P14, P21 and P56) were prepared using a cryostat (Leica CM 1950). A hybridization probe for mouse *Dyrk1A* mRNAs was prepared using pGEM-7Zf containing nucleotides 147-446 (300 bp), 5’-GAG AGG GGA TCC ATG CAT ACA GGA GGA GAG AC-3’ (forward) and 5’-GAG CTC GAA TTC CAA GTC CAC AGA GAG TTT TC-3’

(reverse) of *Dyrk1a* mRNA (NM_001113389.1), Riboprobe System (Promega), and α-[^{35}S] UTP.

Immunohistochemistry

Adult WT and KI mouse (2-3 months) were transcardially perfused with a heparin solution and 4% formaldehyde (sigma 252549), post-fixed for 24 h, and coronal sections were generated (40 μm) using a vibratome (Leica, VT1200s). These sections were permeabilized using TBST/NDS (1x TBS, 0.2% Triton X-100, and 5% NDS (normal donkey serum) for 2 h at room temperature. Permeabilized sections were stained with primary antibodies in TBST/NDS overnight at 4°C, followed by secondary antibody staining and mounting using VECTASHIELD® Antifade Mounting Medium with DAPI (Vector, H-1200). To check the gross morphology of the brain, NeuN antibody (Millipore ABN90, 1:1000) was used. Glial cells were stained using antibodies for S100β (Abcam, ab52642). Neural filaments also visualizing with Antibody for Neurofilament M (NF-M) (BioLegend 841001). Z-stacked images were acquired using a confocal microscope (Zeiss, LSM780).

Transcriptomic analysis

Dyrk1a-KI and WT mice at P21 and P60 were used for each group (n=4-6). The extracted mouse brains were preserved in RNAlater solution (Ambion) and stored at -20 °C. Poly-T oligo-attached magnetic beads were utilized to purify poly-A mRNAs. RNA concentrations were quantified using Quant-IT RiboGreen (Invitrogen, R11490), and RNA integrity was determined using TapeStation RNA screen tape (Agilent Technologies), after which only high-quality RNAs (RIN > 7.0) were selected for

cDNA library construction using TruSeq Stranded mRNA Sample Prep Kit(Illumina). Indexed libraries were submitted to an Illumina HiSeq 4000(Illumina), and paired-end (2 x 100 bp) sequencing was performed by Theragen Bio. Transcript abundance was estimated with Salmon (v1.1.0) [2] in Quasi-mapping-based mode onto the Mus musculus genome (GRCm38) with GC bias correction (-gcBias). The acquired abundance data was imported to R (v.4.1.2) with tximport [3] package and differential gene expression analysis was performed using R/Bioconductor DEseq2 (v1.34.0) [4]. Normalized read counts were computed by dividing the raw read counts by size factors and fitted to a negative binomial distribution. The p-values were adjusted for multiple testing with the Benjamini-Hochberg correction. Genes with an adjusted p-value of less than 0.05 were considered as differentially expressed. Gene Set Enrichment Analysis (GSEA) [5, 6] was performed to determine whether a priori-defined gene sets would show statistically significant differences in expression between Dyrk1a-KI and WT mice. Enrichment Analysis was performed using GSEA Linux(v4.2.3) GSEAPreranked module on gene set collections downloaded from Molecular Signature Database (MSigDB, v7.5.1). GSEAPreranked was applied using the list of all genes expressed, ranked by the fold change and multiplied by the inverse of the p-value with recommended default settings (1,000 permutations and a classic scoring scheme). The false discovery rate (FDR) was estimated to control the false positive finding of a given normalized enrichment score (NES) by comparing the tails of the observed and null distributions derived from 1,000 gene set permutations. The gene sets with an FDR of less than 0.05 were considered as significantly enriched. Integration and visualization of the GSEA results were performed using the EnrichmentMap Cytoscape App (version 3.8.1) [7, 8].

Proteomic analysis

Three separate PTM scans (and a total proteomic analysis) by LC-MS/MS were performed using i) P21 HT/heterozygous-Dyrk1a-KI mice, ii) P60 HT-Dyrk1a-KI mice, and iii) P60-HM/homozygous-Dyrk1a KI mice and their WT counterparts.

i) For P21 HT-Dyrk1a-KI mice, peptide preparation and proteomic analysis were performed by Bertis (Korea). Whole brains from vehicle or lithium treated male heterozygous Dyrk1a-KI and WT mice (4 biological replicates) were homogenized at 4°C using hand-held homogenizer in lysis buffer containing 8 M urea in 100 mM ammonium bicarbonate and phosphatase inhibitor cocktail. The lysates were centrifuged and further sonicated, and were subjected to protein reduction in 10 mM DTT (dithiothreitol), and subsequent alkylation in 25 mM IAA (iodoacetamide). The alkalized samples were treated with 0.4 μg/μL Promega trypsin (1:25 enzyme : protein), then desalted and reconstituted. These samples were then labeled with 16-plex TMT isotope [9] and desalted to remove any remaining TMT reagent without binding to the sample. Phosphorylated peptides were then concentrated using IMAC magnetic beads (Cell signaling) according to the manufacturer's instructions. The samples were fractionated into 12 fractions by high pH fractionation kit (Thermo scientific), dried using SpeedVac, reconstituted in 0.1% TFA before MS analysis.

Nano-LC Ultimate 3000 system coupled to Thermo Orbitrap Exploris 480 was used for LC-MS/MS analysis. The LC-DDA-MS/MS data were processed using the Comet [10] search engine with the following settings: database consisting of UniProt mouse reference database (released on April 2019); precursor and fragment mass tolerance of 20 ppm; semi-tryptic peptide search; up to two missed cleavages, and

peptide length of 7 to 50 were allowed; and carbamidomethylation of cysteine was set as a fixed modification, and oxidation of methionine was set as a variable modification. Then, we used the FragPipe (version 18) with PTMProphet [11] for localization of modifications. The identification results were filtered using Percolator [12] and ProteinProphet4 including 1% FDR at the PSM, peptide, and protein level.

ii) For P60 HT-Dyrk1A-KI mice, peptide preparation and phosphopeptide enrichment were performed using whole brains from heterozygous Dyrk1a-HT and WT mice treated/untreated with lithium (4 replicates). Mouse brains were lysed with 1x sodium dodecyl sulphate (SDS) buffer containing 5% SDS and 50 mM triethylamonium bicarbonate (TEAB) at pH 8.5. The lysates were digested with S-trap method following the provided method. Tryptic digested peptides were labeled with 18-plex TMT isotopes (Thermo Fisher Scientific). After TMT labeling, all peptides were combined and dried by Speed-Vac. Before LC-MS/MS analysis, samples were desalted with Pierce peptide desalting spin columns (Thermo Fisher Scientific) and fractionated into 20 fractions by basic reverse phase liquid chromatography. 5% of the sample were used for total proteome analysis and then remaining 95% was reserved for phosphoproteome analysis. The remaining 95% of the samples were subjected to fractionation into 10 fractions for phosphopeptide enrichment. Firstly, Ni-NTA magnetic agarose beads were washed three times with DW and then incubated with 100mM EDTA (pH 8.0) for 30 minutes on a rotator. Next, 100mM $FeCl_3$ solution is added, and the mixture is rotated for 30 minutes during incubation. The prepared Fe3+-NTA beads are then washed with DW and subjected to overnight incubation in 80% ACN in 0.1% TFA containing each sample at 4°C on a rotator. In the end, the

phosphopeptides attached to the beads were eluted by exposing to elution buffer (50% ACN in 1% ammonium hydroxide). Following elution, the phosphopeptides were promptly acidified to a pH of 3.5–4.0 using 10% TFA before undergoing vacuum drying.

LC-MS/MS analysis was performed using an UltiMate 3000 RSLCnano system (Thermo Scientific) coupled to a Orbitrap Fusion Lumos mass spectrometer (Thermo Fisher Scientific). The mobile phases A and B were composed of 0 and 95.0% acetonitrile containing 0.1% formic acid, respectively. The LC gradient at a flow rate of 250 nL/min was applied during 120 min for the peptide separation. The Orbitrap Fusion Lumos was operated in data-dependent mode, and the MS2 scans were performed with HCD fragmentation (37.5% collision energy). MS/MS spectra were identified and quantified using Integrated Proteomics Pipeline software with the Uniprot mouse database. Search parameters were precursor mass tolerance of 20 ppm, a fragment ion mass tolerance of 200 ppm, two and more peptides assignments for protein identification at a false positive rate less than 0.01, and TMT reporter ion mass tolerance of 20 ppm. For the phosphopeptide identification, phosphorylation of serine(S), threonine, and tyrosine was set as the differential modifications, with a maximum of three additional modifications permitted. Statistical analyses were conducted using Perseus software (version 1.6.15). The expressions of proteins and phosphopeptides between samples were compared with Welch's t-test with p value set at < 0.05.

iii) For P60 HM-Dyrk1a-KI and their WT counterparts, protide preparation and proteomic analysis were conducted by Cell Signaling. PTMScan Multi-

Pathway results for WT and homozygous/HM Dyrk1a-KI mouse brains (2–3 month) were obtained using the PTMScan Multi-Pathway Enrichment method (PTMScan® Multi-Pathway Enrichment Kit #75676, Cell Signaling Technology). For HM Dyrk1a-KI mice, brains from 1 male and 2 female HM KI mice and corresponding WT with matched age and sex were used. Briefly, mouse brain samples containing the whole brains were dissected on ice and snap-frozen in liquid nitrogen. Then brain samples were trypsin-digested and fractionated by solid-phase extraction. Digested phosphorylated peptides were enriched by site-specific antibodies conjugated to protein A beads for peptide immunoaffinity purification (PTMScan® Multi-Pathway Enrichment Kit) and analyzed by LC-MS/MS (cell signaling)

DAVID Gene Ontology (GO) and SynGo analyses of the three proteomic results (i–iii) for the annotation of synaptic proteins and functions were conducted using significantly changed terms ($p < 0.05$, $|FC| > 1.2$) (http://david.ncifcrf.gov).

Behavioral tests

Ultrasonic vocalization (USV) test

Each subject mouse's home cage was placed into the USV chamber. The microphone was placed about 20 cm above the testing arena. Age-matched female mice (C57BL/6J) were randomly introduced to each subject male mice's cage. Subject male mice and intruder female mice freely interacted for 5 min, during which USVs were recorded. For the pup USV test, each pup (postnatal day 3, 5, 7, and 9) was isolated from the dam. An ultrasound microphone (Avisoft) was used to record USVs. USVs were recorded in the USV chamber for 3 min. Avisoft SASLab Pro

software was used to analyze USVs. Spectrograms were generated with 256 Fourier transformation length, 75% overlap of temporal resolution, and 25 kHz of lower cut-off frequency. Call duration and number of the spectrum were measured.

Laboras test

Long-term (96 h) locomotor, climbing, rearing, grooming, eating, and drinking activities were recorded and automatically analyzed using the Laboratory Animal Behavior Observation Registration and Analysis System (LABORAS: Metris). Mice were individually caged in a specialized LABORAS recording environment for the duration of recording and fed *ad libitum*.

Juvenile play

Direct interaction was performed as previously described [13]. Social interaction test sessions were conducted during the first half of the dark cycle in a quiet, dimly lit room illuminated by a single 25 W red light. For juvenile play, P21 mice were brought to the testing room from their home cages for pre-exposure to the experimental conditions. After an hour of isolation, pairs of same sex and genotype but non-sibling mice from different litters were placed in the testing arena, and their interactions were recorded for 15 min. Nose-to-nose sniffing, following, mounting, and allo-grooming were quantified manually as measures of direct social interaction.

Juvenile repetitive behaviors

For homecage self-grooming test, each mouse was placed into a new home cage without bedding and allowed to freely move. Self-grooming activity during 15 min

was analyzed. The light in the booth was adjusted to 50 lux. Self-grooming was defined as stroking or scratching of its face or body, or licking its body parts. For digging test, home cages were filled with 2-cm-deep beddings, where mice were placed for 5 min, and the activities were measured and analyzed. Digging was defined as digging out beddings using its head or forelimbs. Self-grooming and digging were scored as the duration of each behavior in a double-blind manner.

Maternal homing test

Maternal homing test for juvenile mice was performed as previously described [14] P19 WT and Dyrk1a-KI mice were separated from the mother for at least 30 min before testing. The testing was divided into two stages; (i) nest homing and (ii) maternal homing. For the nest homing, fresh bedding was placed in one corner and bedding from home cage was placed in the opposite corner of a 40 × 40 × 40 cm white acryl box, with the other two corners being empty. The subject was placed in one empty corner, and its movements were recorded for 3 min. For the maternal homing stage, an empty container and another container containing the mother of the subject were placed in the two previously empty corners. The subject mouse was then placed in the corner with the home cage bedding, and its movements were recorded for 5 min.

Rotarod test

Mice were placed on the Rota-rod apparatus (Ugo Basile). Starting from initial speed of 4 rpm, the rotarod reached maximum speed of 40 rpm during a 5-min test period. Illumination intensity was set to 40 lux, and the test was performed for 5 days.

Latency to falling off the rod was measured manually for each mouse.

Open-field test

Each mouse was placed in the customized open field box (40 cm × 40 cm × 40 cm) in 100 lux setting. The mouse was allowed to move freely inside the box (1 hour for adult and 20 min for juvenile), and the activity was video-recorded. Parameters of each mouse activity such as distance travelled and time spent in the center region of the box were analyzed using EthoVision XT 10 (Noldus).

Three-chamber test

The three-chamber apparatus as previously described [15, 16] is used. Mice were isolated in a single cage for 3 days prior to the test, while the stranger mice (129S1/SvlmJ strain) were group-housed (4–6 mice). The test consisted of two phases: empty-empty (habituation), and stranger 1-object (S1-O). The test was conducted after 30-min habituation in an experimental booth. The white acrylic three-chambered apparatus (40 cm width × 20 cm height × 26 cm depth with a 12-cm-wide center chamber and 14-cm-wide side chambers) included two small containers for an object or a stranger mouse in the upper or lower corner of the two side chambers. In the first habituation phase, a test mouse was placed in the center area of the three-chambered apparatus and allowed to freely explore the environment for 10 min. In the second S1-O phase, a stranger mouse (S1) and an inanimate blue cylindrical object (O) were placed in the two corner containers. A stranger mouse was randomly positioned in the left or right chamber. The test mouse was allowed to explore the stranger mouse or the object freely. For the analysis, sniffing times were measured

using EthoVision XT 10 (Noldus) software. Sniffing was defined as the nose part of the test mouse being positioned within 20% from a container.

Elevated plus-maze and light-dark tests

For the elevated plus-maze test, each mouse was placed on the center of the elevated plus maze (EPM) and allowed to explore for 10 min. The cross-shaped apparatus was made of gray acrylic plates and was elevated 50 cm from the floor, with two open arms (30 × 5 × 0.5 cm, 300 lux) and two closed arms (30 × 5 × 30 cm, 30 lux). Time spent in open or closed arms and the frequency of entries to each arm were automatically measured using EthoVision XT 10 (Noldus). For the light-dark test (LD), mice were placed in the light chamber with their heads toward the opposite wall from the dark chamber and allowed to explore the light-dark apparatus (20 × 13 × 20 cm, 300 lux for light chamber, 20 × 13 × 20 cm, 0 lux for dark chamber), which has a 5-cm wide entrance between the two chambers. The latency to enter the dark chamber and time spent in light and dark chambers were analyzed using EthoVision XT 10 (Noldus).

Fear-conditioning test

For context fear conditioning test, a day before conditioning day, subject mice were placed in the fear chamber and habituated into the chamber for 5 min. On conditioning day, mice were introduced again to the fear chamber and allowed to freely explore the environment for 2 min, and then received five-foot shocks (0.8 mA, 1-sec interval, 120-sec intervals). After the last shock, the mice were left in the box for additional 2 min, making the total experimental time 12 min. After 24 h, the mice

were placed in the same conditioning box and allowed to freely explore for 10 min without any stimuli, and the freezing levels of the mice were quantified (for fear conditioning test version A). This measurement of freezing levels were repeated 7 days after the last shock (for fear conditioning test A). For fear conditioning version B, mice were reintroduced to the conditioning box only for 7 days after the last shock without the 24 hour measurement. All freezing behaviors were recorded and analyzed using FreezeFrame software (Coulbourn Instruments). For cued fear conditioning, a mouse was introduced to the same fear box and then given 3 min to explore the environment, followed by three foot shocks (0.8 mA, 1 s, unconditioned stimulus (US)) at the end of sound (75 dB, 8 kHz tone, 20 s, conditioned stimulus (CS)) with 1 min intervals for 3 min (this process totally take 6 min). On the day after, the mice that already cued fear-conditioned were introduced to a different fear box (context B) and allowed to move for 3 min without tone (CS−) and 3 min with tone (CS+; 75 dB, 8 kHz tone, 3 min). Freezing behaviors were analyzed using FreezeFrame 3 (Coulbourn Instrument).

PTZ-Induced seizure

After intraperitoneal injection of pentylenetetrazole (PTZ; Sigma; 40 mg/kg), subject mice were placed in a clean new home cage. Video recordings for 30 min were used to analyze seizure stages defined as follows; stage 1, behavioral arrest; stage 2, myoclonic (jerk) seizures; stage 3: general tonic-clonic seizures. The seizure susceptibility score was defined as follows; 0.2 × 1/(latency to stage 1) + 0.3 × 1/(latency to stage 2) + 0.5 × 1/(latency to stage 3).

Morris water maze test

A hidden platform (10 cm diameter) was placed in a white plastic tank (120 cm diameter). Mice were trained to find the hidden platform 3 trials per day with an inter-trial interval of 30 minutes. The learning phase was performed on consecutive days until the latency to the platform is less than 20 seconds. The day next, we conducted a probe test (1 minute) without the hidden platform. On the next day, we conducted reversal learning after changing the position of the platform to the opposite side. If latency to the platform is less than 20 seconds, the day next, we conducted a probe test (1 minute) without a hidden platform. The recorded video was analyzed using Ethovision XT10 software (Nodulus). Time spent in quadrants and the number of platform passing were calculated

Electron microscopy

WT, vehicle treated Dyrk1a-KI, and lithium treated Dyrk1a-KI mice were deeply anesthetized with a mixture of ketamine (120 mg/kg) and xylazine (10 mg/kg) and were intracardially perfused with 10 ml of heparinized normal saline, followed by 50 ml of a freshly prepared fixative of 2.5% glutaraldehyde and 1% paraformaldehyde in 0.1 M phosphate buffer (PB, pH 7.4). Hippocampus was removed from the whole brain, postfixed in the same fixative for 2 hours and stored in PB overnight at 4 C. Sections were cut transversely on a Vibratome at 70 μm. The sections were osmicated with 0.5% osmium tetroxide (in PB) for 1 hour, dehydrated in graded alcohols, flat embedded in Durcupan ACM (Fluka), and cured for 48 hrs at 60°C. Small pieces containing stratum radiatum of hippocampal CA1 region were cut out of

the wafers and glued onto the plastic block by cyanoacrylate. Ultrathin sections were cut and mounted on Formvar-coated single slot grids. For the analysis of excitatory synapses, sections were stained with uranyl acetate and lead citrate, and examined with an electron microscope (Hitachi H-7500; Hitachi) at 80 kV accelerating voltage. For the analysis of inhibitory synapses, sections were further immunogold stained for GABA.

Postembedding immunogold staining for GABA

Sections were immunostained for GABA by the postembedding immunogold method, as previously described [17], with some modifications. In brief, the grids were treated for 5 min in 1% periodic acid, to etch the resin, and for 8 min in 9% sodium periodate, to remove the osmium tetroxide, then washed in distilled water, transferred to Tris-buffered saline containing 0.1% Triton X-100 (TBST; pH 7.4) for 10 min, and incubated in 2% human serum albumin (HSA) in TBST for 10 min. The grids were then incubated with rabbit antiserum against GABA (GABA 990, 1:10,000) in TBST containing 2% HSA for 2 hrs at room temperature. The antiserum (a kind gift from professor O. P. Ottersen at the Center for Molecular Biology and Neuroscience, University of Oslo) was raised against GABA conjugated to bovine serum albumin with glutaraldehyde and formaldehyde [18] and characterized by spot testing [19]. To eliminate cross-reactivity, the diluted antiserum was preadsorbed overnight with glutaraldehyde (G)-conjugated glutamate (500 µM, prepared according to a previous study [20]). After extensive rinsing in TBST, grids were incubated for 3 hrs in goat anti-rabbit IgG coupled to 15 nm gold particles (1:25 in TBST containing 0.05%

polyethylene glycol; BioCell). After a rinse in distilled water, the grids were counterstained with uranyl acetate and lead citrate, and examined with an electron microscope (Hitachi H-7500; Hitachi) at 80 kV accelerating voltage. To assess the immunoreacivity for GABA, gold particle density (number of gold particles per μm^2) of each GABA+ terminal was compared with gold particle density of terminals which contain round vesicles and make asymmetric synaptic contact with dendritic spines (background density). Terminals were considered GABA-immunopositive (+) if the gold particle density over the vesicle-containing areas was at least five times higher than background density.

Quantitative analysis of inhibitory synapses

For quantification of excitatory synapse, twenty-four electron micrographs representing 368.9 μm^2 neuropil regions in each mouse were taken at a 40,000×. Number of spines (PSD density), proportion of perforated spines, PSD length and PSD thickness from each three WT and Dyrk1a-KI mice were quantified by using ImageJ software. For quantification of inhibitory synapse, twenty-four electron micrographs representing 655.5 μm^2 neuropil regions in each mouse were taken at a 30,000×. Number of GABA+ terminals showing clear PSD (inhibitory synapse density), length and thickness of PSD contacting GABA+ terminals from each three WT and Dyrk1a-KI mice were quantified by using ImageJ software. The measurements were all performed by an experimenter blind to the genotype. Digital images were captured with GATAN DigitalMicrograph software driving a CCD camera (SC1000 Orius; Gatan) and saved as TIFF files. Brightness and contrast of the images were adjusted in Adobe Photoshop 7.0 (Adobe Systems).

References for methods

1. Mo J, Kim CH, Lee D, Sun W, Lee HW, Kim H. Early growth response 1 (Egr-1) directly regulates GABAA receptor alpha2, alpha4, and theta subunits in the hippocampus. *J Neurochem* 2015; **133**(4)**:** 489-500.

2. Patro R, Duggal G, Love MI, Irizarry RA, Kingsford C. Salmon provides fast and bias-aware quantification of transcript expression. *Nat Methods* 2017; **14**(4)**:** 417-419.

3. Soneson C, Love MI, Robinson MD. Differential analyses for RNA-seq: transcript-level estimates improve gene-level inferences. *F1000Res* 2015; **4:** 1521.

4. Love MI, Huber W, Anders S. Moderated estimation of fold change and dispersion for RNA-seq data with DESeq2. *Genome Biol* 2014; **15**(12)**:** 550.

5. Subramanian A, Kuehn H, Gould J, Tamayo P, Mesirov JP. GSEA-P: a desktop application for Gene Set Enrichment Analysis. *Bioinformatics* 2007; **23**(23)**:** 3251-3253.

6. Subramanian A, Tamayo P, Mootha VK, Mukherjee S, Ebert BL, Gillette MA *et al.* Gene set enrichment analysis: a knowledge-based approach for

interpreting genome-wide expression profiles. *Proc Natl Acad Sci U S A* 2005; **102**(43)**:** 15545-15550.

7. Isserlin R, Merico D, Voisin V, Bader GD. Enrichment Map - a Cytoscape app to visualize and explore OMICs pathway enrichment results. *F1000Res* 2014; **3:** 141.

8. Merico D, Isserlin R, Stueker O, Emili A, Bader GD. Enrichment map: a network-based method for gene-set enrichment visualization and interpretation. *PLoS One* 2010; **5**(11)**:** e13984.

9. Li J, Van Vranken JG, Pontano Vaites L, Schweppe DK, Huttlin EL, Etienne C *et al.* TMTpro reagents: a set of isobaric labeling mass tags enables simultaneous proteome-wide measurements across 16 samples. *Nat Methods* 2020; **17**(4)**:** 399-404.

10. Eng JK, Jahan TA, Hoopmann MR. Comet: an open-source MS/MS sequence database search tool. *Proteomics* 2013; **13**(1)**:** 22-24.

11. Shteynberg DD, Deutsch EW, Campbell DS, Hoopmann MR, Kusebauch U, Lee D *et al.* PTMProphet: Fast and Accurate Mass Modification Localization for the Trans-Proteomic Pipeline. *J Proteome Res* 2019; **18**(12)**:** 4262-4272.

12. The M, MacCoss MJ, Noble WS, Kall L. Fast and Accurate Protein False Discovery Rates on Large-Scale Proteomics Data Sets with Percolator 3.0. *J Am Soc Mass Spectrom* 2016; **27**(11)**:** 1719-1727.

13. McFarlane HG, Kusek GK, Yang M, Phoenix JL, Bolivar VJ, Crawley JN. Autism-like behavioral phenotypes in BTBR T+tf/J mice. *Genes Brain Behav* 2008; **7**(2)**:** 152-163.

14. Zhan Y, Paolicelli RC, Sforazzini F, Weinhard L, Bolasco G, Pagani F *et al.* Deficient neuron-microglia signaling results in impaired functional brain connectivity and social behavior. *Nat Neurosci* 2014; **17**(3)**:** 400-406.

15. Nadler JJ, Moy SS, Dold G, Trang D, Simmons N, Perez A *et al.* Automated apparatus for quantitation of social approach behaviors in mice. *Genes Brain Behav* 2004; **3**(5)**:** 303-314.

16. Silverman JL, Yang M, Lord C, Crawley JN. Behavioural phenotyping assays for mouse models of autism. *Nat Rev Neurosci* 2010; **11**(7)**:** 490-502.

17. Paik SK, Bae JY, Park SE, Moritani M, Yoshida A, Yeo EJ *et al.* Developmental changes in distribution of gamma-aminobutyric acid- and

glycine-immunoreactive boutons on rat trigeminal motoneurons. I. Jaw-closing motoneurons. *J Comp Neurol* 2007; **503**(6)**:** 779-789.

18. Kolston J, Osen KK, Hackney CM, Ottersen OP, Storm-Mathisen J. An atlas of glycine- and GABA-like immunoreactivity and colocalization in the cochlear nuclear complex of the guinea pig. *Anat Embryol (Berl)* 1992; **186**(5)**:** 443-465.

19. Ottersen OP, Storm-Mathisen J. GABA-containing neurons in the thalamus and pretectum of the rodent. An immunocytochemical study. *Anat Embryol (Berl)* 1984; **170**(2)**:** 197-207.

20. Ottersen OP, Storm-Mathisen J, Madsen S, Skumlien S, Stromhaug J. Evaluation of the immunocytochemical method for amino acids. *Med Biol* 1986; **64**(2-3)**:** 147-158.

Supplementary figure legends

Supplementary Figure 1. Generation and characterization of Dyrk1a-I48K-KI mice

(a) Schematic depiction of the utilized Dyrk1a-I48K-knockin (KI) strategy in mice. The I48K-KI mutation results in the truncation of the DYRK1A protein after amino acid residue 48 in the mutant mice, as in the human case. Ex, exon; Frt, Flp recombinase target site.

(b and c) Immunoblot analysis of DYRK1A protein levels in whole-brain total lysates from heterozygous Dyrk1a-KI mice (P21 for N-term Ab, P56 for C-term), showing a ~50% decrease in DYRK1A protein levels compared with those in wild-type (WT) mice. The DYRK1A antibodies target amino acids 1–50 and 674–763 of the DYRK1 protein (termed DYRK1A-N and DYRK1A-C antibodies, respectively). Note that the use of a high-percentage PAGE gel and the DYRK1A-N antibody does not reveal a small N-terminal fragment of the DYRK1A protein (48 amino acids) with an expected molecular mass of ~5 kDa. (n = 5 mice [WT and KI] for the panel b, Student's t-test).

(d) *In situ* hybridization for *Dyrk1a* mRNAs in the mouse brain at various developmental stages; E/embryonic day; P/postnatal day. Signals are evident in olfactory bulb, cortex, hippocampus, hypothalamus, and cerebellum. Scale bar, 10 mm.

(e) Immunoblot analysis for DYRK1A and PSD-95 (control) proteins in whole-brain total lysates obtained from mice at various developmental stages.

(f) Immunoblot analyses reveal comparable DYRK1A protein levels in different brain

regions of WT mice (P14). mPFC, medial prefrontal cortex; Ctx, cortex; Str, striatum; Hp, hippocampus; Hyp, hypothalamus.

(g and h) Body weights of WT and Dyrk1a-KI male and female mice at various postnatal stages. (n = 16 [male-WT; reduced to 15 at P49], 11 [male-KI], 12 [female-WT; reduced to 11 at P74], 8 [female-KI], two-way ANOVA).

(i) Brain weights and areas and brain/body weight ratios were obtained from WT and Dyrk1a-KI mice at postnatal weeks 3 and 8 (male). Top-down views of the brains were used to measure brain areas. (n = 4 mice [WT, KI] for both age groups, Student's t-test).

(j and k) Largely normal gross morphology of the Dyrk1a-KI brain (2-3 month), as shown by staining of coronal sections for DAPI (cell bodies) + NeuN (neurons) + S100β (astrocytes), or DAPI + neurofilament-M (NF-M; axons). Scale bar, 500 mm (merged and markers).

Significance is indicated as * (<0.05), ** (<0.01), *** (<0.001), or ns (not significant).

Supplementary Figure 2. Behavioral characterization of Dyrk1a-KI mice.

(a) Normal levels of open-field locomotor activity in Dyrk1a-KI mice (2–5 months; male), as shown by total distance moved, mean movement velocity, and time spent in the center region of the arena. (n = 23 mice [WT], 27 [KI], two-way ANOVA).

(b) Normal levels of locomotor activity in Laboras cages among Dyrk1a-KI mice (2–5 months; male), as shown by total distances moved in the light-off and light-on

phases over three consecutive days. (n = 16 [WT], 15 [KI], two-way ANOVA).

(c) Normal levels of anxiety-like behavior in the elevated plus-maze test among Dyrk1a-KI mice (2–5 months; male), as shown by time spent in open/closed arms and number of entries to open arms. (n = 16 [WT], 18 [KI], two-way ANOVA, Mann-Whitney U test).

(d) Normal levels of anxiety-like behavior in the light/dark test among Dyrk1a-KI mice (2–5 months; male), as shown by time spent in the light box. (n = 15 [WT], 19 [KI], Mann-Whitney U test).

(e) Normal motor coordination in the rotarod test among Dyrk1a-KI mice (2–5 months; male), as shown by latency to fall. (n = 14 [WT], 18 [KI], two-way ANOVA).

(f) Normal levels of social interaction in the three-chamber test among Dyrk1a-KI mice (2–5 months; male), as shown by time spent sniffing social/object targets (S1/O) and preference index (S1–O/S1+O). (n = 9 [WT], 10 [KI], two-way ANOVA, Student’s t-test).

(g–i) Decreased repetitive climbing but normal repetitive rearing and self-grooming behavior in Laboras cages among Dyrk1a-KI mice (2–5 months; male), as shown by time spent in the indicated repetitive behaviors during the light-off and light-on periods over three consecutive days (n = 16 [WT], 15 [KI], two-way ANOVA).

Significance is indicated as * (<0.05), ** (<0.01), *** (<0.001), or ns (not significant).

Supplementary Figure 3. Additional behavioral characterization of adult, juvenile, and newborn Dyrk1a-KI mice.

(a and b) Reduced levels of 24-hr contextual fear memory retrieval but normal levels of 7-day contextual fear memory retrieval in Dyrk1a-KI mice (5 months; male), as shown by % freezing during learning/acquisition and retrieval. Memory retrieval was also performed at 7 days without a prior 24-hr retrieval session (b) to exclude the possibility that a 24-hr retrieval may affect the outcome of the 7-day retrieval. Pre, pre-training; post, post-training. (n = 11 mice [WT] and 13 [KI] for 24-hr followed by 7-day retrieval, Student's t-test or Mann-Whitney U test; 16 [WT] and 14 [KI] for 7-day only retrieval; Student's t-test).

(c) Normal levels of cued fear conditioning in Dyrk1a-KI mice (5 months; male), as shown by % freezing during 24-hr retrieval. (n = 16 [WT] and 13 [KI] for 24-hr retrieval, Student's t-test and Mann-Whitney).

(d) Normal levels of contextual spatial learning and memory in the Morris water maze in Dyrk1a-KI mice (4.5 months; male), as shown by time to target/platform in the learning/acquisition phase and time spent in the quadrant in the probe test. Opp, opposite. (n = 12 [WT] and 14 [KI] for learning/acquisition, n = 9 [WT] and 14 [KI] for probe test, two-way ANOVA).

(e) Normal levels of locomotor activity in the open-field test in juvenile Dyrk1a-KI mice (P20–21; males and females mixed), as shown by distance moved, total distance moved, velocity of movement, and time spent in the center region. (n = 35 [WT] and 31 [KI], Mann-Whitney U test or two-way ANOVA).

(f) Normal levels of juvenile play in Dyrk1a-KI mice (~P24; male), as shown by time spent sniffing a social target. (n = 13 pairs [WT-WT] and 15 [KI-KI], Mann-Whitney U test).

(g) Normal levels of repetitive behaviors (self-grooming and digging) in juvenile Dyrk1a-KI mice (~P27; male), as shown by time spent self-grooming/digging. (n = 13 [WT] and 16 [KI], Mann-Whitney U test).

(h) Normal levels of USVs in Dyrk1a-KI pups (P3–9; male and female) separated from their mothers, as shown by the number of USVs and average duration of each USV. (n = 28,28,29,26 [WT] and 22,22,22,21 [KI], respectively, two-way ANOVA).

Significance is indicated as * (<0.05), ** (<0.01), *** (<0.001), or ns (not significant).

Supplementary Figure 4. Normal levels of basal synaptic transmission, presynaptic release, NMDA/AMPA ratios, and synaptic plasticity in Dyrk1a-KI mice.

(a) Normal levels of basal excitatory synaptic transmission, which is mediated by AMPA receptors, at hippocampal Schaffer collateral (SC)-CA1 synapses in Dyrk1a-KI mice (P19–27; male), as shown by input-output curve. (n = 11 neurons from 4 mice [WT], 10, 4 [KI], two-way ANOVA).

(b) Largely normal levels of presynaptic release at hippocampal SC-CA1 synapses in Dyrk1a-KI mice (P21–24; male), as shown by paired-pulse ratios plotted against interstimulus intervals. (n = 11, 4 [WT], 10, 4 [KI], two-way ANOVA).

(c) Normal ratios of NMDA and AMPA receptor-mediated evoked excitatory postsynaptic currents/EPSCs (NMDA/AMPA ratios) at hippocampal SC-CA1 synapses in Dyrk1a-KI mice (P21; female). (n = 13, 9 [WT], 11, 6 [KI], Student's t-test).

(d) Normal high-frequency stimulation-induced long-term potentiation (HFS-LTP) at hippocampal SC-CA1 synapses in Dyrk1a-KI mice (P19–21; male), as shown by the average values of fEPSPs during the final 10 min. (n = 10 slices from 7 mice [WT], 11,6 [KI], Mann-Whitney U test).

(e) Normal theta-burst stimulation-induced long-term potentiation (TBS-LTP) at hippocampal SC-CA1 synapses in Dyrk1a-KI mice (P19–21; male), as shown by average values of fEPSPs during the last 10 min of the recording. (n = 11, 6 [WT], 11, 6 [KI], Student's t-test).

(f) Normal low-frequency stimulation (1 Hz, 15 min)-induced long-term depression (LFS-LTD) at hippocampal SC-CA1 synapses in Dyrk1a-KI mice (P17–22; male), as shown by the average values of fEPSPs during the last 10 min of the recording. (n = 12, 4 [WT], 12, 4 [KI], Student's t-test).

(g) Normal metabotropic glutamate receptor (mGluR)-dependent long-term depression (mGluR-LTD) at hippocampal SC-CA1 synapses in Dyrk1a-KI mice (P18–20; male), as shown by average values of fEPSPs during the last 10 min of the recording. (n = 13, 5 [WT], 7, 4 [KI], Student's t-test).

Significance is indicated as * (<0.05), ** (<0.01), *** (<0.001), or ns (not significant).

Supplementary Figure 5. Transcriptomic changes in WT and Dyrk1a-KI whole brains at P21 and P60.

(a) Volcano plot of differentially expressed genes (DEGs; $p < 0.05$) in the whole-brain transcriptome from Dyrk1a-KI mice (P21; male). (n = 6 mice [WT], 4 [KI]).

(b) List of top DEGs. (n = 6 [WT], 4 [KI]).

(c) Gene set enrichment analysis (GSEA) results showing the enrichments of whole-brain Dyrk1a-KI/WT transcripts at P21 for ASD-related gene sets. Note that ASD-risk genes (SFARI, FMRP targets, De Novo missense, De Novo variants, ASD AutismKB) tend to be downregulated or negatively enriched in ASD. (n = 6 [WT], 4 [KI]).

(d) Clustering of enriched gene sets using the EnrichmentMap Cytoscape App and the GSEA results from Dyrk1a-KI/WT transcripts at P21 derived using gene ontology gene sets (CC, cellular component; BP, biological process). (n = 6 [WT], 4 [KI]).

(e) Volcano plot of differentially expressed genes (DEGs; $p < 0.05$) in the whole-brain transcriptome from Dyrk1a-KI mice (P60; male). (n = 5 mice [WT, KI]).

(f) List of top DEGs. (n = 5 mice [WT, KI]).

(g) Gene set enrichment analysis (GSEA) results showing the enrichments of whole-brain Dyrk1a-KI/WT transcripts at P60 for ASD-related gene sets. (n = 5 mice [WT, KI]).

(h) Clustering of enriched gene sets using the EnrichmentMap Cytoscape App and the GSEA results from Dyrk1a-KI/WT transcripts at P60 derived using gene ontology

gene sets (BP, biological process). (n = 5 mice [WT, KI]).

Supplementary Figure 6. Total-DEPs from vehicle-treated WT and vehicle-treated Dyrk1a-KI mice at P21 and P60.

(a) Volcano plot presentation of P21 total-DEPs ($p < 0.05$ + $FC > 1.2$) from early chronic (P0–21) vehicle-treated WT and vehicle-treated heterozygous Dyrk1a-KI mice, representing baseline differences. (n = 3,5 mice [WT-Veh, KI-Veh]).

(b) Examples of P21 total-DEPs ($p < 0.05$ + $FC > 1.2$; shown in the order of FC) from early chronic (P0–21) vehicle-treated WT and vehicle-treated heterozygous Dyrk1a-KI mice. (n = 3,5 mice [WT-Veh, KI-Veh]).

(c) Volcano plot presentation of P60 total-DEPs ($p < 0.05$ + $FC > 1.2$) from early chronic (P0–28) vehicle-treated WT and vehicle-treated heterozygous Dyrk1a-KI mice, representing baseline differences. (n = 4 mice [WT-Veh, KI-Veh]).

(d) Examples of P60 total-DEPs ($p < 0.05$ + $FC > 1.2$; shown in the order of FC) from early chronic (P0–28) vehicle-treated WT and vehicle-treated heterozygous Dyrk1a-KI mice. (n = 4 mice [WT-Veh, KI-Veh]).

Supplementary Figure 7. PTM-DEPPs in early vehicle-treated WT mice versus early vehicle-treated Dyrk1a-KI mice at ~P21.

(a) Volcano plot showing P21 PTM-DEPPs from early (P0-21) vehicle-treated WT versus early vehicle-treated heterozygous Dyrk1a-KI mice, representing baseline

differences. (n = 3, 5 mice [WT-Veh, KI-Veh]).

(b) Examples of P21 PTM-DEPPs ($p < 0.05$ + FC > 1.2; shown in the order of FC) from early (P0–21) vehicle-treated WT versus vehicle-treated heterozygous Dyrk1a-KI mice. (n = 3, 5 mice [WT-Veh, KI-Veh]).

(c) DAVID-KEGG/gene ontology (GO) analysis of P21 PTM-DEPPs (up and down were pooled) from early (P0–21) vehicle-treated WT versus vehicle-treated heterozygous Dyrk1a-KI mice. (n = 3, 5 mice [WT-Veh, KI-Veh]).

(d) SynGO analysis of P21 PTM-DEPPs (up and down pooled) from early (P0–21) vehicle-treated WT versus vehicle-treated heterozygous Dyrk1a-KI mice. (n = 3, 5 mice [WT-Veh, KI-Veh]).

Supplementary Figure 8. Rescue of some P21 and P60 PTM-DEPPs by early lithium treatment of Dyrk1a-KI mice shown by SynGO analyses

(a) Examples of lithium-rescued P21 PTM-DEPPs (shown in the order of FC).

(b) SynGO analysis of the lithium-rescued P21 PTM-DEPPs (up and down pooled). (n = 3, 4, 5, 4 mice [WT-Veh, WT-Li, KI-Veh, and KI-Li]).

(c) Examples of early lithium-rescued PTM-DEPPs (shown in the order of FC) at ~P60.

(d) SynGO analyses of early lithium-rescued ~P60 PTM-DEPPs (up and down pooled). (n = 4 mice [WT-Veh, WT-Li, KI-Veh, and KI-Li]).

Supplementary Figure 9. PTM-DEPPs derived from the comparison of early vehicle-treated WT and vehicle-treated Dyrk1a-KI mice at ~P60.

(a) Volcano plot presentation of PTM-DEPPs ($p < 0.05$ + FC > 1.2) from early (P0–28) vehicle-treated WT and vehicle-treated heterozygous Dyrk1a-KI mice at ~P60, representing baseline differences. (n = 4 mice [WT-Veh, KI-Veh]).

(b) Examples of PTM-DEPPs ($p < 0.05$ + FC > 1.2; shown in the order of FCs) from early (P0–28) vehicle-treated WT and vehicle-treated heterozygous Dyrk1a-KI mice at ~P60. (n = 4 mice [WT-Veh, KI-Veh]).

(c) DAVID-KEGG/GO analyses of PTM-DEPPs (up and down pooled) from early (P0–28) vehicle-treated WT and vehicle-treated heterozygous Dyrk1a-KI mice at ~P60. (n = 4 mice [WT-Veh, KI-Veh]).

(d) SynGO analyses of PTM-DEPPs (up and down pooled) from early (P0–28) vehicle-treated WT and vehicle-treated heterozygous Dyrk1a-KI mice at ~P60. (n = 4 mice [WT-Veh, KI-Veh]).

Supplementary Figure 10. PTM-DEPPs from naïve homozygous Dyrk1a-KI mice at ~P60.

(a and b) Volcano plot showing PTM-DEPPs ($p < 0.05$ + FC > 1.2) from naïve WT and homozygous/HM Dyrk1a-KI mice (~P60; male and female mixed due to the very low birth rate of homozygous mutant mice). Additional details on PTM-DEPPs with stronger changes ($p < 0.05$ + FC > 1.8) are listed in the table with details on specific phosphorylation sites (b). (n = 3 mice [WT, KI]).

(c) DAVID analyses of P60 PTM-DEPPs (up and down pooled) from naïve homozygous Dyrk1a-KI mice. (n = 3 mice [WT, KI]).

(d) SynGO analysis of P60 PTM-DEPPs (up and down pooled) from naïve homozygous Dyrk1a-KI mice. (n = 3 mice [WT, KI]).

Supplementary Figure 11. Functional characterization of lithium-rescued PTM-DEPPs in Dyrk1a-KI mice.

(a) Total and GSK3β-Ser9-phosphorylation levels of in the brains of P21 WT, Dyrk1a-HT (KI), and lithium-treated Dyrk1a-HT (KI-Li) mice determined by immunoblot analyses. β-Actin was used as a loading control. (n = 3 mice [WT, KI, and KI-Li], one-way ANOVA).

(b) Lack of changes in the phosphorylation states of potential upstream kinases for GSK3β-Ser9 phosphorylation in the PTM-DEPPs from P21 and P60 Dyrk1a-HT mice. Top kinases in the indicated kinase groups with strong possibility (> 99%) to act as a kinase for GSK3β-Ser9 phosphorylation were examined in the PTM-DEPP results to see if there are any changes in their phosphorylations. ns, not significant.

(c and d) List of potential upstream kinases, including DYRK1A and GSK3β, for the top downregulated PTM-DEPPs from P21/P60 Dyrk1a-HT mice, as shown by the pie graphs of upstream kinase groups (left) and some of these kinases (DYRK1A and GSK3β) and their potential substrates identified from the PTM-DEPP results (right). Note that Kalirin represents a strong substrate candidate with neuronal/synaptic functions for both Dyrk1a and GSK3β (indicated as D and G, respectively) and that

Elavl2, although with relatively weaker D/G kinase scores, have neuronal/synaptic functions, leading us to try functional characterization.

(e and f) Effects of the overexpression of Kalirin-7-S448D/A (phospho-mimic/nonphosphorylatable) and Elavl2-S221D/A (phospho-mimic/nonphosphorylatable) mutants in cultured WT and Dyrk1a-HT hippocampal neurons (days in vitro/DIV 10 to DIV 8), as shown by the characterization of neuronal arborization by Sholl analysis of neuronal immunostaining for AAV-syn-mcherry which was cotransfected with each construct. (n = 3 independent experiments [WT/KI-Kalirin-S448D/A and WT/KI-Elavl2-S221D/A], two-way ANOVA with Bonferroni's test).

Significance is indicated as * (<0.05), ** (<0.01), *** (<0.001), or ns (not significant).

Supplementary Figure 12. Full-length raw images of the immunoblot results.

Tables

Supplementary Table 1. Statistical details.

Supplementary Table 2. Transcriptomic data from WT and Dyrk1a-KI whole brains at P21 (DEG list, and GSEA results).

Supplementary Table 3. Transcriptomic data from WT and Dyrk1a-KI whole brains at P60 (DEG list, and GSEA results).

Supplementary Table 4. List of P21 and P60 total-DEPs derived from vehicle-

treated WT and vehicle-treated Dyrk1a-KI mice.

Supplementary Table 5. List of baseline P21 PTM-DEPPs derived from vehicle-treated WT and vehicle-treated Dyrk1a-KI mice, and P21 lithium-rescued PTM-DEPPs derived from vehicle-treated WT and early lithium-treated Dyrk1a-KI mice.

Supplementary Table 6. List of baseline P60 PTM-DEPPs derived from vehicle-treated WT and vehicle-treated Dyrk1a-KI mice, and P60 lithium-rescued PTM-DEPPs derived from vehicle-treated WT and early lithium-treated Dyrk1a-KI mice.

Supplementary Table 7. List of P60 PTM-DEPPs derived from naïve WT and homozygous Dyrk1a-KI mice.

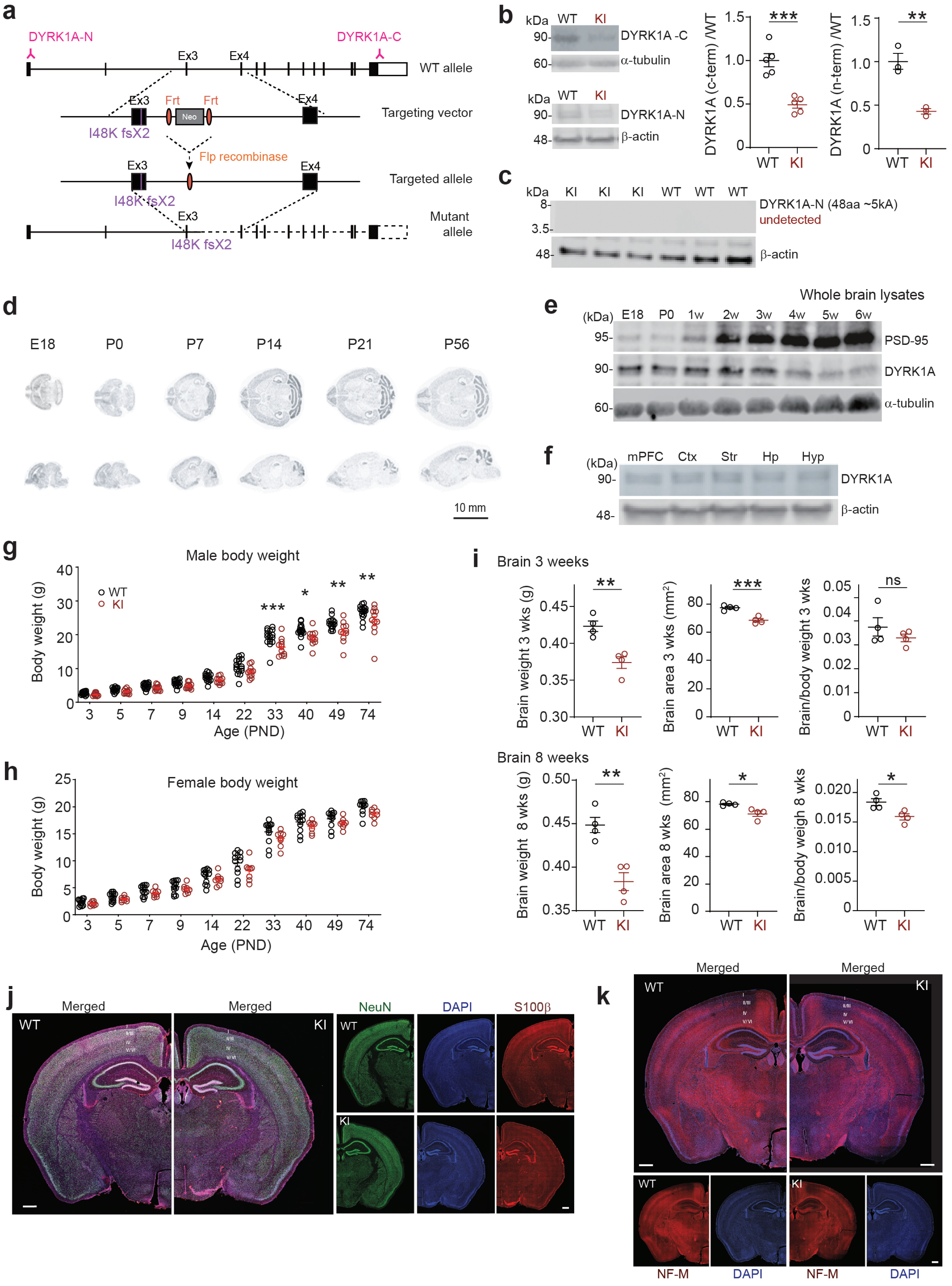
a
DYRK1A-N
DYRK1A-C
Ex3
Ex4
WT allele
Frt
Neo
Targeting vector
I48K fsX2
Flp recombinase
Targeted allele
Mutant allele
b
kDa WT KI
90- DYRK1A -C
60- α-tubulin
90- DYRK1A-N
48- β-actin
DYRK1A (c-term) /WT
DYRK1A (n-term) /WT
***
**
c
kDa KI KI KI WT WT WT
8-
3.5-
DYRK1A-N (48aa ~5kA)
undetected
48- β-actin
d
E18 P0 P7 P14 P21 P56
10 mm
e
Whole brain lysates
(kDa) E18 P0 1w 2w 3w 4w 5w 6w
95- PSD-95
90- DYRK1A
60- α-tubulin
f
(kDa) mPFC Ctx Str Hp Hyp
90- DYRK1A
48- β-actin
g
Male body weight
Body weight (g)
Age (PND)
WT
KI
h
Female body weight
Body weight (g)
Age (PND)
i
Brain 3 weeks
Brain weight 3 wks (g)
Brain area 3 wks (mm²)
Brain/body weight 3 wks
ns
Brain 8 weeks
Brain weight 8 wks (g)
Brain area 8 wks (mm²)
Brain/body weigh 8 wks
j
Merged
WT
KI
NeuN
DAPI
S100β
k
Merged
NF-M
DAPI

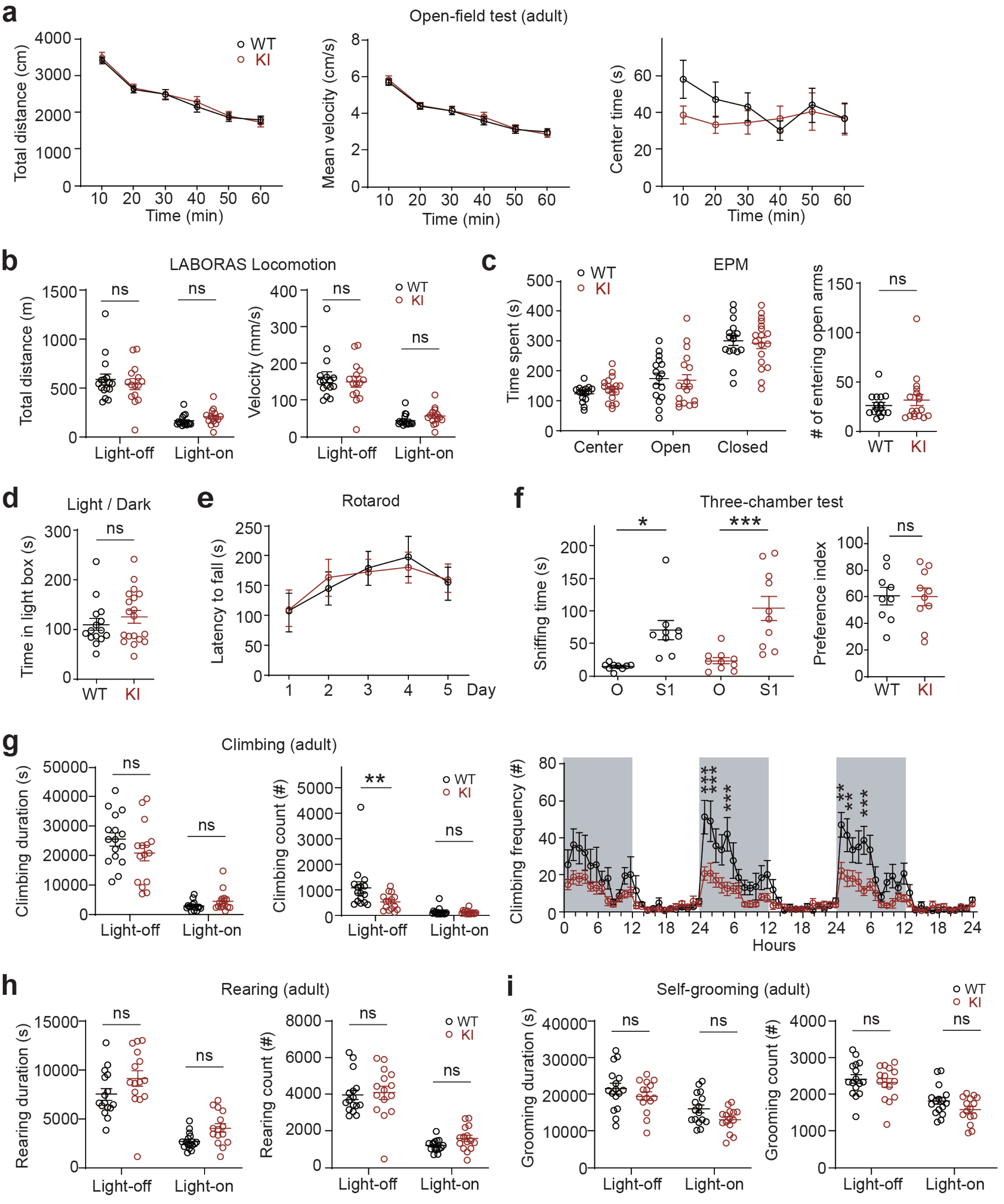
a
Open-field test (adult)
Total distance (cm)
Time (min)
WT
KI
Mean velocity (cm/s)
Center time (s)
b
LABORAS Locomotion
Total distance (m)
ns
Light-off
Light-on
Velocity (mm/s)
c
EPM
Time spent (s)
Center
Open
Closed
# of entering open arms
d
Light / Dark
Time in light box (s)
e
Rotarod
Latency to fall (s)
Day
f
Three-chamber test
Sniffing time (s)
O
S1
Preference index
g
Climbing (adult)
Climbing duration (s)
Climbing count (#)
Climbing frequency (#)
Hours
h
Rearing (adult)
Rearing duration (s)
Rearing count (#)
i
Self-grooming (adult)
Grooming duration (s)
Grooming count (#)

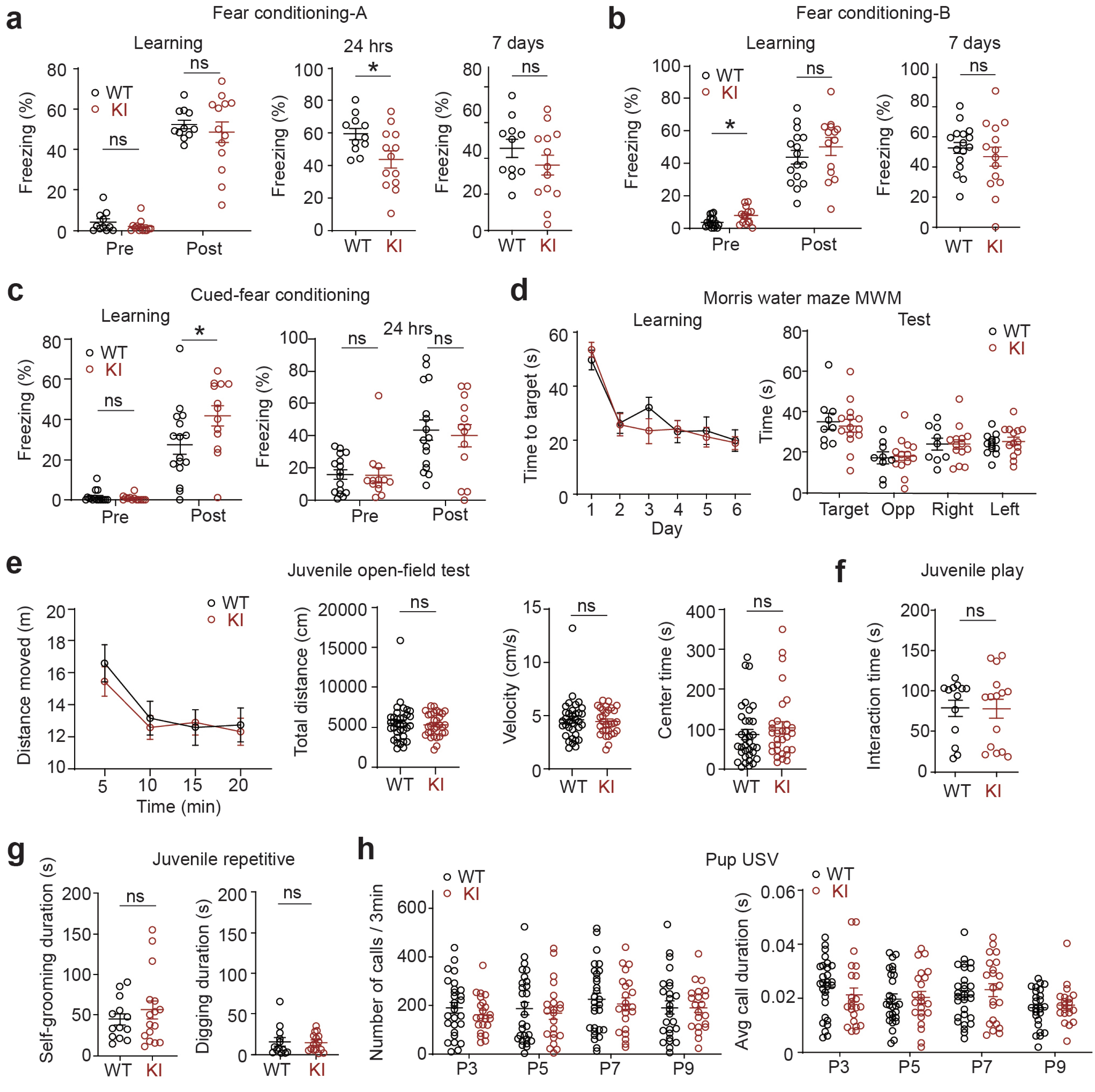

a
Fear conditioning-A
Learning
24 hrs
7 days
Freezing (%)
WT
KI
ns
*
Pre
Post
b
Fear conditioning-B
c
Cued-fear conditioning
d
Morris water maze MWM
Test
Time to target (s)
Day
Time (s)
Target
Opp
Right
Left
e
Juvenile open-field test
Distance moved (m)
Time (min)
Total distance (cm)
Velocity (cm/s)
Center time (s)
f
Juvenile play
Interaction time (s)
g
Juvenile repetitive
Self-grooming duration (s)
Digging duration (s)
h
Pup USV
Number of calls / 3min
Avg call duration (s)
P3
P5
P7
P9

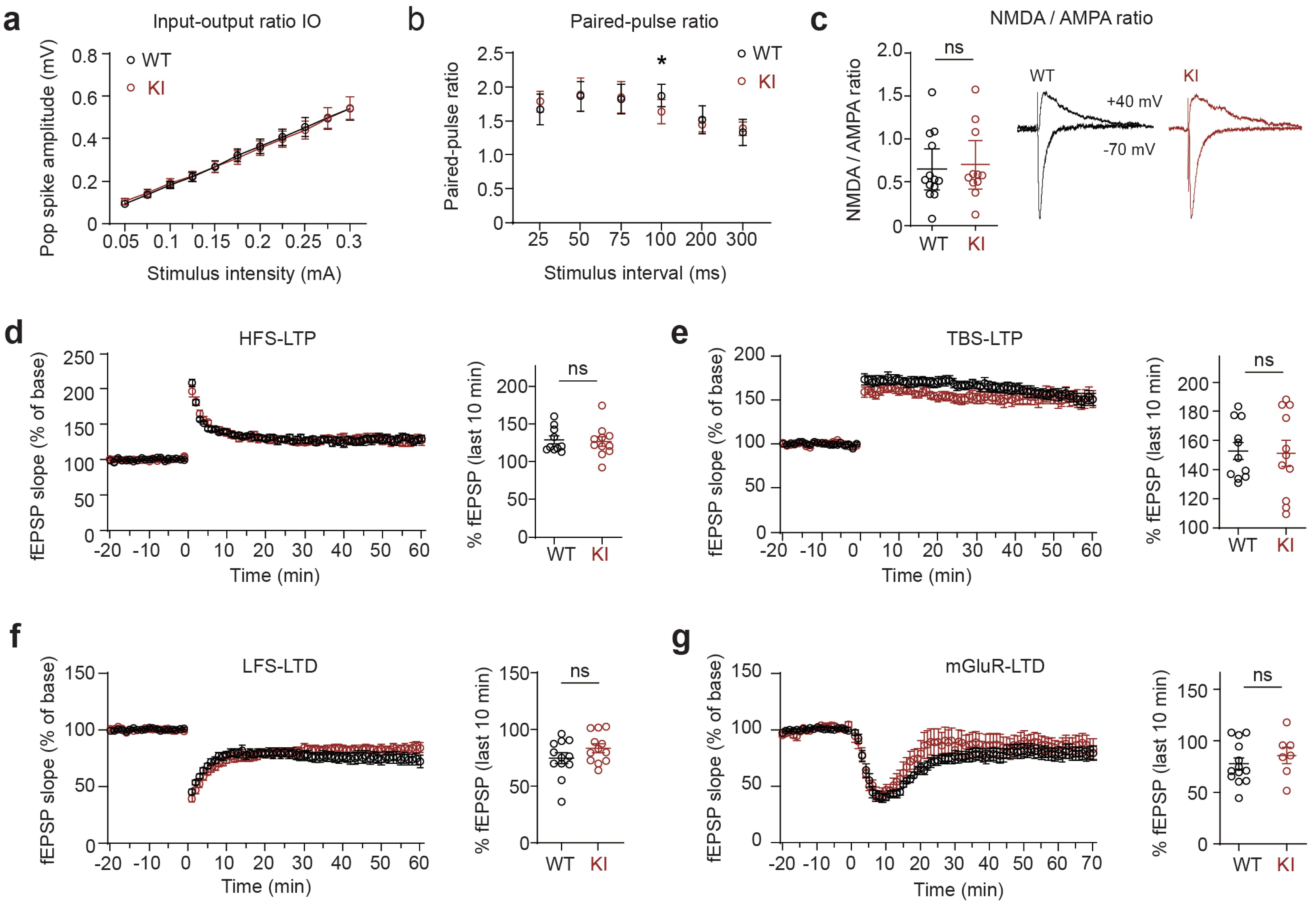

a
Input-output ratio IO
Pop spike amplitude (mV)
Stimulus intensity (mA)
WT
KI
b
Paired-pulse ratio
Paired-pulse ratio
Stimulus interval (ms)
WT
KI
c
NMDA / AMPA ratio
NMDA / AMPA ratio
ns
WT
KI
+40 mV
-70 mV
d
HFS-LTP
fEPSP slope (% of base)
Time (min)
% fEPSP (last 10 min)
ns
e
TBS-LTP
f
LFS-LTD
g
mGluR-LTD

a

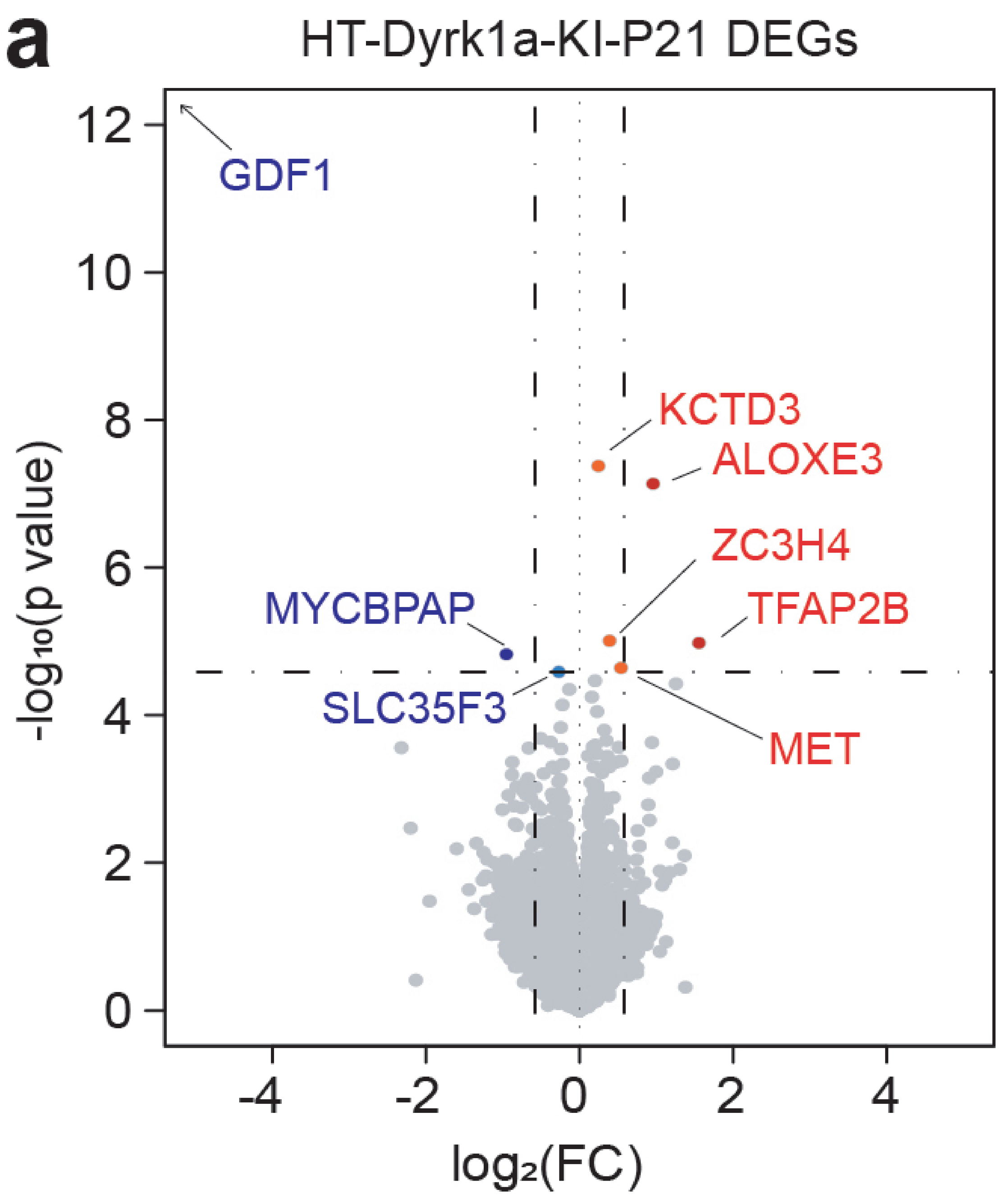

b

Gene name	Fold change	p-value	Full name
GDF1	-2.47E+06	5.15E-14	Growth differentiation factor 1
KCTD3	1.19	4.21E-08	K^+ channel tet. domain containing 3
ALOXE3	1.95	7.33E-08	Arachidonate lipoxygenase 3
ZC3H4	1.31	9.81E-06	Zinc finger CCCH-type containing 4
TFAP2B	2.94	1.05E-05	Transcription factor AP-2 beta
MYCBPAP	-1.93	1.50E-05	MYCBP associated protein
MET	1.46	2.29E-05	MET proto-oncogene, receptor Y kinase
SLC35F3	-1.20	2.59E-05	Solute carrier family 35 member F3

c

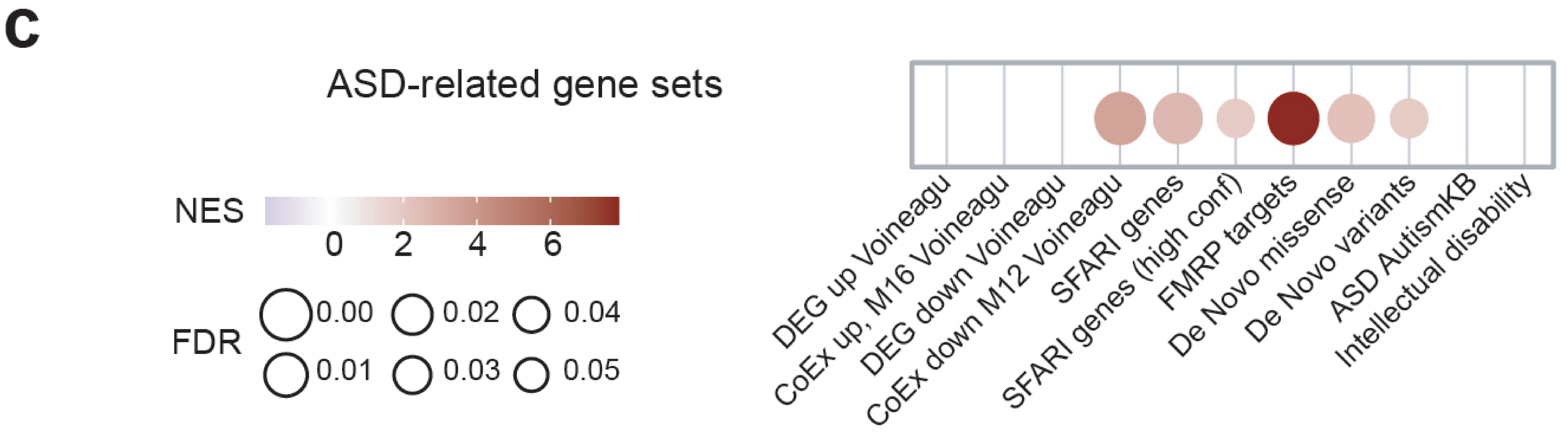

d

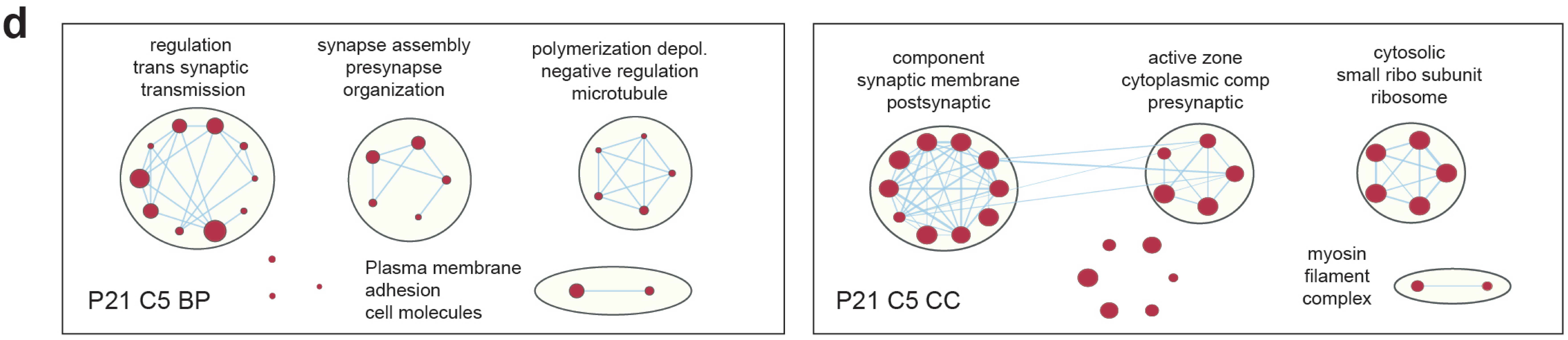

e

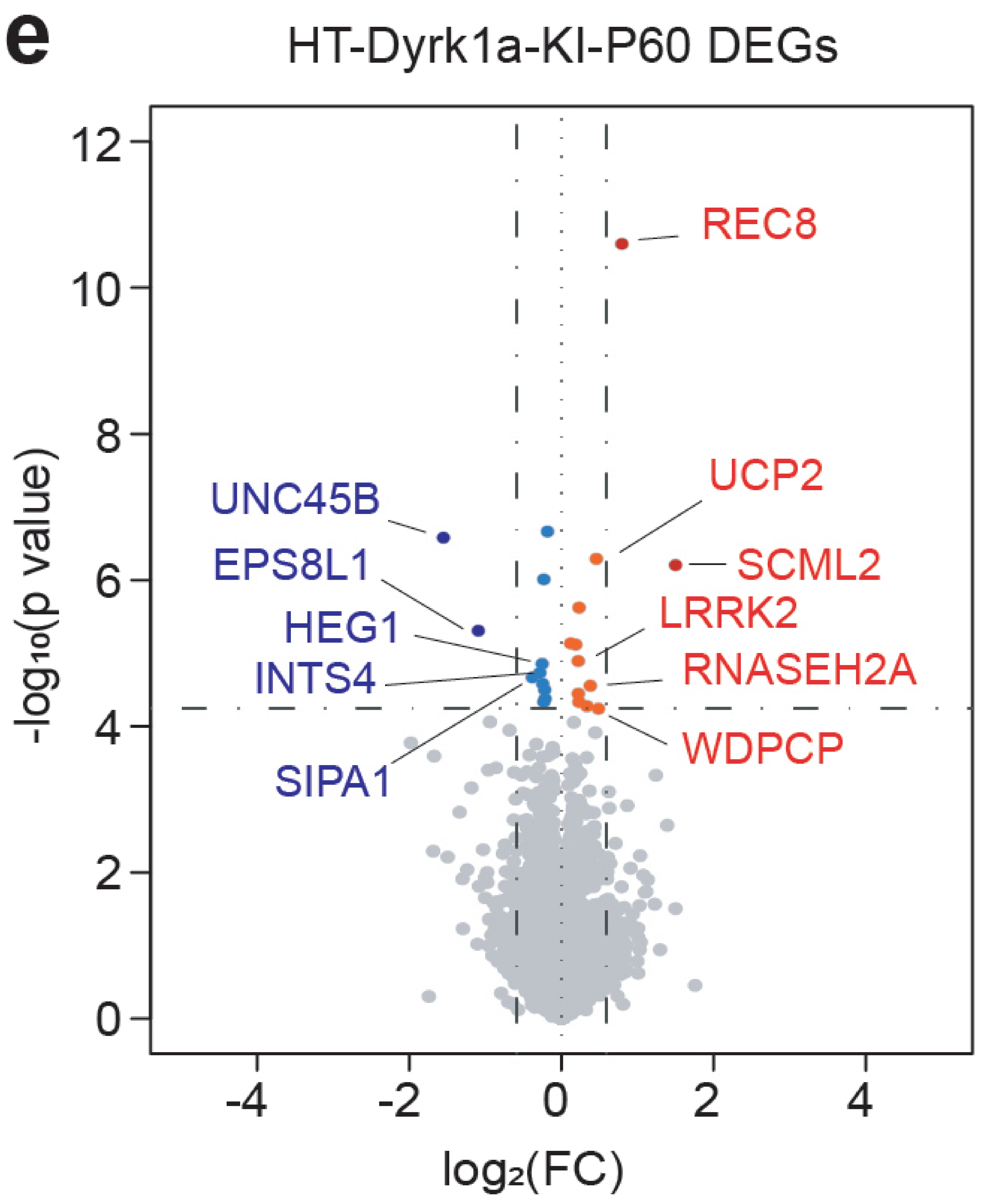

f

Gene name	Fold change	p-value	Full name
REC8	1.74	2.52E-11	REC8 meiotic recombination protein
UNC45B	-2.93	2.63E-07	unc-45 myosin chaperone B
UCP2	1.37	5.12E-07	uncoupling protein 2
SCML2	2.83	6.24E-07	Scm polycomb group protein like2
EPS8L1	-2.13	4.93E-06	EPS8 like1
HEG1	-1.19	1.40E-05	heart development protein with EGF like domains 1
INTS4	-1.22	1.86E-05	integrator complex subunit 4
SIPA1	-1.30	2.15E-05	signal-induced proliferation-associated 1
LRRK2	1.30	2.79E-05	leucine rich repeat kinase 2
RNASEH2A	1.26	5.31E-05	ribonuclease H2 subunit A
WDPCP	1.40	5.74E-05	WD repeat containing planar cell polarity effector

g

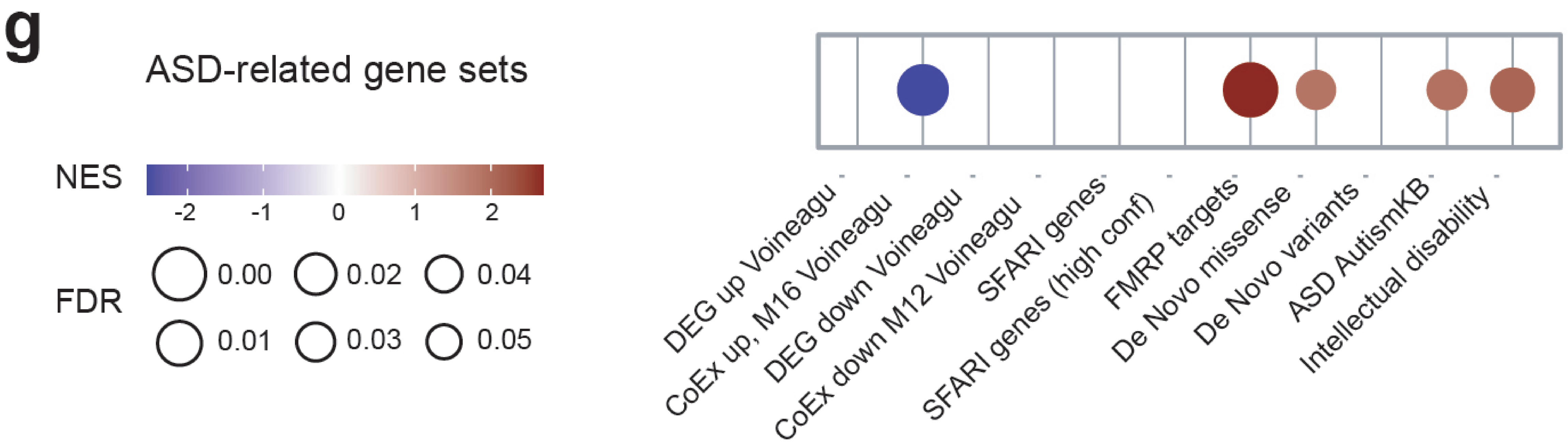

h

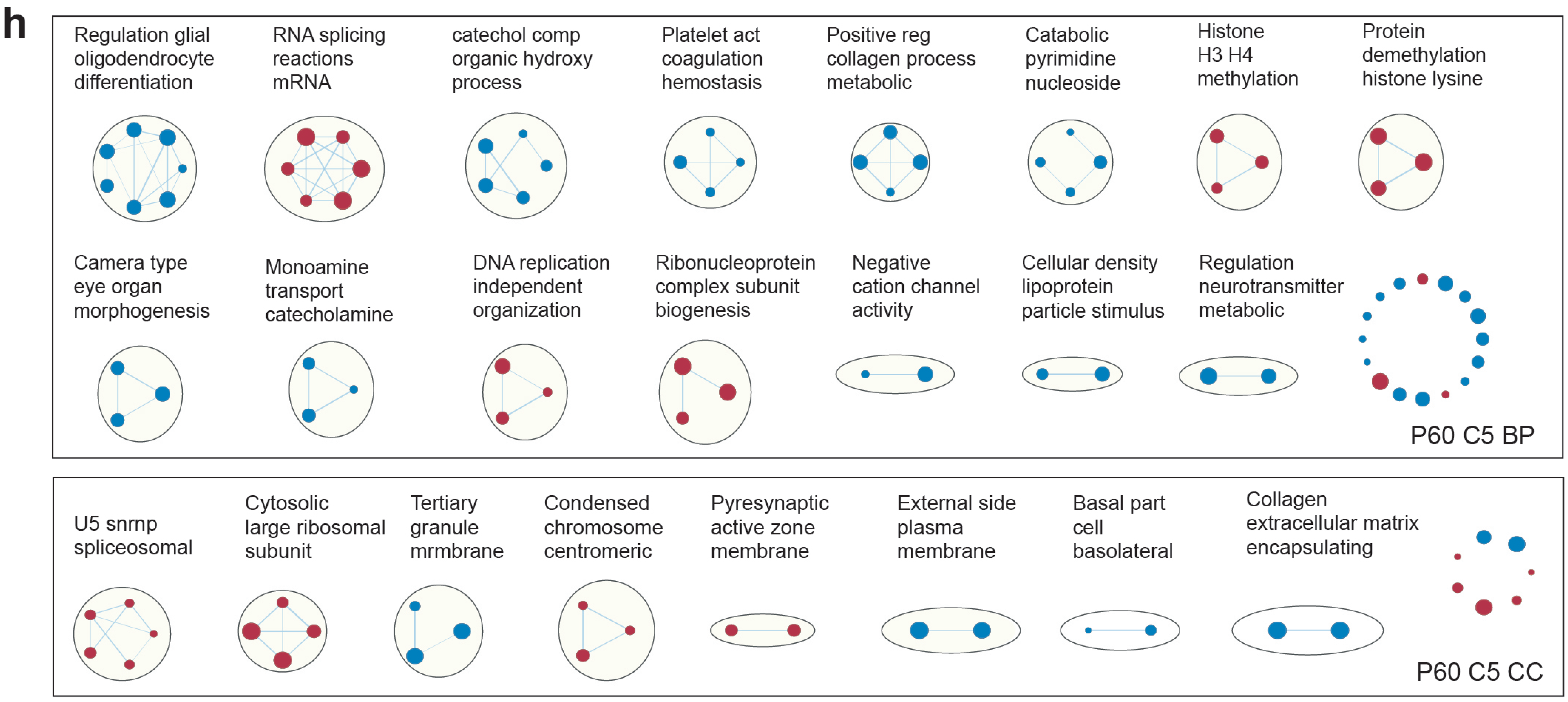

a

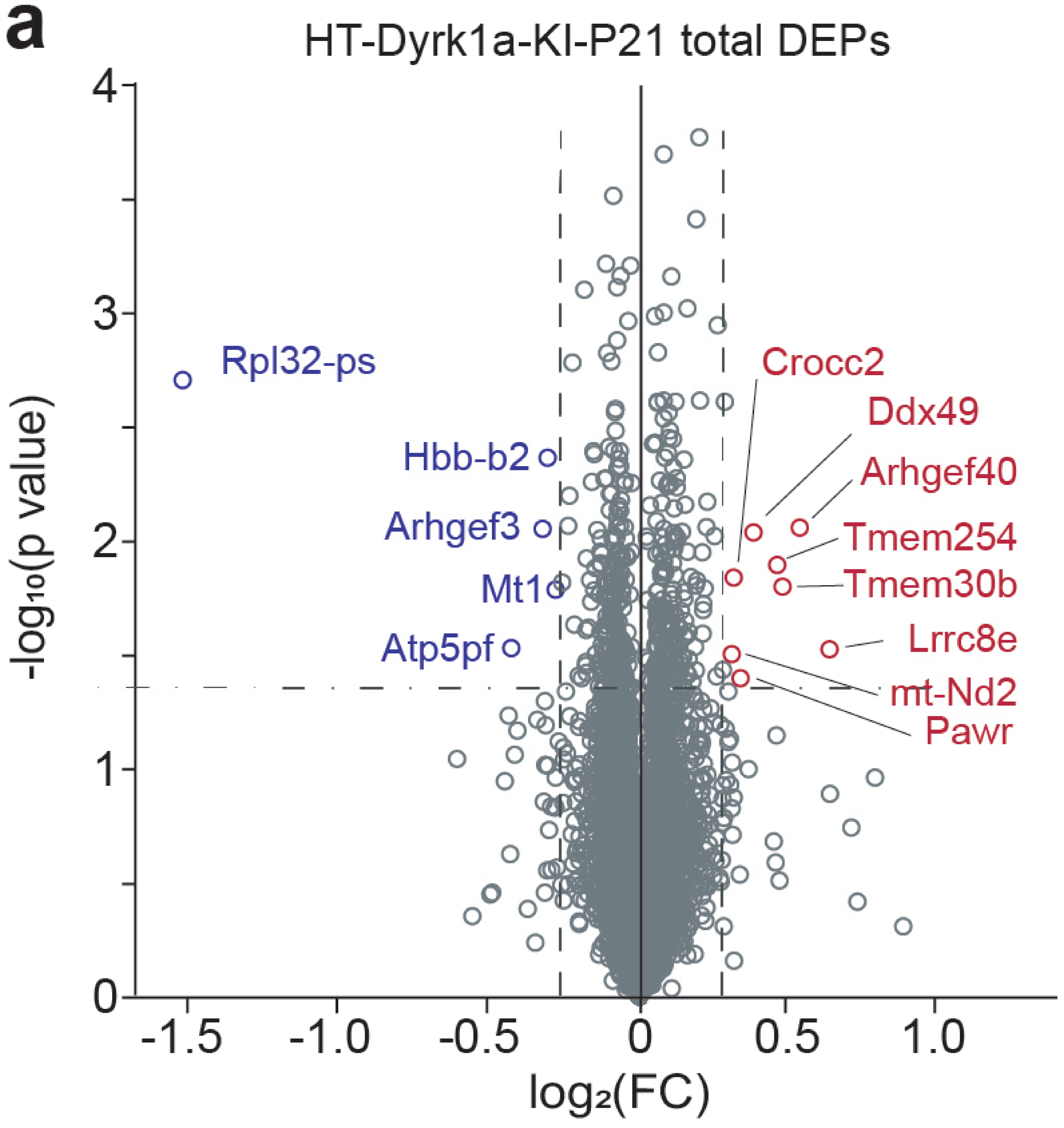

b

Top DEPs in P21 Dyrk1a KI proteome vs WT

Gene name	FC	p-value	Full name
Lrrc8e	1.554	1.554	Leucine-rich repeat-containing protein 8E
Arhgef40	1.451	1.451	Rho guanine nucleotide exchange factor 40
Tmem30b	1.395	1.395	Transmembrane protein 30b
Tmem254	1.376	1.376	Transmembrane protein254
Ddx49	1.304	1.304	DEAD-box hlicase 49
Pawr	1.265	1.265	Pro-apoptotic WT1 regulator
Crocc2	1.245	1.245	Ciliary rootlet coiled-coil protein 2
mt-Nd2	1.239	1.239	Mito-encoded NADH dehydrogenase subunit2
Rpl32-ps	-2.882	0.002	Ribosomal protein L32, pseudogene
Atp5pf	-1.345	0.030	ATP synthase peripheral stalk subunit F6
Arhgef3	-1.252	0.009	Rho guanine nucleotide exchange factor 3
Hbb-b2	-1.237	0.004	Hemoglobin subunit beta2
Mt1	-1.214	0.016	Metallothionein 1

c

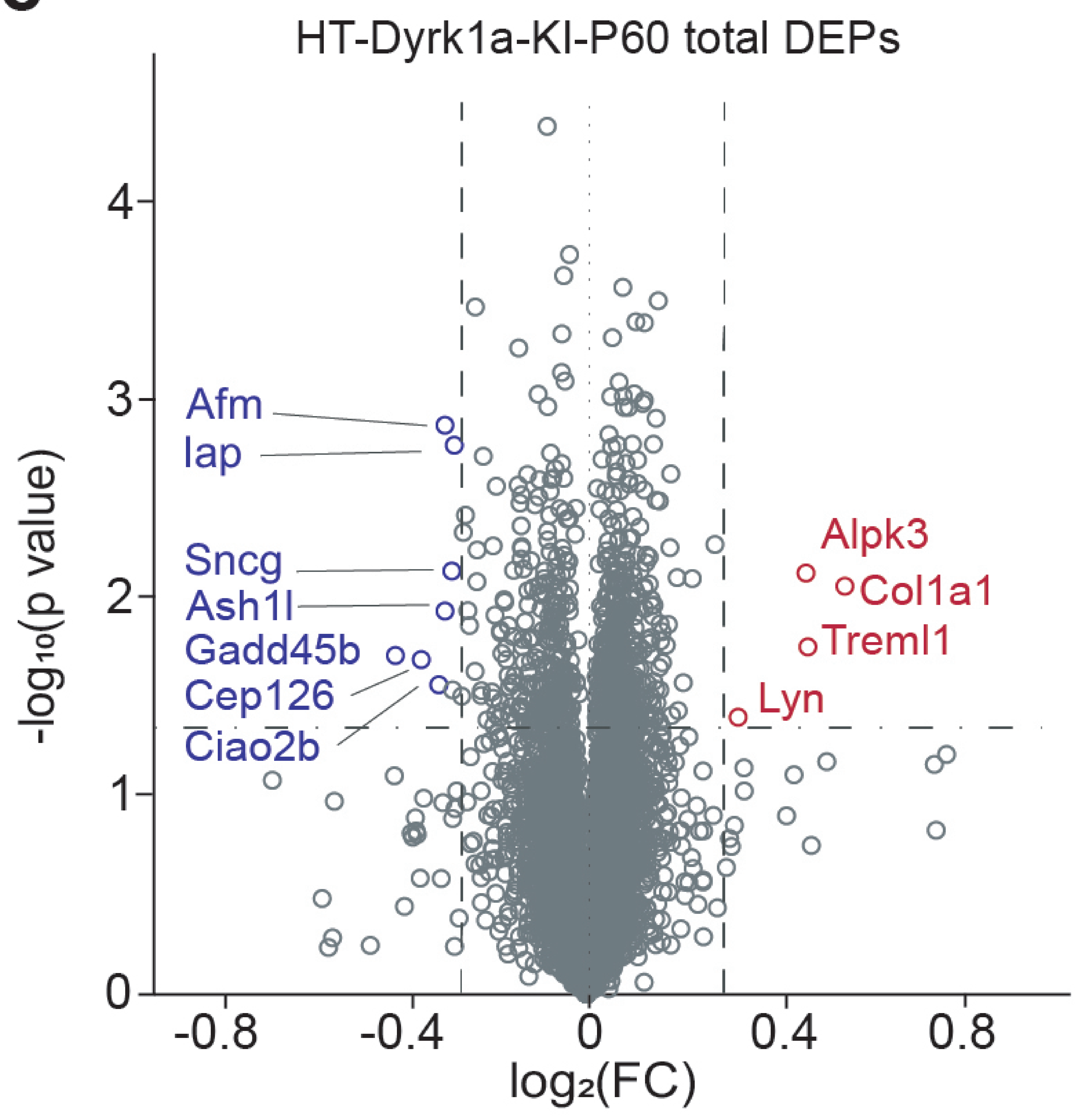

d

Top DEPs in P60 Dyrk1a KI proteome vs WT

Gene name	FC	p-value	Full name
Col1a1	1.447	0.009	Collagen alpha-1(I) chain
Col1a2	1.372	0.017	Collagen alpha-2(I) chain
Alpk3	1.369	0.007	Alpha-protein kinase 3
Lyn	1.242	0.039	Tyrosine-protein kinase Lyn
Treml1	1.201	0.005	Trem-like transcript 1 protein
Iap	-1.209	0.002	IgE-binding protein
Ciao2b	-1.212	0.029	Cytosolic iron-sulfur assembly component 2B
Sncg	-1.213	0.007	Gamma-synuclein
Alb	-1.225	0.011	Albumin
Afm	-1.225	0.001	Afamin
Cep126	-1.236	0.027	Centrosomal protein of 126
Ash1l	-1.267	0.020	Histone-lysine N-methyltransferase
Gadd45b	-1.315	0.019	Growth arrest and DNA damage-inducible protein

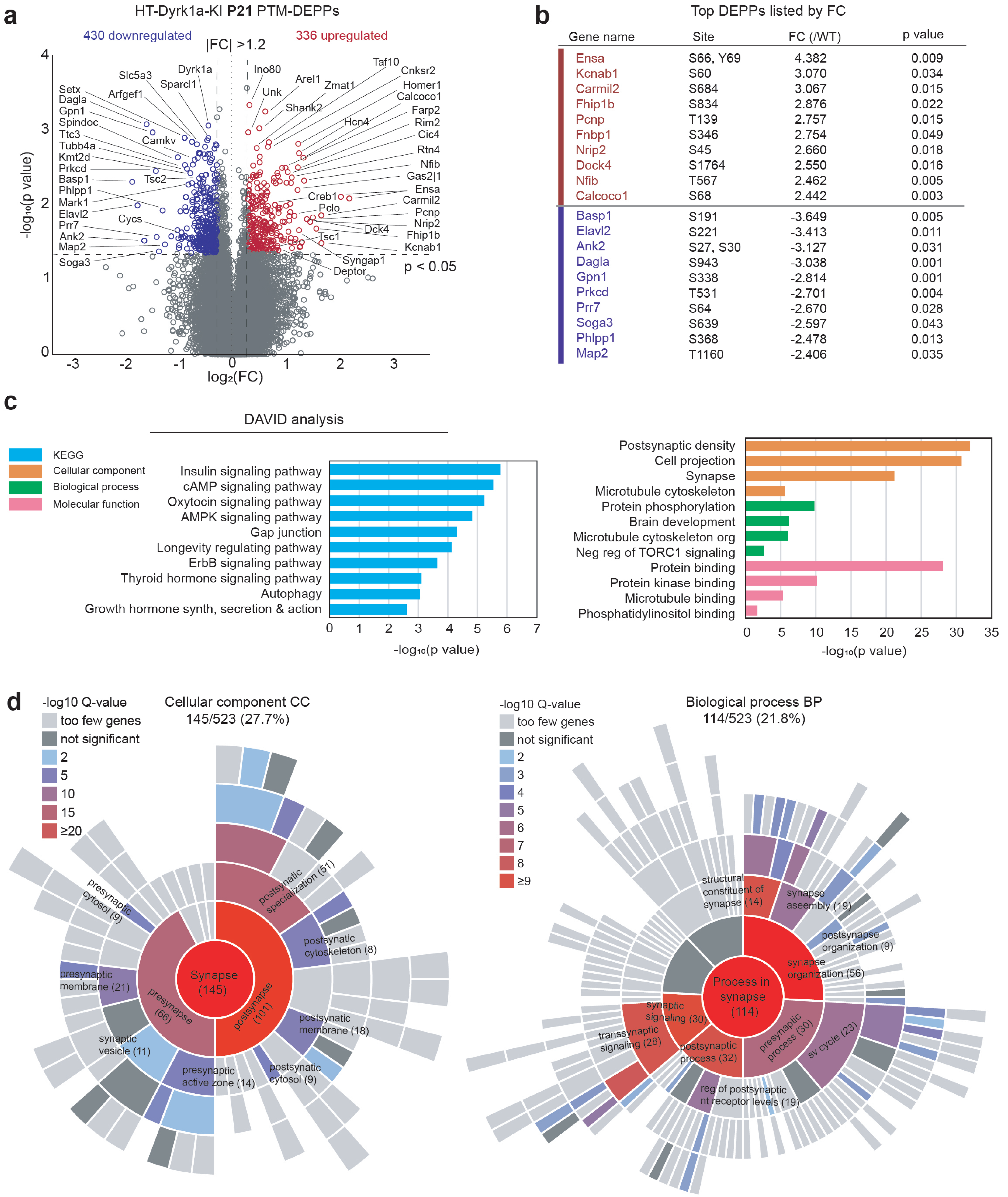

Gene name	Site	FC (/WT)	p value
Ensa	S66, Y69	4.382	0.009
Kcnab1	S60	3.070	0.034
Carmil2	S684	3.067	0.015
Fhip1b	S834	2.876	0.022
Pcnp	T139	2.757	0.015
Fnbp1	S346	2.754	0.049
Nrip2	S45	2.660	0.018
Dock4	S1764	2.550	0.016
Nfib	T567	2.462	0.005
Calcoco1	S68	2.442	0.003
Basp1	S191	-3.649	0.005
Elavl2	S221	-3.413	0.011
Ank2	S27, S30	-3.127	0.031
Dagla	S943	-3.038	0.001
Gpn1	S338	-2.814	0.001
Prkcd	T531	-2.701	0.004
Prr7	S64	-2.670	0.028
Soga3	S639	-2.597	0.043
Phlpp1	S368	-2.478	0.013
Map2	T1160	-2.406	0.035

a Top DEPPs rescued by lithium treatment P21 (by ΔFC)

Gene name	Site	FC (/WT)	p value	FC (/WT)	p value
Smg7	S1010	2.239	0.005	1.289	0.087
Cic	S1510	1.994	0.004	1.075	0.617
Cpeb2	S246	2.162	0.048	1.257	0.544
Naxd	Y36	2.068	0.033	1.187	0.206
Calcoco1	S68	2.442	0.003	1.577	0.233
Sp9	T12	2.259	0.047	1.542	0.304
Tjp1	Y898	1.917	0.029	1.218	0.138
Map1a	T1292	1.628	0.014	-1.063	0.447
Kmt2c	S694	1.763	0.004	1.087	0.617
Phc3	T261	1.678	0.019	1.007	0.553
Taf10	S44	1.571	0.002	-1.082	0.694
FAM120A	S432	1.514	0.008	-1.107	0.796
Elavl2	S221	-3.413	0.011	-1.054	0.287
Map2	T1160, S1191	-2.406	0.035	-1.070	0.797
Mast2	S149	-1.743	0.029	-1.037	0.871
Necab1	S14	-1.669	0.032	-1.077	0.982
Pds5a	S1171	-1.854	0.011	-1.271	0.496
Hectd1	S482	-1.858	0.027	-1.277	0.173
Atp1a3	Y45	-1.606	0.006	-1.037	0.652
Aff4	S698	-1.654	0.020	-1.091	0.568
Ank1	S433	-1.947	0.030	-1.394	0.174
Soga3	S288	-1.650	0.035	-1.112	0.441
Cd2bp2	T244	-1.572	0.033	-1.036	0.574
Kalrn	S488	-1.692	0.009	-1.177	0.073

c Top DEPPs rescued by lithium treatment P60 (by ΔFC)

Gene name	Site	FC (/WT)	p value	FC (/WT)	p value
Bsn	T2595	1.997	0.011	1.048	0.900
Rin1	Y35	2.214	0.020	1.432	0.208
Tppp	S34	1.353	0.029	0.898	0.483
Ap1s1	S147	1.466	0.011	1.033	0.871
Dock10	S292	1.238	0.020	0.930	0.476
Sv2b	T36,Y46	1.283	0.016	0.982	0.789
Marcks	T79	1.536	0.014	1.248	0.257
Mapre2	T201	1.298	0.006	1.021	0.840
H1-4	S41, T35	1.236	0.034	0.998	0.974
Atxn2l	S499	1.373	0.029	1.140	0.277
Cap2	S261	1.235	0.047	1.029	0.765
Sucla2	S279	1.583	0.043	1.380	0.105
Kcnc1	S468	-1.360	0.041	1.064	0.726
Mia3	S1693	-1.397	0.025	-1.087	0.562
Camsap2	S647	-1.596	0.012	-1.310	0.141
Prrc2c	S1965	-1.245	0.018	1.014	0.799
Tjp2	S380	-1.257	0.019	-1.030	0.776
Ctnnd2	T525	-1.465	0.023	-1.248	0.121
Znf827	S757	-1.227	0.039	-1.047	0.463
Aftph	S284	-1.222	0.012	-1.043	0.653
Acsbg1	S70	-1.298	0.046	-1.124	0.360
Fam117b	T104	-1.269	0.041	-1.102	0.293
Pak3	S227	-1.241	0.028	-1.077	0.316
Tsc1	T640	-1.217	0.018	-1.055	0.443

b

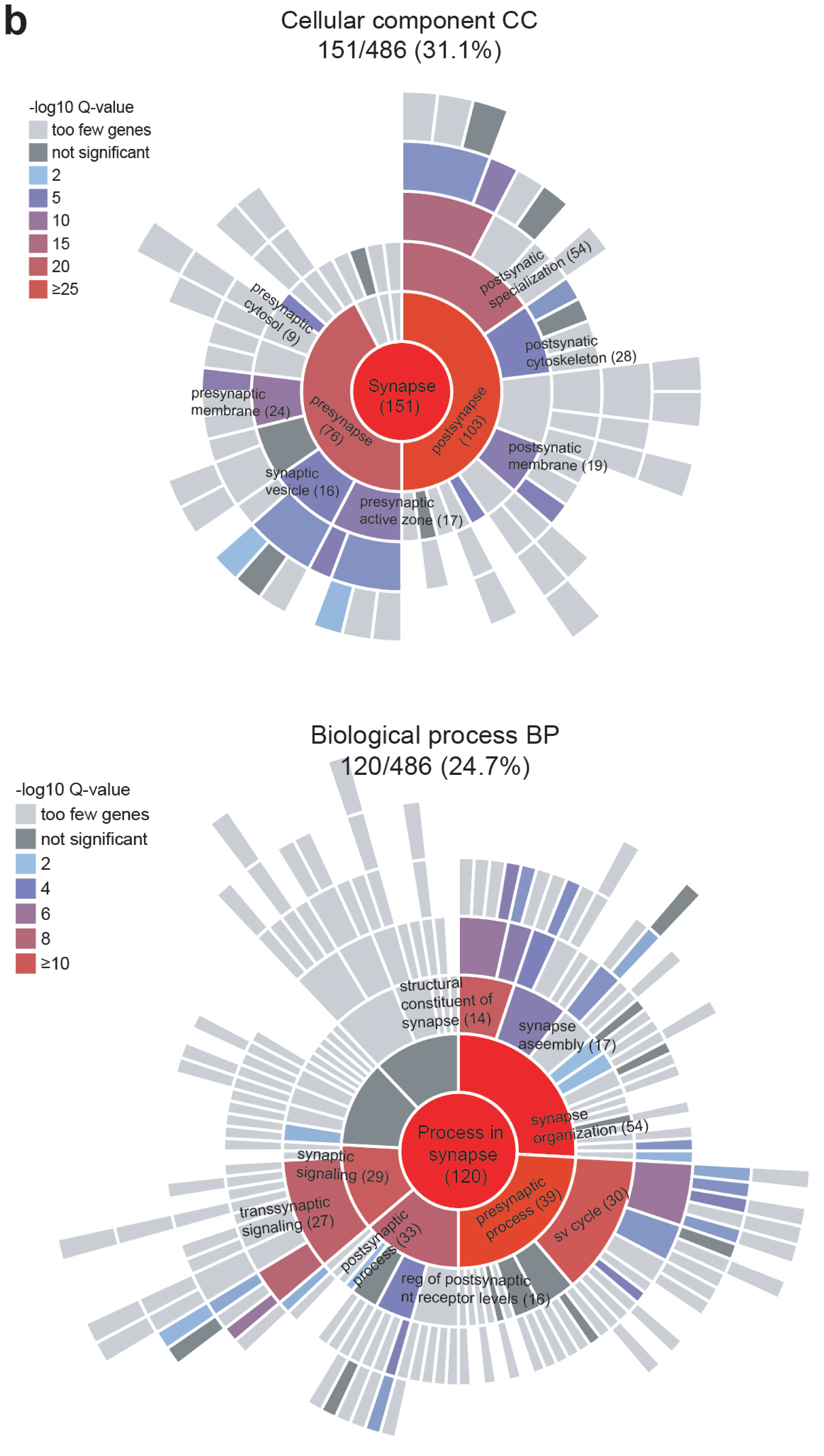

d

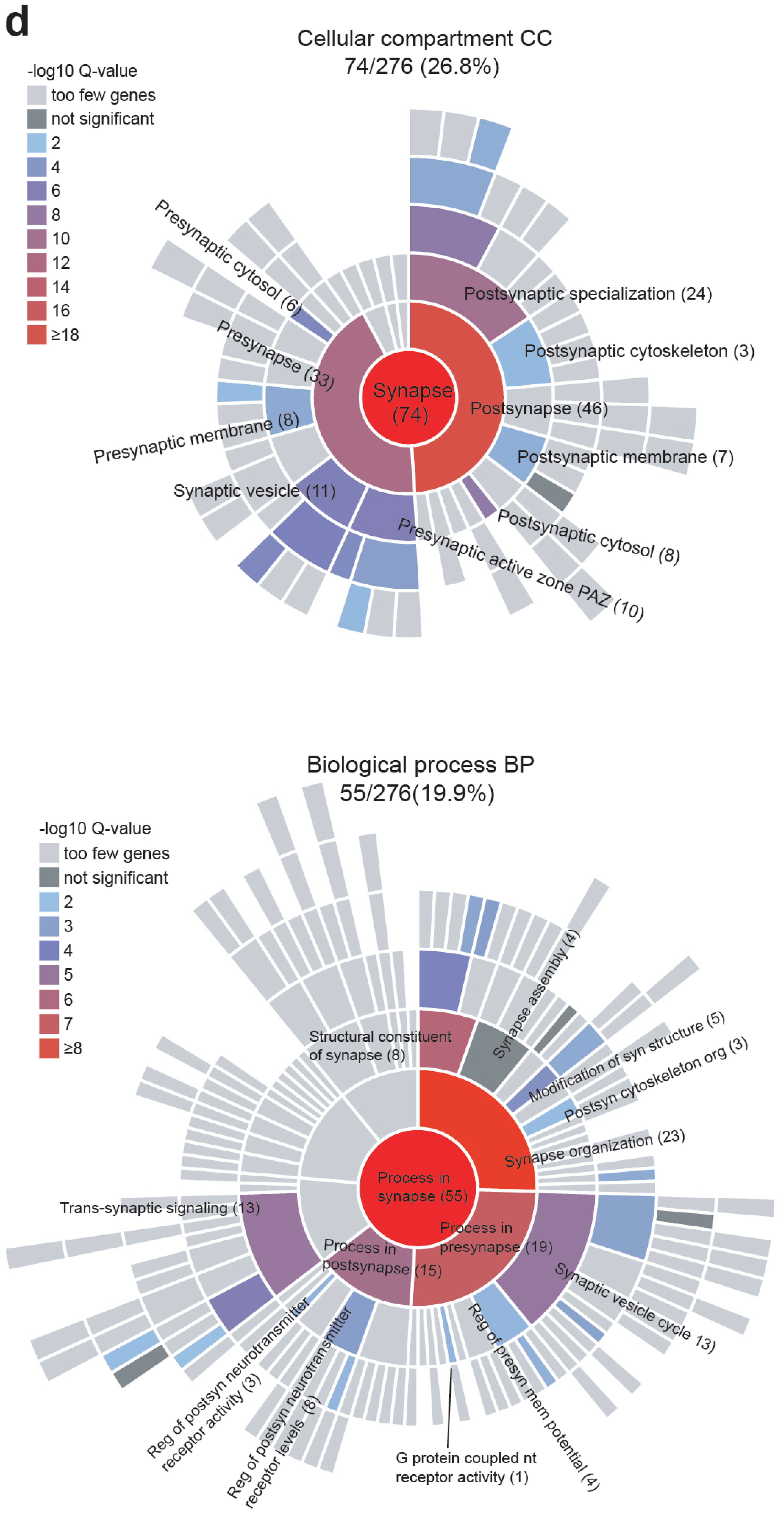

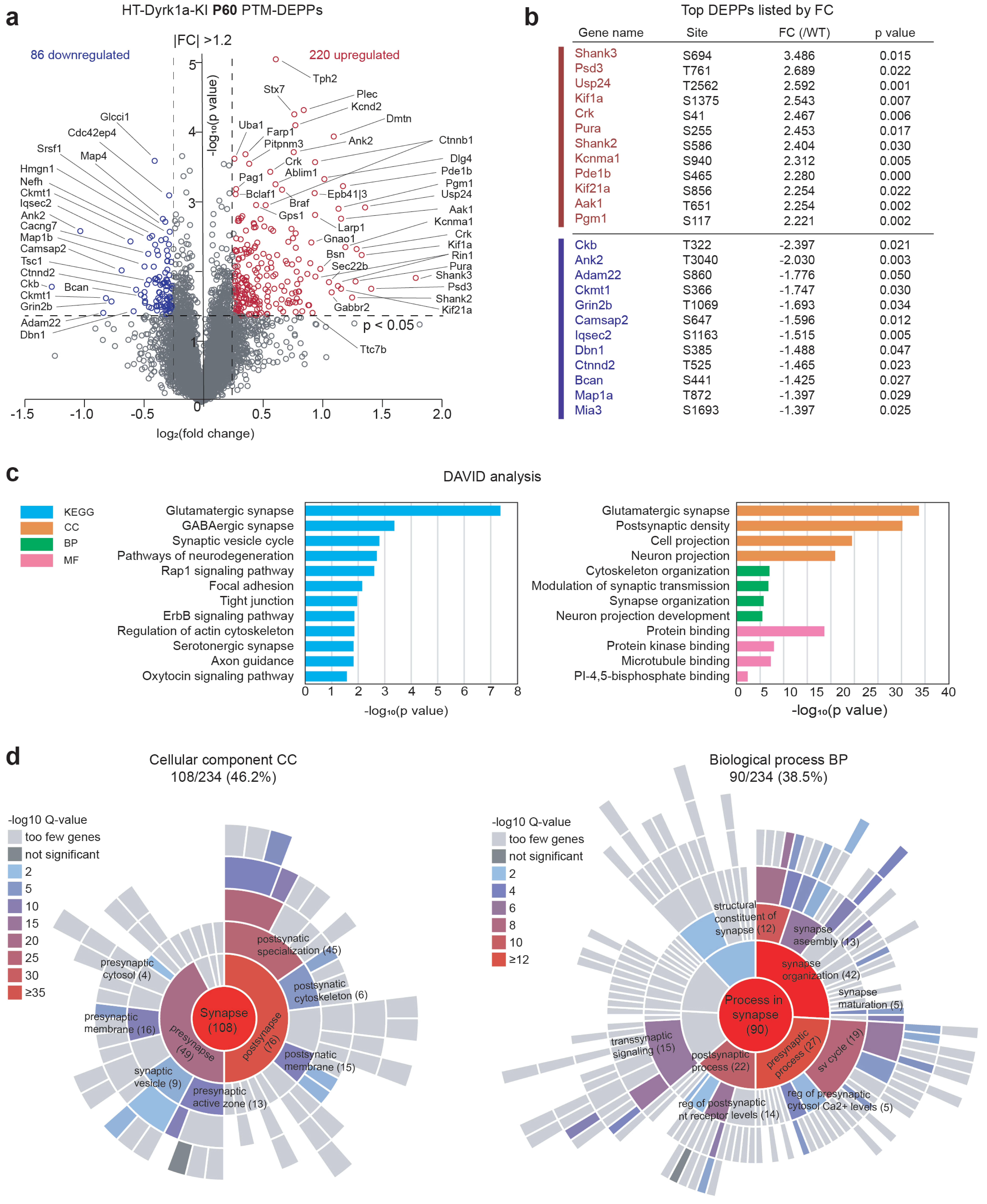
a
HT-Dyrk1a-KI P60 PTM-DEPPs
|FC| >1.2
86 downregulated
220 upregulated
-log₁₀(p value)
log₂(fold change)
p < 0.05
Tph2
Stx7
Plec
Kcnd2
Dmtn
Uba1
Farp1
Pitpnm3
Ank2
Ctnnb1
Crk
Dlg4
Ablim1
Pde1b
Pag1
Pgm1
Bclaf1
Epb41l3
Usp24
Braf
Gps1
Aak1
Larp1
Kcnma1
Gnao1
Crk
Kif1a
Bsn
Rin1
Sec22b
Pura
Shank3
Psd3
Shank2
Gabbr2
Kif21a
Ttc7b
Glcci1
Cdc42ep4
Srsf1
Map4
Hmgn1
Nefh
Ckmt1
Iqsec2
Ank2
Cacng7
Map1b
Camsap2
Tsc1
Ctnnd2
Ckb
Bcan
Ckmt1
Grin2b
Adam22
Dbn1
b
Top DEPPs listed by FC
Gene name | Site | FC (/WT) | p value
Shank3 | S694 | 3.486 | 0.015
Psd3 | T761 | 2.689 | 0.022
Usp24 | T2562 | 2.592 | 0.001
Kif1a | S1375 | 2.543 | 0.007
Crk | S41 | 2.467 | 0.006
Pura | S255 | 2.453 | 0.017
Shank2 | S586 | 2.404 | 0.030
Kcnma1 | S940 | 2.312 | 0.005
Pde1b | S465 | 2.280 | 0.000
Kif21a | S856 | 2.254 | 0.022
Aak1 | T651 | 2.254 | 0.002
Pgm1 | S117 | 2.221 | 0.002
Ckb | T322 | -2.397 | 0.021
Ank2 | T3040 | -2.030 | 0.003
Adam22 | S860 | -1.776 | 0.050
Ckmt1 | S366 | -1.747 | 0.030
Grin2b | T1069 | -1.693 | 0.034
Camsap2 | S647 | -1.596 | 0.012
Iqsec2 | S1163 | -1.515 | 0.005
Dbn1 | S385 | -1.488 | 0.047
Ctnnd2 | T525 | -1.465 | 0.023
Bcan | S441 | -1.425 | 0.027
Map1a | T872 | -1.397 | 0.029
Mia3 | S1693 | -1.397 | 0.025
c
DAVID analysis
KEGG
CC
BP
MF
Glutamatergic synapse
GABAergic synapse
Synaptic vesicle cycle
Pathways of neurodegeneration
Rap1 signaling pathway
Focal adhesion
Tight junction
ErbB signaling pathway
Regulation of actin cytoskeleton
Serotonergic synapse
Axon guidance
Oxytocin signaling pathway
-log₁₀(p value)
Glutamatergic synapse
Postsynaptic density
Cell projection
Neuron projection
Cytoskeleton organization
Modulation of synaptic transmission
Synapse organization
Neuron projection development
Protein binding
Protein kinase binding
Microtubule binding
PI-4,5-bisphosphate binding
-log₁₀(p value)
d
Cellular component CC
108/234 (46.2%)
-log10 Q-value
too few genes
not significant
2
5
10
15
20
25
30
≥35
Synapse (108)
postsynapse (76)
presynapse (49)
postsynaptic specialization (45)
postsynaptic cytoskeleton (6)
postsynaptic membrane (15)
presynaptic cytosol (4)
presynaptic membrane (16)
synaptic vesicle (9)
presynaptic active zone (13)
Biological process BP
90/234 (38.5%)
-log10 Q-value
too few genes
not significant
2
4
6
8
10
≥12
Process in synapse (90)
structural constituent of synapse (12)
synapse assembly (13)
synapse organization (42)
synapse maturation (5)
transsynaptic signaling (15)
postsynaptic process (22)
presynaptic process (27)
sv cycle (19)
reg of postsynaptic nt receptor levels (14)
reg of presynaptic cytosol Ca2+ levels (5)

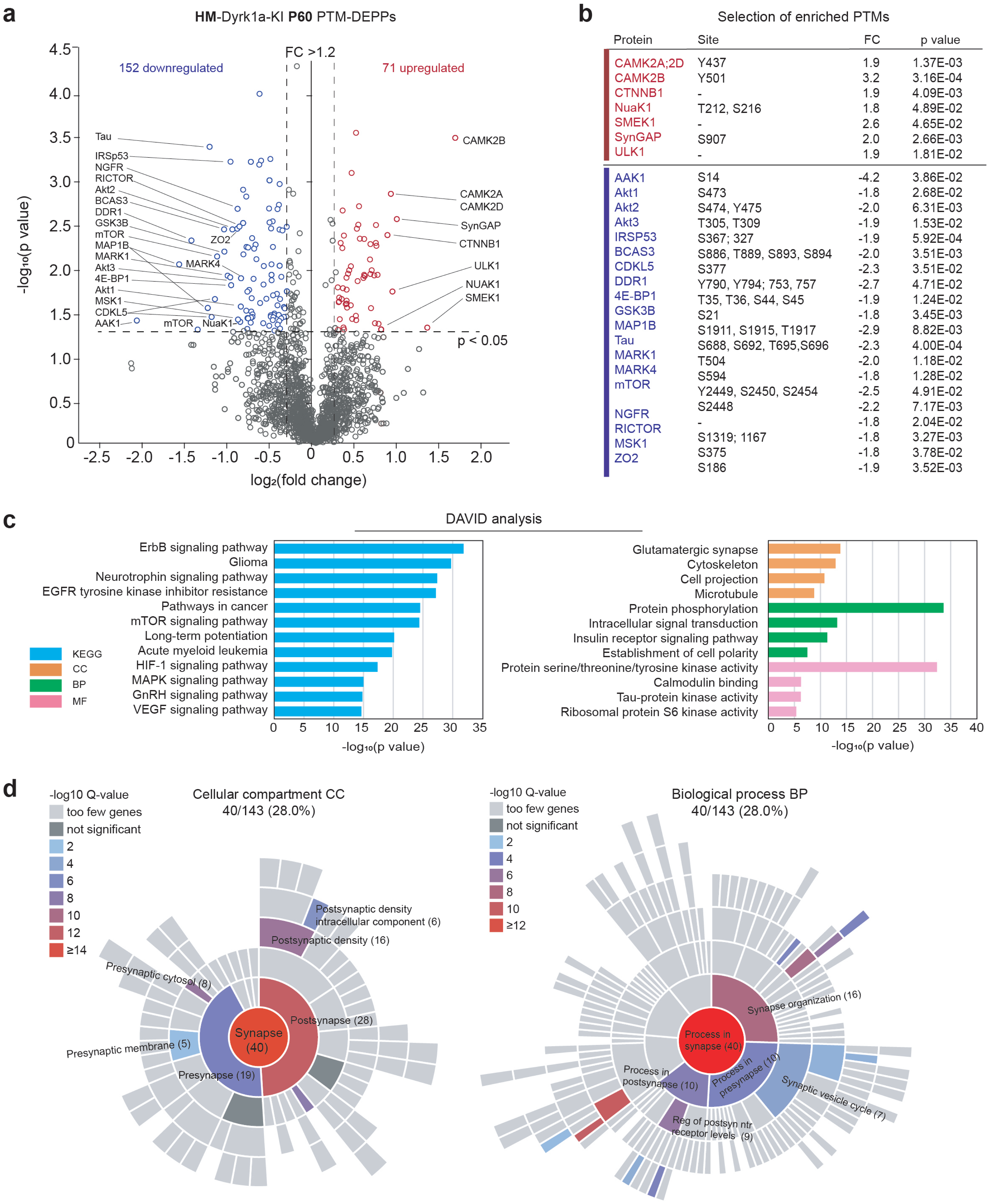

a
HM-Dyrk1a-KI P60 PTM-DEPPs
152 downregulated
71 upregulated
FC >1.2
p < 0.05
-log₁₀(p value)
log₂(fold change)
Tau
IRSp53
NGFR
RICTOR
Akt2
BCAS3
DDR1
GSK3B
mTOR
MAP1B
MARK1
Akt3
4E-BP1
Akt1
MSK1
CDKL5
AAK1
ZO2
MARK4
mTOR
NuaK1
CAMK2B
CAMK2A
CAMK2D
SynGAP
CTNNB1
ULK1
NUAK1
SMEK1
b
Selection of enriched PTMs
Protein Site FC p value
CAMK2A;2D Y437 1.9 1.37E-03
CAMK2B Y501 3.2 3.16E-04
CTNNB1 - 1.9 4.09E-03
NuaK1 T212, S216 1.8 4.89E-02
SMEK1 - 2.6 4.65E-02
SynGAP S907 2.0 2.66E-03
ULK1 - 1.9 1.81E-02
AAK1 S14 -4.2 3.86E-02
Akt1 S473 -1.8 2.68E-02
Akt2 S474, Y475 -2.0 6.31E-03
Akt3 T305, T309 -1.9 1.53E-02
IRSP53 S367; 327 -1.9 5.92E-04
BCAS3 S886, T889, S893, S894 -2.0 3.51E-03
CDKL5 S377 -2.3 3.51E-02
DDR1 Y790, Y794; 753, 757 -2.7 4.71E-02
4E-BP1 T35, T36, S44, S45 -1.9 1.24E-02
GSK3B S21 -1.8 3.45E-03
MAP1B S1911, S1915, T1917 -2.9 8.82E-03
Tau S688, S692, T695,S696 -2.3 4.00E-04
MARK1 T504 -2.0 1.18E-02
MARK4 S594 -1.8 1.28E-02
mTOR Y2449, S2450, S2454 -2.5 4.91E-02
S2448 -2.2 7.17E-03
NGFR - -1.8 2.04E-02
RICTOR S1319; 1167 -1.8 3.27E-03
MSK1 S375 -1.8 3.78E-02
ZO2 S186 -1.9 3.52E-03
c
DAVID analysis
KEGG
CC
BP
MF
ErbB signaling pathway
Glioma
Neurotrophin signaling pathway
EGFR tyrosine kinase inhibitor resistance
Pathways in cancer
mTOR signaling pathway
Long-term potentiation
Acute myeloid leukemia
HIF-1 signaling pathway
MAPK signaling pathway
GnRH signaling pathway
VEGF signaling pathway
-log₁₀(p value)
Glutamatergic synapse
Cytoskeleton
Cell projection
Microtubule
Protein phosphorylation
Intracellular signal transduction
Insulin receptor signaling pathway
Establishment of cell polarity
Protein serine/threonine/tyrosine kinase activity
Calmodulin binding
Tau-protein kinase activity
Ribosomal protein S6 kinase activity
-log₁₀(p value)
d
-log10 Q-value
too few genes
not significant
2
4
6
8
10
12
≥14
Cellular compartment CC
40/143 (28.0%)
Synapse (40)
Postsynapse (28)
Presynapse (19)
Postsynaptic density (16)
Postsynaptic density intracellular component (6)
Presynaptic cytosol (8)
Presynaptic membrane (5)
-log10 Q-value
too few genes
not significant
2
4
6
8
10
≥12
Biological process BP
40/143 (28.0%)
Process in synapse (40)
Synapse organization (16)
Process in presynapse (10)
Process in postsynapse (10)
Synaptic vesicle cycle (7)
Reg of postsyn ntr receptor levels (9)

a

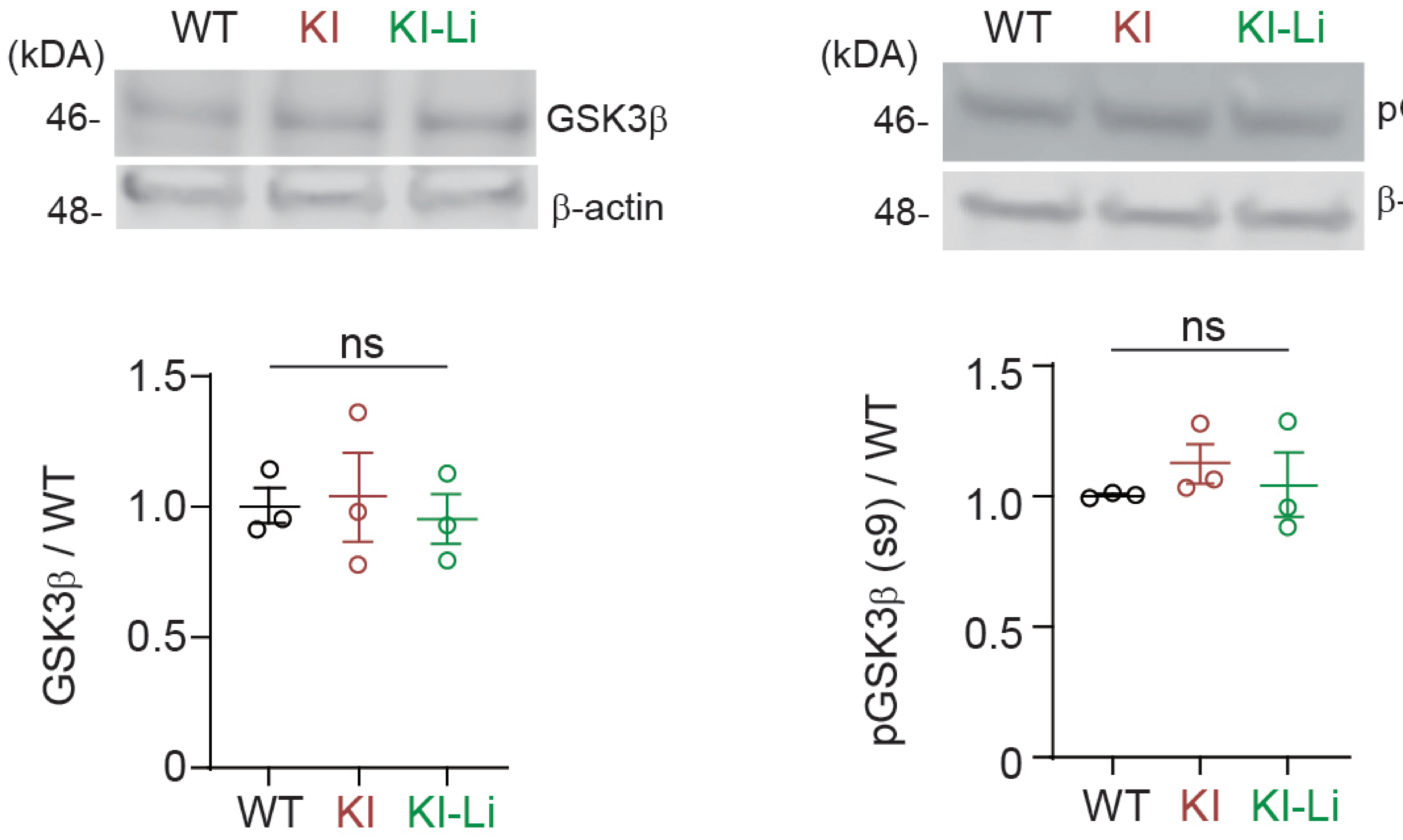

b

GSK3b S9

position:	-5	-4	-3	-2	-1	0	1	2	3	4	5	6
sequence:	R	P	R	T	T	S	F	A	E	S	C	K

Upstream kinase	Kinase group	%	Change in P21?	Change in P60?
DRAK1	CAMK	99.65%	ns	ns
PKN3	AGC	99.49%	ns	ns
PDK1	AGC	99.45%	ns	ns
TTBK2	CK1	99.34%	ns	ns
DCAMKL1	CAMK	99.32%	ns	ns
CAMK2A	CAMK	99.25%	ns	ns
SGK3	AGC	99.25%	ns	ns
AMPKA2	CAMK	99.21%	ns	ns
P70S6K	AGC	99.14%	ns	ns
DCAMKL2	CAMK	98.99%	ns	ns

c

P21 Upstream kinase groups for top 31 downregulated terms

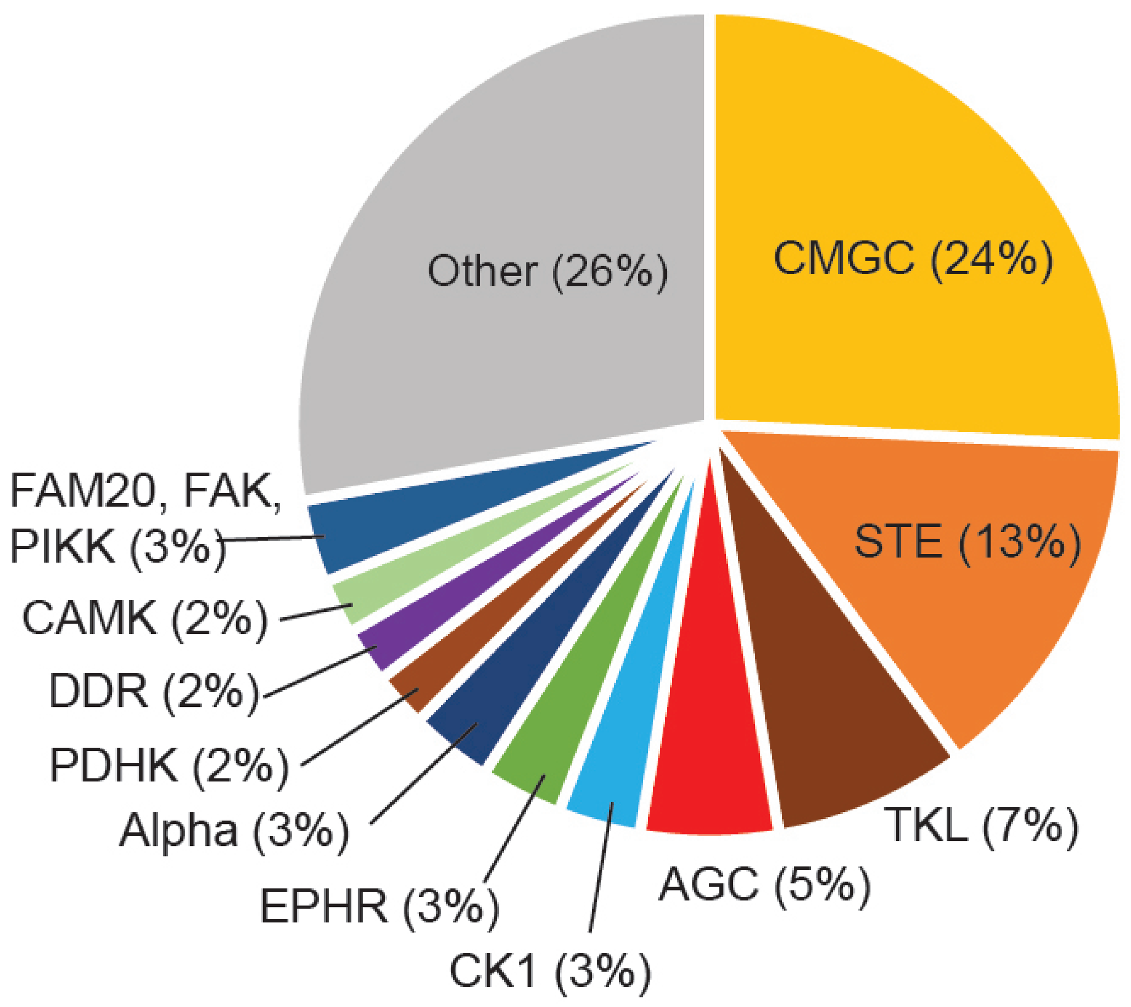

Top candidate substrates of Dyrk1a (D) / GSK3b (G) **P21**

DEPP	Site	%
Kalrn-7	S488	D 99.50%<br>G 88.40%
Spindoc	S249	D 89.86%<br>G 79.85%
Zc3h3	S920	D 83.18%<br>G 69.78%
Cd2bp2	T244	D 82.38%<br>G 67.63%
Tubb2a	S172	D 77.12%<br>G 80.21%
Aff4	S698	D 74.12%<br>G 95.21%
Bcas1	S414	D 73.69%<br>G 51.53%
Elavl2	S221	D 61.21%<br>G 68.14%

d

P60 Upstream kinase groups for top 22 downregulated terms

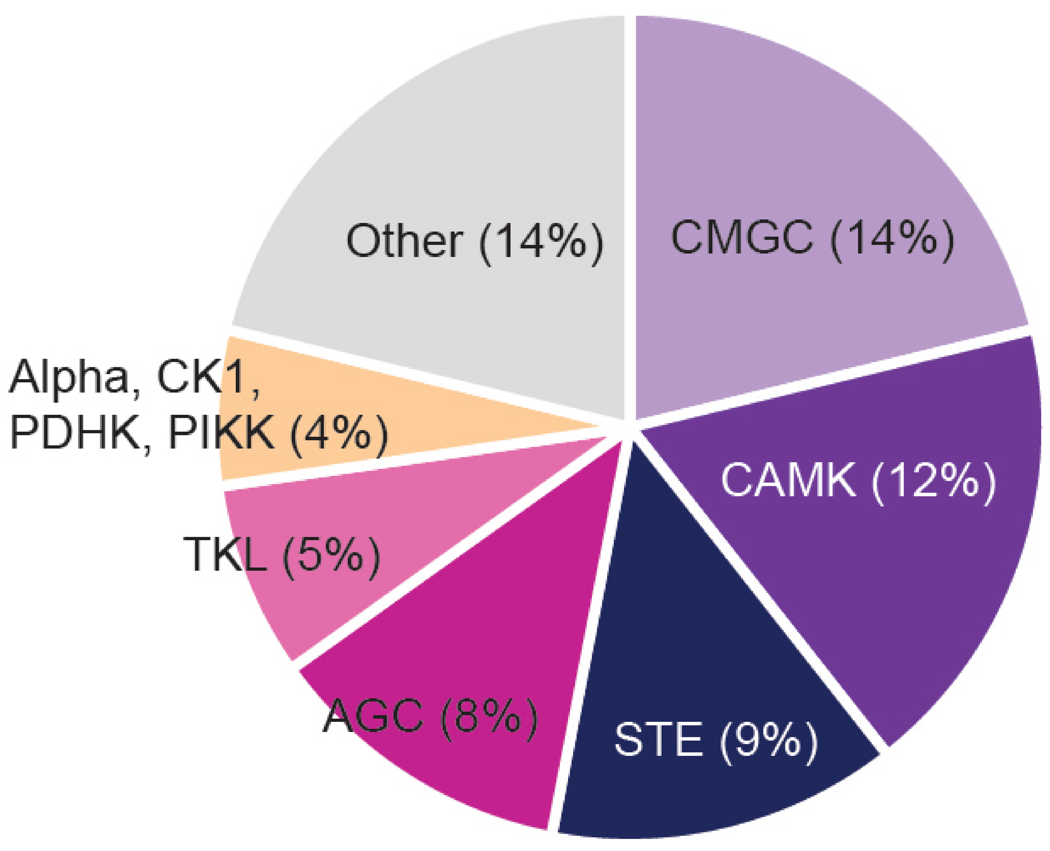

Top candidate substrates of Dyrk1a (D) / GSK3b (G) **P60**

DEPP	Site	%
Camsap2	S647	D 95.32%<br>G 76.98%
Prrc2c	S1965	D 94.95%<br>G 89.26%
Prr12	S1128	D 94.91%<br>G 83.56%
Znf827	S757	D 90.04%<br>G 75.23%
Kcnc1	S468	D 83.39%<br>G 87.08%
Acsbg1	S70	D 83.17%<br>G 51.14%
Ndrg2	S332	D 80.36%<br>G 50.18%
Tsc1	T640	D 44.94%<br>G 87.91%

e

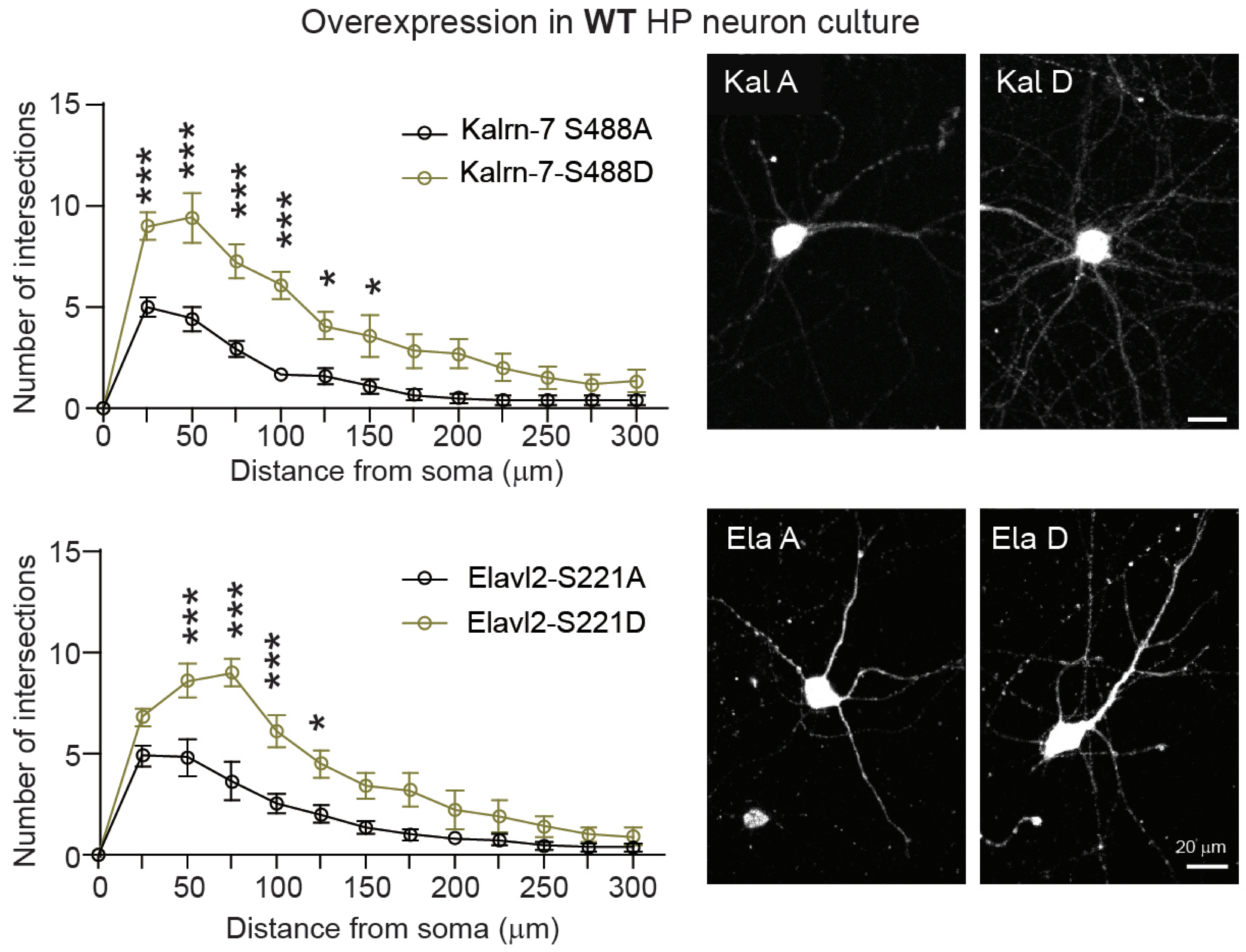

f

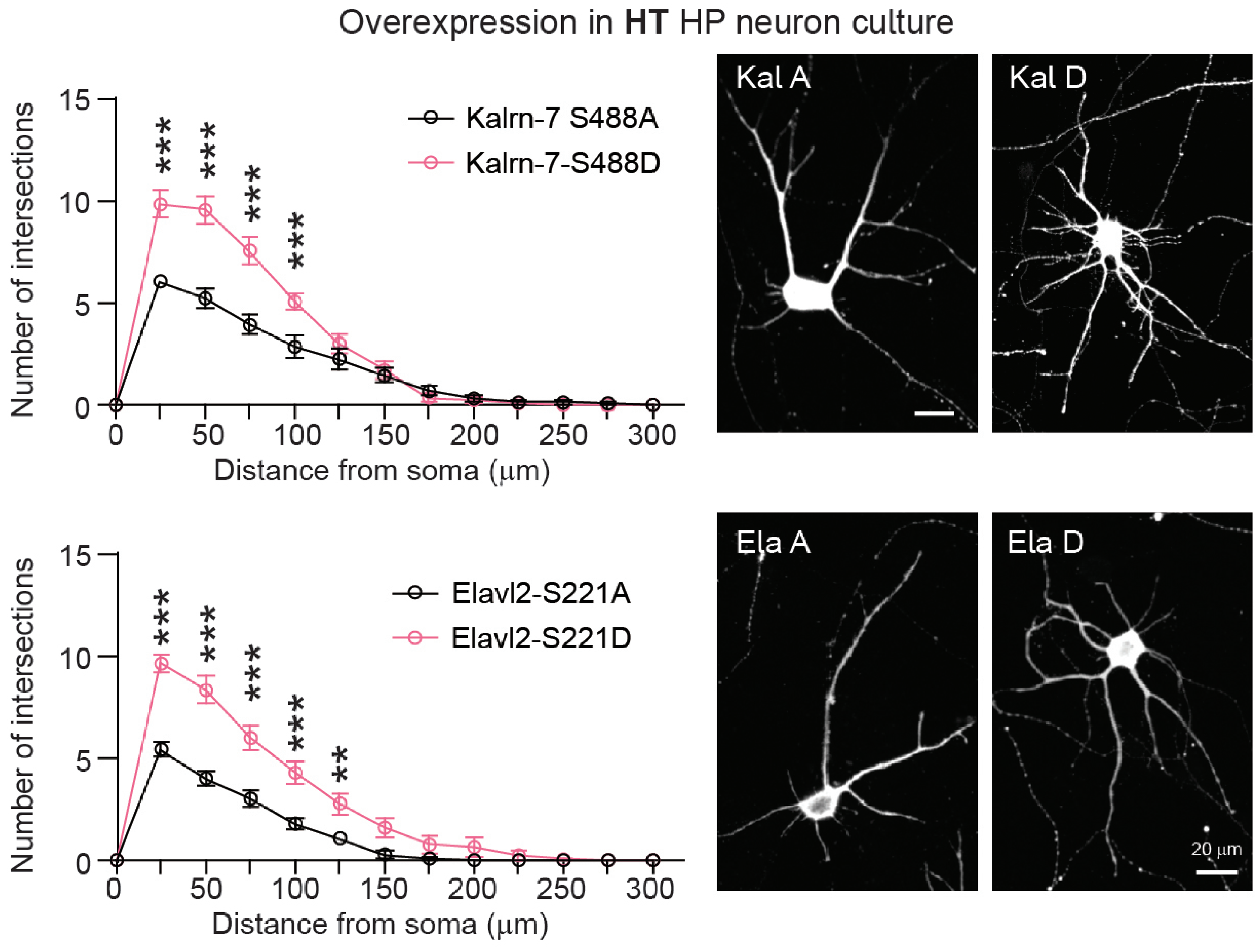

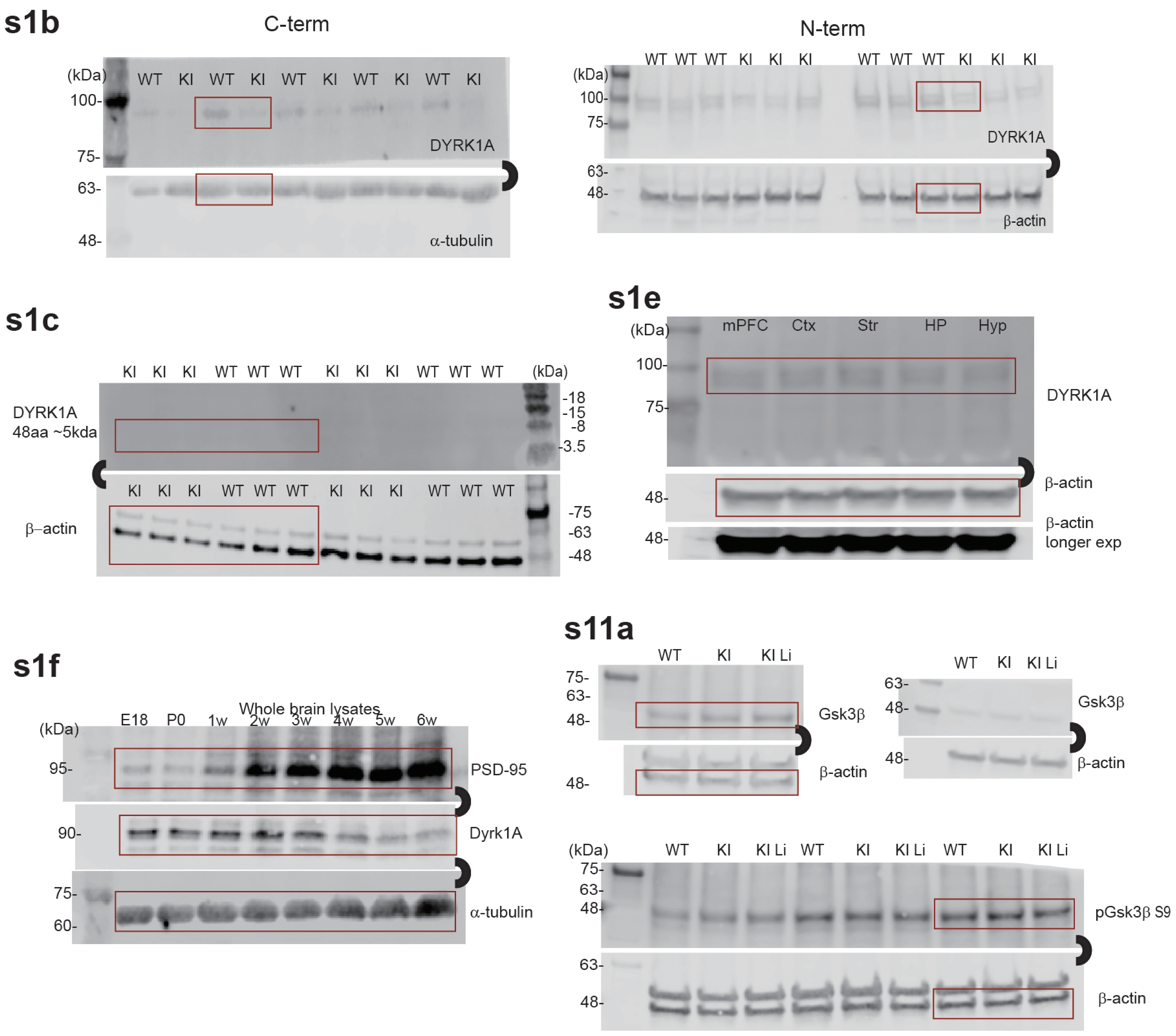
s1b
C-term
(kDa)
WT KI WT KI WT KI WT KI WT KI
100-
75-
DYRK1A
63-
48-
α-tubulin
N-term
WT WT WT KI KI KI WT WT WT KI KI KI
(kDa)
100-
75-
DYRK1A
63-
48-
β-actin
s1c
KI KI KI WT WT WT KI KI KI WT WT WT (kDa)
DYRK1A
48aa ~5kda
-18
-15
-8
-3.5
KI KI KI WT WT WT KI KI KI WT WT WT
β-actin
-75
-63
-48
s1e
(kDa)
mPFC Ctx Str HP Hyp
100-
75-
DYRK1A
48-
β-actin
48-
β-actin
longer exp
s1f
Whole brain lysates
(kDa)
E18 P0 1w 2w 3w 4w 5w 6w
95-
PSD-95
90-
Dyrk1A
75-
60-
α-tubulin
s11a
WT KI KI Li
75-
63-
48-
Gsk3β
48-
β-actin
WT KI KI Li
63-
48-
Gsk3β
48-
β-actin
(kDa)
WT KI KI Li WT KI KI Li WT KI KI Li
75-
63-
48-
pGsk3β S9
63-
48-
β-actin
